# Automated CUT&Tag profiling of chromatin heterogeneity in mixed-lineage leukemia

**DOI:** 10.1038/s41588-021-00941-9

**Published:** 2021-10-18

**Authors:** Derek H. Janssens, Michael P. Meers, Steven J. Wu, Ekaterina Babaeva, Soheil Meshinchi, Jay F. Sarthy, Kami Ahmad, Steven Henikoff

**Affiliations:** 1 Basic Sciences Division, Fred Hutchinson Cancer Research Center, Seattle, WA USA; 2 Molecular Engineering and Sciences Institute, University of Washington, Seattle, WA USA; 3 Clinical Research Division, Fred Hutchinson Cancer Research Center, Seattle, WA USA; 4 Cancer and Blood Disorder Center, Seattle Children’s Hospital, Seattle, WA USA; 5 Howard Hughes Medical Institute, Chevy Chase, MD USA

**Keywords:** Acute lymphocytic leukaemia, Acute myeloid leukaemia

## Abstract

Acute myeloid and lymphoid leukemias often harbor chromosomal translocations involving the *KMT2A* gene, encoding the KMT2A lysine methyltransferase (also known as mixed-lineage leukemia-1), and produce in-frame fusions of KMT2A to other chromatin-regulatory proteins. Here we map fusion-specific targets across the genome for diverse KMT2A oncofusion proteins in cell lines and patient samples. By modifying CUT&Tag chromatin profiling for full automation, we identify common and tumor-subtype-specific sites of aberrant chromatin regulation induced by KMT2A oncofusion proteins. A subset of KMT2A oncofusion-binding sites are marked by bivalent (H3K4me3 and H3K27me3) chromatin signatures, and single-cell CUT&Tag profiling reveals that these sites display cell-to-cell heterogeneity suggestive of lineage plasticity. In addition, we find that aberrant enrichment of H3K4me3 in gene bodies is sensitive to Menin inhibitors, demonstrating the utility of automated chromatin profiling for identifying therapeutic vulnerabilities. Thus, integration of automated and single-cell CUT&Tag can uncover epigenomic heterogeneity within patient samples and predict sensitivity to therapeutic agents.

## Abstract (web summary)

Automated and single-cell CUT&Tag is used to characterize the effects of KMT2A fusion proteins on chromatin in human primary leukemia samples, identifying oncogenic networks and fusion-specific therapeutic vulnerabilities.

## Main

Ten percent of acute leukemias harbor chromosomal translocations involving the *KMT2A* gene encoding lysine methyltransferase 2A (also referred to as mixed-lineage leukemia-1)^1^. In its normal role, KMT2A catalyzes methylation of the Lys4 residue of the histone H3 nucleosome tail (H3K4) and is required for fetal and adult hematopoiesis^2^. The N-terminal portion of KMT2A contains a low-complexity domain that mediates protein–protein interactions, an AT-hook/CXXC domain that binds DNA and multiple chromatin-interacting domains (PHD domains and a bromo domain), whereas the C-terminal portion contains a transactivation domain that interacts with histone acetyltransferases and a SET domain that catalyzes histone H3K4 methylation^3–6^. The KMT2A precursor protein is cleaved to a 320-kDa N-terminal fragment (KMT2A-N) and a 180-kDa C-terminal fragment (KMT2A-C) that form a stable dimer^7,8^.

*KMT2A* contributes to leukemogenesis through oncogenic chromosomal rearrangements involving the DNA-binding domain in the N-terminal portion of KMT2A with a diverse array of other chromatin-regulatory proteins^9,10^. Although more than 80 translocation partners have been identified in *KMT2A*-rearranged (*KMT2A*r) leukemias, fusions involving the AF9, ENL, ELL, AF4 and AF10 transcriptional elongation factors account for the majority of cases^1,10^. These fusion partners regulate RNA polymerase II elongation (ELL and AF4), recruit the DOT1L H3K79 histone methyltransferase (AF10), or both (AF9 and ENL)^11–14^. Additionally, ENL and AF9 interact with the CBX8 chromobox protein to neutralize the Polycomb repressive complex 1 (PRC1) gene-silencing complex^15,16^.

Previous work has suggested that KMT2A fusion proteins bind different genomic loci depending on the fusion partner to drive different leukemia subtypes^17,18^. For example, AF4 fusions are more common in acute lymphoid leukemia (ALL), and AF9 fusions are associated with acute myeloid leukemia (AML)^1^. In addition, *KMT2A* rearrangements are also prevalent in mixed-phenotype acute leukemia (MPAL), and numerous examples of *KMT2A*r leukemias that interconvert between lineage types have been documented^17,19–21^. However, because methods for efficiently and reliably profiling KMT2A fusion-binding sites in scarce input patient samples are lacking, the relationship among KMT2A fusions, chromatin structure and lineage plasticity has been challenging to fully characterize. Here we establish a chromatin profiling platform that efficiently profiles oncogenic fusion proteins, transcription-associated complexes and histone modifications in cell lines and patient samples. By integrating these results with findings from related single-cell methods, we characterize the regulatory dynamics of *KMT2A*r leukemias. We identify groups of target genes for fusion oncoproteins that show divergent patterns of active and repressive chromatin within the same sample. These patterns suggest that KMT2A fusion proteins activate distinct oncogenic networks within different cells of the same tumor and may explain the lineage plasticity associated with *KMT2A*r leukemia. In addition, we find that distinct fusion partners display different affinity for various transcriptional cofactors, which predicts cancer sensitivity to therapeutic compounds.

## Results

### Mapping the binding sites of diverse KMT2A fusion proteins

**Fig. 1:**
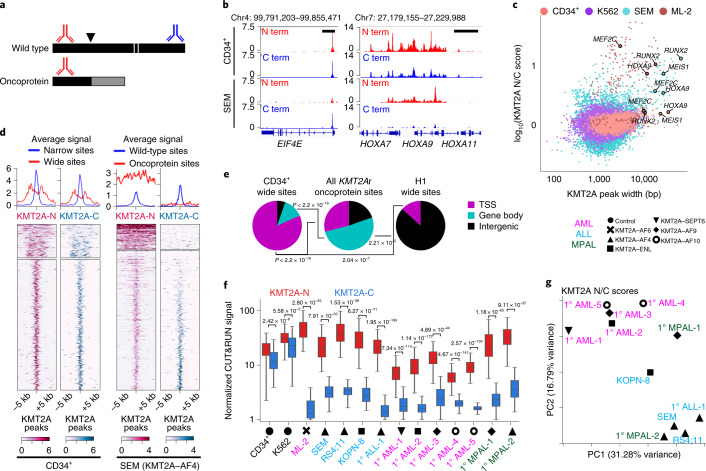
AutoCUT&RUN profiling of KMT2A fusion protein binding. **a**, A general strategy for mapping KMT2A fusion proteins. The wild-type KMT2A protein (black) is cleaved (white lines) into KMT2A-N and KMT2A-C proteins. Common oncogenic lesions (black arrowhead) produce in-frame translation of oncogenic KMT2A with numerous fusion partners (gray). Antibodies to C-terminal KMT2A (blue) recognize wild-type KMT2A-C. Antibodies to N-terminal KMT2A (red) recognize wild-type KMT2A-N and oncogenic KMT2A fusion proteins. **b**, Example of a wild-type KMT2A-binding site (*EIF4E*) and an oncoprotein-binding site (*HOXA* locus). Black scale bars, 10 kb. **c**, Scatterplot comparing KMT2A peak width and relative enrichment of the KMT2A N versus C terminus in control (CD34^+^ and K562) samples and *KMT2A*r (SEM and ML-2) samples. **d**, Heatmap comparison of KMT2A signal over 10-kb windows centered on wide KMT2A-binding sites (top) and narrow KMT2A-binding sites (bottom) in CD34^+^ HSPCs and KMT2A fusion protein-binding sites (top) and wild-type KMT2A-binding sites (bottom) in SEM cells. **e**, Pie charts showing the fraction of wide KMT2A peaks (CD34^+^ and H1 cells) and KMT2A fusion-bound sites (*KMT2A*r samples) overlapping transcriptional start sites (TSSs), gene bodies and intergenic regions. *P* values were computed using Fisher’s exact test. **f**, Box plots of KMT2A-N and KMT2A-C signal showing that the antibody to N-terminal KMT2A is enriched relative to the antibody to the C-terminal portion of KMT2A at fusion-binding sites. The center line indicates the median, box limits represent the first and third quartiles, and whiskers show all data within 1.5 times the interquartile range (IQR) of the lower and upper quartiles; outliers are not shown. *P* values were computed using a two-sample *t* test (two sided). CD34^+^, *n* = 131; K562, *n* = 65; ML-2, *n* = 144; SEM, *n* = 91; RS4;11, *n* = 92; KOPN-8, *n* = 192; 1° ALL-1, *n* = 156; 1° AML-1, *n* = 349; 1° AML-2, *n* = 423; 1° AML-3, *n* = 103; 1° AML-4, *n* = 270; 1° AML-5, *n* = 186; 1° MPAL-1, *n* = 248; 1° MPAL-2, *n* = 189. **g**, Principal-component analysis (PCA) of fusion oncoprotein-binding sites in *KMT2A*r samples. The first two components are shown.

**Extended Data Fig. 1:**
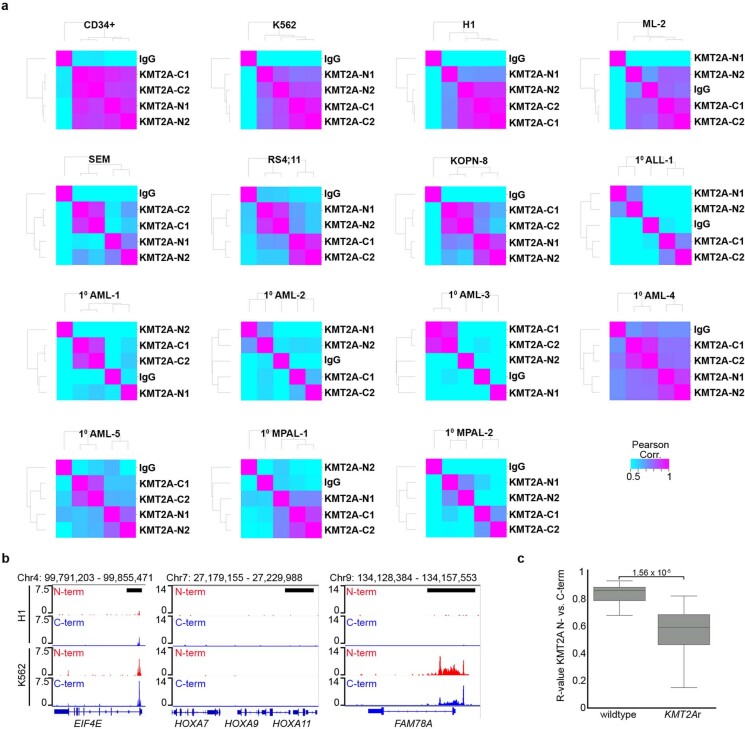
KMT2A N-terminus and C-terminus specific antibodies for AutoCUT&RUN chromatin profiling. **a**, Pearson correlation matrices between KMT2A N-terminus and C-terminus specific antibodies over the merged KMT2A peaks for each sample. In the control CD34 + progenitors, as well as K562 and H1 cells signals for the KMT2A-N1 antibody (Millipore Cat# 05-764), KMT2A-N2 antibody (Cell Signaling Tech Cat# 14689 S), KMT2A-C1 antibody (Millipore Cat# 05-765) and KMT2A-C2 antibody (Santa Cruz Cat# sc-374392) are all highly correlated, indicating that the N-terminal and C-terminal regions of wild-type KMT2A co-localize on chromatin. In the *KMT2A*r sample profiles the N-terminal antibodies show higher correlations with one another than they do with the C-terminal antibodies, indicating the KMT2A fusion binding is detected by the N-terminal antibodies and uncoupled from the KMT2A wild-type protein mapped by the C-terminal antibodies. **b**, Genome browser tracks showing wild-type KMT2A enrichment over the TSS of the *EIF4E* gene in both H1 and K562 cells. In contrast to CD34 + HSPCs, many critical hematopoietic cell fate determinants (for example *HOXA9*) are not bound by wild-type KMT2A in H1 or K562 cells. A broad distribution of KMT2A signal is found across the gene bodies of a limited collection of target genes (for example *FAM78A*) in K562 cells. Black scale bars = 10 kb. **c**, The Pearson correlation of KMT2A N- versus C-terminal profiles is significantly higher in the control KMT2A wild-type samples than in the *KMT2A*r samples. Center line = median; box limits = first and third quartiles; whiskers show all data within 1.5 IQRs of the lower and upper quartiles; outliers are not shown; *P* value was computed using a two sample t-test (two-sided); wildtype, *n* = 12; *KMT2A*r, *n* = 48.

Characterizing the chromatin localization of oncogenic fusion proteins has often been limited by the inability of chromatin immunoprecipitation and sequencing (ChIP–seq) to be used with small amounts of patient samples. To efficiently compare the binding sites for wild-type KMT2A and fusion proteins, we applied AutoCUT&RUN^22^ across a panel of four *KMT2A*r leukemia cell lines and eight primary *KMT2A*r patient samples sorted for CD45^+^ blasts. This collection spans the spectrum of *KMT2A*r leukemia subtypes with diverse *KMT2A* translocations that create oncogenic fusion proteins with the transcriptional elongation factors AF4 (SEM, RS4;11, 1° ALL-1, 1° MPAL-2), AF9 (1° AML-3, 1° MPAL-1), ENL (KOPN-8, 1° AML-2), AF6 (ML-2) and AF10 (1° AML-4, 1° AML-5) as well as a relatively rare fusion to the cytoplasmic GTPase SEPT6 (1° AML-1) (Supplementary Table 1). With the exception of ML-2, an AML-derived cell line, these samples also contained a wild-type copy of the *KMT2A* locus. For comparison, we also profiled KMT2A localization in untransformed human CD34^+^ hematopoietic stem and progenitor cells (HSPCs), in H1 human embryonic stem cells and in the K562 leukemia cell line, each of which lacks *KMT2A* translocations. Antibodies to the C-terminal portion recognized only wild-type KMT2A-C, while antibodies to the N-terminal portion recognized both wild-type KMT2A-N and the fusion proteins (Fig. 1a). Therefore, binding sites unique to oncogenic fusion proteins could be identified by comparing the chromatin profiles obtained with antibodies specific to C-terminal and N-terminal KMT2A. We used AutoCUT&RUN to profile replicate samples with two different antibodies to the N terminus and two different antibodies to the C terminus of KMT2A, and correlation analysis of the sequencing results showed high reproducibility (*r* = 0.78 ± 0.19; Extended Data Fig. 1a).

**Extended Data Fig. 2:**
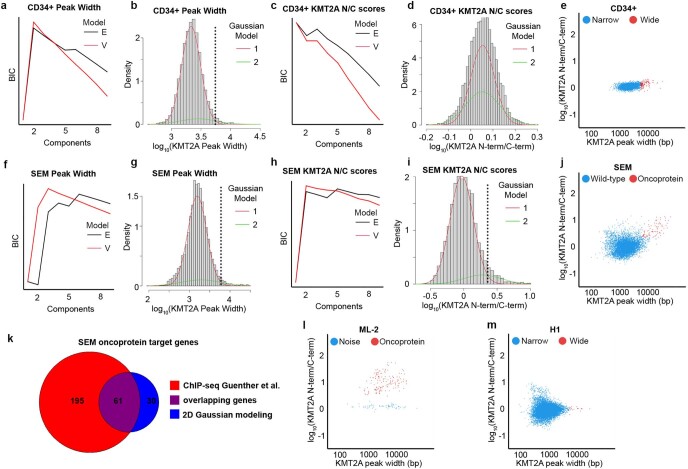
A uniform approach to identify sites bound by the KMT2A fusion protein using KMT2A N- and C-terminal profiles. **a**, Line plot comparing the Bayesian Information Criterion (BIC) for a range of Gaussian mixture models containing 1-9 components using either equal (E) or unequal (V) variance to model the distribution of KMT2A peak widths in CD34 + HSPCs. A two component Gaussian mixture model provides the highest BIC. **b**, Histogram of KMT2A peak widths in CD34 + HSPCs showing the two Gaussian models fit to the data. The dotted line indicates the threshold separating peaks called as ‘wide’ versus ‘narrow’. **c**, Same as (a) but modeling the distribution of the relative enrichment of the KMT2A N- versus C-terminus (KMT2A N/C scores) over KMT2A peaks in CD34 + HSPCs. The highest BIC is achieved by a single Gaussian distribution. **d**, A two component model fails to partition the KMT2A peaks by N/C scores in CD34 + HSPCs. **e**, Scatter plots comparing the KMT2A peak width and N/C scores in control CD34 + HSPCs; wide peaks indicated in red. **f**, Same as (a) but for the *KMT2A*r SEM cell line. **g**, Same as (b) but for SEM cells. **h**, Same as (c) but for SEM cells. Here, a two component Gaussian mixture model achieves the highest BIC. **i**, Two Gaussian models fit to the SEM KMT2A N/C scores. The dotted line indicates the threshold separating peaks with ‘high’ versus ‘low’ KMT2A N/C scores. **j**, Same as (e) but for SEM cells; oncoprotein targets are indicated in red. **k**, Venn diagram of KMT2A-AF4 oncoprotein target genes in SEM cells called using either this two-dimensional Gaussian modeling approach or using ChIP-seq^23^. **l**, Same as (e) for the *KMT2A*r ML-2 cell line which lacks the wild-type *KMT2A* allele. **m**, Same as (e) for the control H1 sample.

To evaluate our KMT2A dual-antibody approach, we first compared the KMT2A N-terminal and C-terminal profiles between our KMT2A wild-type and *KMT2A*r samples. As expected, in H1 and K562 cells and CD34^+^ HSPCs, KMT2A-N and KMT2A-C showed nearly identical patterns of enrichment across the genome (*r* = 0.82 ± 0.08; Fig. 1b and Extended Data Fig. 1a,b). Strikingly, in H1 cells, KMT2A binding was generally focused in narrow peaks directly over TSSs, whereas in K562 cells and CD34^+^ progenitors additional regions showed wide peaks of both KMT2A-N and KMT2A-C extending from TSSs across gene bodies. Many of the genes with a wide KMT2A distribution in CD34^+^ progenitors (for example, *HOXA9*, *RUNX2*, *MEIS1*, *MEF2C*) are master regulators of hematopoietic cell fate (Fig. 1c) and have previously been defined as KMT2A fusion oncoprotein targets in leukemias^18,23^. In the *KMT2A*r leukemia samples, the correlation between the KMT2A N-terminal and C-terminal profiles was significantly lower than in the control samples (*r* = 0.53 ± 0.21; Extended Data Fig. 1a,c), and many of the wide KMT2A-bound regions showed an enriched KMT2A N-terminal signal relative to the KMT2A C terminus (Fig. 1b,c). To systematically define fusion protein-binding sites across our collection of samples, we used Gaussian mixture modeling to partition KMT2A peaks into two different distributions based on both the width of the KMT2A peaks and the enrichment-normalized ratio of KMT2A-N to KMT2A-C signal (KMT2A N/C score; Fig. 1c and Extended Data Fig. 2). In CD34^+^ HSPCs, 131 of 6,336 KMT2A-bound sites were called as wide peaks (mean = 8.2 ± 4 kb) and a two-component Gaussian mixture model failed to partition the KMT2A-bound sites based on N/C score (Fig. 1c,d and Extended Data Fig. 2a–e), suggesting that there is similar enrichment of KMT2A-N and KMT2A-C proteins, consistent with wild-type KMT2A binding. In comparison, in the SEM cell line encoding a KMT2A–AF4 fusion, 195 of 8,259 KMT2A-bound regions were called as wide (mean = 13.1 ± 10 kb), and about half of these wide peaks (91/195) were enriched for KMT2A-N relative to KMT2A-C, which we interpret as fusion oncoprotein-binding sites (Fig. 1c,d and Extended Data Fig. 2f–j). In line with this interpretation, 61 of 91 of the oncoprotein target genes we identified in SEM cells overlapped with KMT2A–AF4 target genes that were previously identified in SEM cells using ChIP–seq^23^ (Extended Data Fig. 2k). The ML-2 cell line has a deletion of the wild-type *KMT2A* allele and harbors only a KMT2A–AF6 fusion oncoprotein. As expected, the majority of KMT2A-bound sites had a high KMT2A N/C score (144/211; Fig. 1c and Extended Data Fig. 2l). This persistent localization of the KMT2A–AF6 fusion oncoprotein to chromatin in ML-2 cells demonstrates that binding of the oncoprotein is not dependent on wild-type KMT2A.

**Extended Data Fig. 3:**
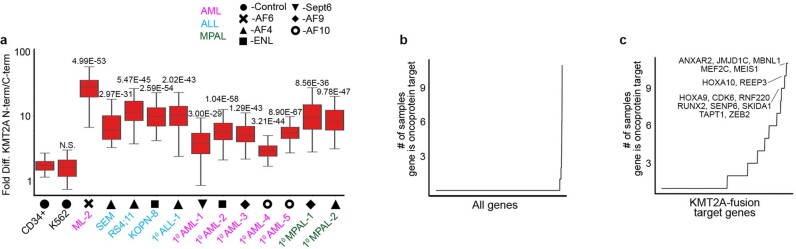
Comparison of fusion oncoprotein binding sites across all samples. **a**, Box plot showing the fold difference between the KMT2A-N and -C signal at wide KMT2A peaks in the control CD34 + and K562 samples is significantly less than at oncoprotein target sites in the *KMT2A*r samples. Center line = median; box limits = first and third quartiles; whiskers show all data within 1.5 IQRs of the lower and upper quartiles; outliers are not shown; *P* values were computed using a two sample t-test (two-sided) comparing the fold difference between the KMT2A-N and -C signal on KMT2A fusion oncoprotein sites to wide KMT2A peaks in the CD34 + control; CD34 + , *n* = 131; K562, *n* = 65; ML-2, *n* = 144; SEM, *n* = 91; RS4;11, *n* = 92; KOPN-8, *n* = 192; 1^0^ ALL-1, *n* = 156; 1^0^ AML-1, *n* = 349; 1^0^ AML-2, *n* = 423; 1^0^ AML-3, *n* = 103; 1^0^ AML-4, *n* = 270; 1^0^ AML-5, *n* = 186; 1^0^ MPAL-1, *n* = 248; 1^0^ MPAL-2, *n* = 189. **b**, Line plot of all genes showing the number of samples in which a particular gene is called as an oncoprotein target. **c**, Same as (b) but only genes called as an oncoprotein target in at least one sample are included. The most frequent oncoprotein target genes are indicated.

Next, we examined how the distribution of KMT2A differed between wild-type and oncofusion proteins. In CD34^+^ HSPCs, 81% of wide peaks overlapped a gene TSS, whereas in *KMT2A*r samples significantly fewer fusion oncoprotein peaks overlapped a TSS (30%), and significantly more (50%) overlapped a gene body (Fig. 1e). In comparison, in the control H1 human embryonic stem cell line, only 15 of 17,000 KMT2A peaks were called as wide and these peaks were significantly less enriched on gene TSSs than the wide KMT2A peaks we identified in CD34^+^ HSPCs and significantly less enriched in gene bodies than the oncoprotein-binding sites in *KMT2A*r leukemia samples (Fig. 1e and Extended Data Fig. 2m). This pattern of KMT2A fusion oncoproteins spreading across target gene bodies is consistent with previous reports^23,24^. By comparing enrichment of the KMT2A N terminus and C terminus across fusion oncoprotein-binding sites in all *KMT2A*r samples, we found that in all cases the N terminus was significantly more enriched than the C terminus (Fig. 1f), and in all cases the fold difference between the N-terminal and C-terminal signal at oncoprotein-bound regions in *KMT2A*r samples was greater than in the wide KMT2A-bound regions in CD34^+^ HSPCs (Extended Data Fig. 3a). Taking these findings together, we conclude that our KMT2A N-terminal versus C-terminal antibody multiplexing approach identifies regions bound by diverse KMT2A fusion oncoproteins.

We then compared oncoprotein target sites among leukemias with different KMT2A fusions. We found that 81 of 440 (~18%) of all fusion oncoprotein target genes were shared by five or more of the *KMT2A*r leukemia samples we profiled, representing 12% of the total sequence space occupied by the fusion proteins (Extended Data Fig. 3b,c). As expected, the group of genes we identified as the most frequent KMT2A fusion targets across our collection of samples included several genes that are known to be required for *KMT2A*r leukemia, including *HOXA9*, *MEIS1*, *MEF2C*, *MBNL1* and *JMJD1C*^25–29^ (Extended Data Fig. 3c and Supplementary Table 2). PCA of KMT2A N/C scores across all oncoprotein-binding sites indicated that both the specific fusion partner and the myeloid versus lymphoid lineage bias of the tumor may influence tumor-specific localization of the oncofusion protein (Fig. 1g). For example, all KMT2A–AF4 samples clustered together in the PCA plot and grouped with a sample from a patient with ALL and a sample from a patient with MPAL. By contrast, the ALL cell line KOPN-8 carries a KMT2A–ENL fusion protein and partitioned away from KMT2A–AF4-bearing leukemias. Primary AML samples bearing KMT2A–AF9, KMT2A–AF10 and KMT2A–ENL fusions formed a second cluster, apart from the KMT2A–SEPT6-containing primary AML and the primary KMT2A–AF9-bearing MPAL sample. Thus, tumors bearing KMT2A–AF4 fusions share a distinct binding profile, but other oncofusion proteins such as KMT2A–ENL and KMT2A–AF9 also appear to be influenced by the lineage bias of the tumor.

### Chromatin landscape of *KMT2A*r leukemia samples

**Extended Data Fig. 4:**
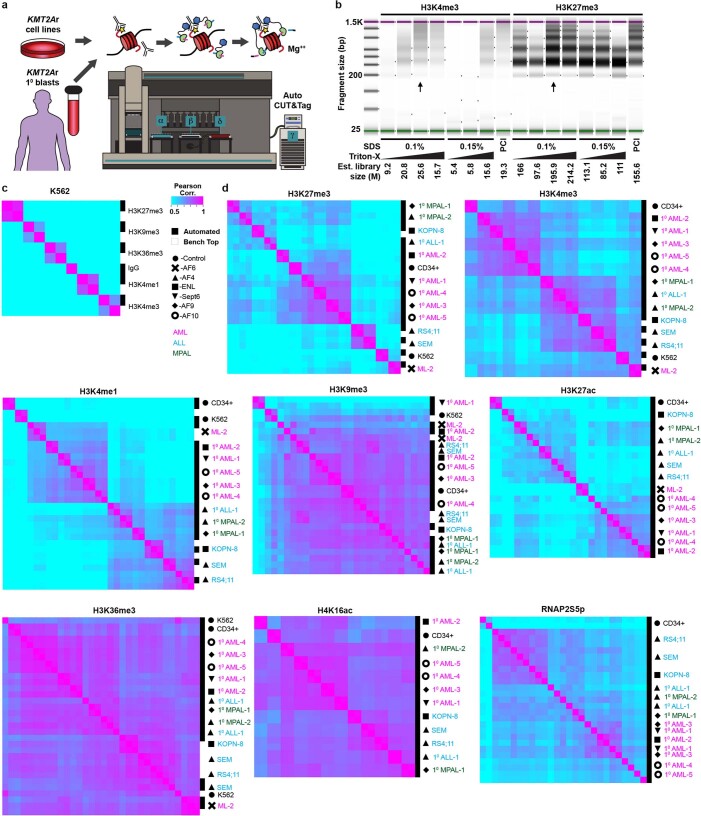
Adaptation of CUT&Tag for full automation. **a**, Con-A bound nuclei are incubated with the primary antibody of interest and arrayed for AutoCUT&Tag profiling on a liquid handling robot equipped for high volume magnetic separation (α), low volume magnetic separation (β) and temperature control (δ, γ). This method prepares up to 96 sequencing-ready samples in a single day that can all be pooled on a single HiSeq two-lane or comparable flow cell for sequencing. The automated protocol uses a low concentration of SDS to displace bound Tn5 from tagmented DNA, and Triton-X100 to quench the detergent for PCR. **b**, CUT&Tag libraries imaged on an Agilent Tapestation 4200 instrument, this High sensitivity D1000 screen tape image is uncropped. To optimize the DNA release and quenching conditions for AutoCUT&Tag varying amounts of SDS and Triton-X100 were tested for library yield. Arrows indicate the optimum condition. **c**, Pearson correlation matrix of reproducibility between benchtop and automated CUT&Tag profiling methods on K562 fixed nuclei with 5 antibodies to histone modifications as well as the IgG negative control antibody. The log-transformed signal was compared across 5-kb bins tiling the entire genome. **d**, Pearson correlation matrix of reproducibility between benchtop and automated CUT&Tag profiling methods on fixed nuclei from all 12 *KMT2A*r leukemias as well as the CD34 + progenitor and K562 control samples using antibodies against H3K27me3, H3K4me3, H3K4me1, H3K36me3, H3K9me3, H3K27ac, H4K16ac, and RNAP2S5p. Here, log-transformed signal was compared across the merged peak file of all samples for each mark. H3K27me3, H3K4me3, H3K4me1, H3K27ac, and RNAP2S5p show the highest variation between samples, and most reliably cluster sample replicates together.

To economically characterize the global chromatin landscape of tumors at a scale that could be generally applied to patient samples, we developed AutoCUT&Tag, a modification of our previous AutoCUT&RUN robotic platform^22^. CUT&Tag takes advantage of the high efficiency and low background of antibody-tethered Tn*5* tagmentation-based chromatin profiling relative to previous methods, such as ChIP–seq and CUT&RUN^30^. The standard CUT&Tag protocol requires DNA extraction before library enrichment by PCR. However, we recently developed conditions for DNA release and PCR enrichment without extraction (CUT&Tag-direct)^31^. In this improved protocol, a low concentration of SDS is used to displace bound Tn*5* from tagmented DNA, and subsequent addition of the non-ionic detergent Triton X-100 quenches the SDS to allow for efficient PCR. This streamlined protocol makes CUT&Tag compatible with robotic handling of samples in a 96-well plate format and generates profiles with data quality comparable to that produced by benchtop CUT&Tag (*r* = 0.79 ± 0.093; Extended Data Fig. 4a–c).

**Fig. 2:**
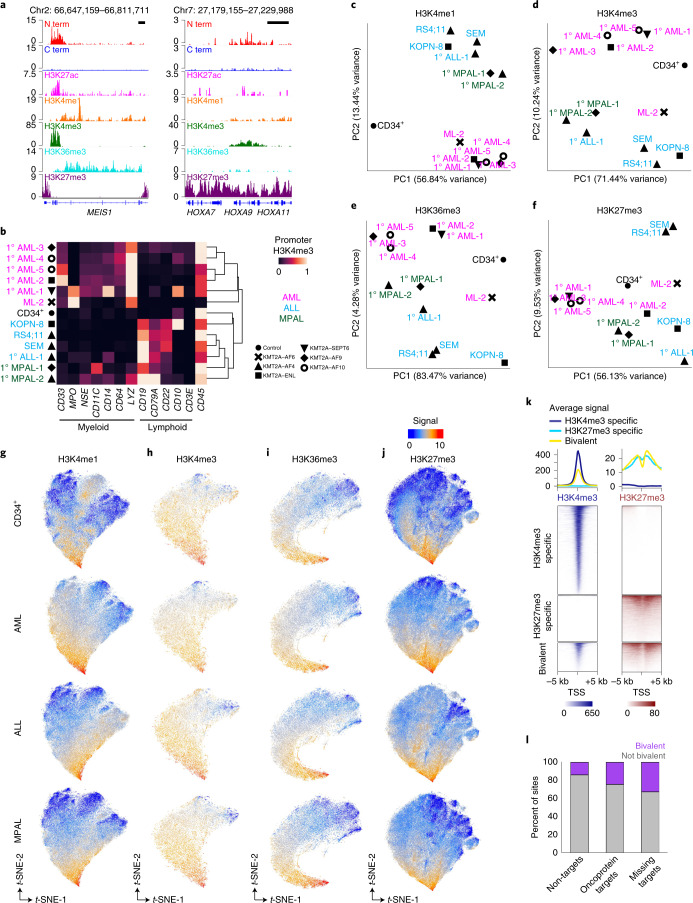
Clustering regulatory features distinguishes common and restricted elements in leukemia samples. **a**, The *MEIS1* locus is a direct target of KMT2A–AF9 in the 1° MPAL-1 sample and is decorated by both active and repressive chromatin marks. The *HOXA* cluster is relatively repressed in this tumor. Black scale bars, 10 kb. **b**, H3K4me3 signal at the promoters of diagnostic immunophenotypic markers accurately classifies AML, ALL and MPAL leukemias. **c**, PCA clustering analysis of H3K4me1-marked regions across the genome separates samples according to lineage specificity. **d**, Same as in **c** for H3K4me3. **e**, Same as in **c** for H3K36me3. **f**, Same as in **c** for H3K27me3. Grouping samples by PCA of H3K27me3-marked repressive chromatin separates tumors of the same lineage. **g**, Two-dimensional *t*-distributed stochastic neighbor embedding (*t*-SNE) projections of all H3K4me1-marked regions separates lineage-specific regulatory elements. Each colored pixel corresponds to a single H3K4me1 peak, colored by the maximum intensity in the indicated sample type. **h**, *t*-SNE projection of H3K4me3 identifies lineage-specific promoters. **i**, Same as in **g** for H3K36me3. **j**, Same as in **g** for H3K27me3. AML and ALL samples display widespread H3K27me3, whereas H3K27me3 is more confined in the CD34^+^ control and MPAL samples. **k**, Heatmaps showing H3K4me3- and H3K27me3-specific regions (top) as well as regions called as bivalent (bottom) in the 1° MPAL-1 sample. **l**, Comparison of bivalent chromatin at genes not bound by the KMT2A fusion (non-targets), genes bound by the KMT2A fusion (oncoprotein targets) and genes bound in the majority of samples but missed in the sample of interest (missing targets) shows that a bivalent chromatin signature is enriched at oncoprotein target genes.

To define the chromatin features around KMT2A fusion-binding sites, we used AutoCUT&Tag to profile the active chromatin modifications H3K4me1, H3K4me3, H3K36me3, H3K27ac and H4K16ac as well as initiating RNA polymerase II marked by Ser5 phosphorylation of the C-terminal domain (RNAP2S5p). In addition, we profiled the silencing histone modifications H3K27me3 and H3K9me3. Together, these eight modifications distinguish active promoters, enhancers, transcribed regions, developmentally silenced chromatin and constitutively silenced chromatin^32^ and provide a straightforward picture of the regulatory status of a genome (Fig. 2a). Replicate profiles for each mark in control CD34^+^ samples and *KMT2A*r leukemia samples were very similar and were merged for further analysis (H3K27me3, *r* = 0.93 ± 0.051; H3K4me3, *r* = 0.96 ± 0.015; H3K4me1, *r* = 0.90 ± 0.037; H3K9me3, *r* = 0.83 ± 0.060; H3K27ac, *r* = 0.80 ± 0.077; H3K36me3, *r* = 0.95 ± 0.021; H4K16ac, *r* = 0.97 ± 0.012; RNAP2S5p, *r* = 0.77 ± 0.107) (Extended Data Fig. 4d).

**Extended Data Fig. 5:**
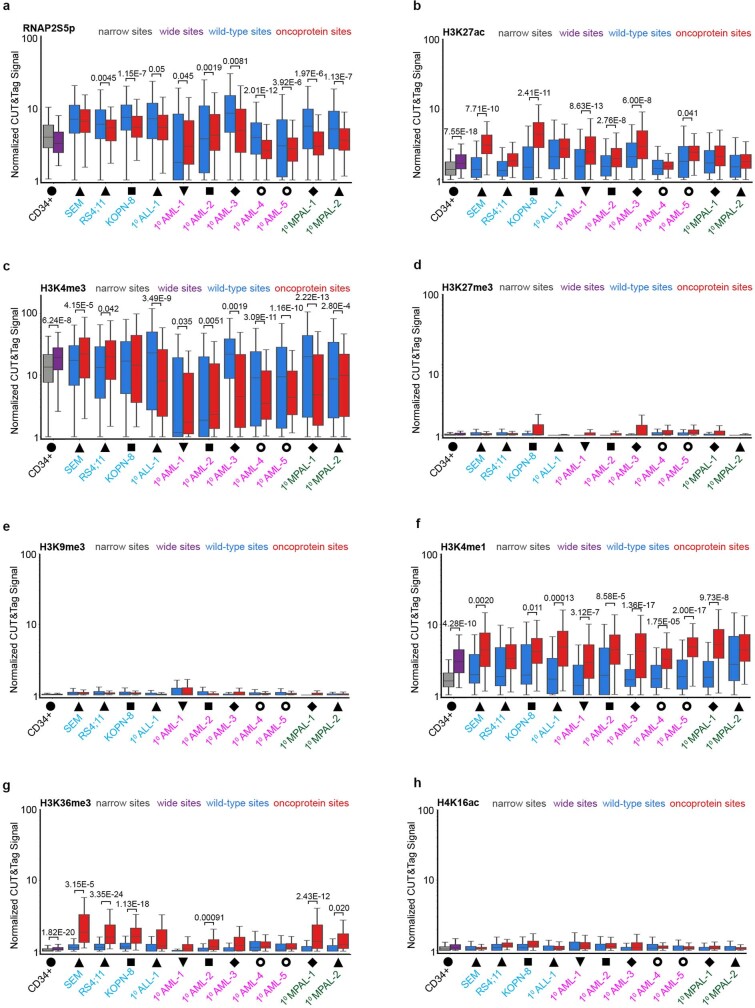
Chromatin features of KMT2A fusion protein binding sites. **a**, Quantification of the RNAP2Sp signal across the KMT2A wild-type and oncoprotein target sites in all samples. Signal is normalized by total coverage, as well as peak width. For all box plots center line = median; box limits = first and third quartiles; whiskers show all data within 1.5 IQRs of the lower and upper quartiles; outliers are not shown; *P* values were computed using a two sample t-test (two-sided). *N* values listed in order: narrow sites, wide sites for the control, or wild-type sites, oncoprotein sites for the *KMT2A*r samples. CD34 + , *n* = 6204, 131; SEM, *n* = 8168, 91; RS4;11, *n* = 10287, 92; KOPN-8, *n* = 9723, 192; 1^0^ ALL-1, *n* = 3747, 156; 1^0^ AML-1, *n* = 15915, 349; 1^0^ AML-2, *n* = 26148, 423; 1^0^ AML-3, *n* = 3943, 103; 1^0^ AML-4, *n* = 7218, 270; 1^0^ AML-5, *n* = 11256, 186; 1^0^ MPAL-1, *n* = 8641, 248; 1^0^ MPAL-2, *n* = 15179, 189. **b**, Same as (a) but for H3K27ac. H3K27ac is significantly enriched at the fusion oncoprotein binding sites in the SEM, KOPN-8, 1^0^ AML-1, 1^0^ AML-2, 1^0^ AML-3 and 1^0^ AML-5 *KMT2A*r leukemia samples as compared to the sample matched wild-type KMT2A bound sites. **c**, Same as (a) but for H3K4me3. H3K4me3 is significantly enriched at the fusion oncoprotein binding sites in SEM, RS4;11, 1^0^ AML-1, 1^0^ AML-2 and 1^0^ MAPL-2 and significantly depleted in 1^0^ ALL-1, 1^0^ AML-3, 1^0^ AML-4, 1^0^ AML-5 and 1^0^ MPAL-1. **d**, Same as (a) but for H3K27me3. **e**, Same as (a) but for H3K9me3. **f**, Same as (a) but for H3K4me1. **g**, Same as (a) but for H3K36me3. **h**, Same as (a) but for H4K16ac.

We first compared the chromatin features associated with sites bound by wild-type KMT2A to sites bound by KMT2A oncofusion proteins across all samples. In line with localization of the KMT2A fusion proteins to actively transcribed genes, we found that the active promoter marks H3K4me3, RNAP2S5p and H3K27ac were all present at oncofusion protein-binding sites (Extended Data Fig. 5a–c). H3K4me3 was also enriched at some promoters in the ML-2 cell line (for example, *LPO* and *LYZ* in Fig. 2b), which lacks the KMT2A methyltransferase domain, indicating that another H3K4me3 methyltransferase is responsible. In comparison to sample-matched wild-type KMT2A-bound sites, H3K27ac was enriched at oncofusion protein-binding sites in all samples, but this difference was statistically significant in only 6 of 11 samples (SEM, KOPN-8, 1° AML-1, 1° AML-2, 1° AML-3 and 1° AML-5; Extended Data Fig. 5b). The H3K4me3 mark was significantly enriched at oncofusion protein-binding sites in five of the samples (SEM, RS4;11, 1° AML-1, 1° AML-2 and 1° MPAL-2) and significantly depleted in five of the other samples (1° ALL-1, 1° AML-3, 1° AML-4, 1° AML-5 and 1° MPAL-1; Extended Data Fig. 5c). Oncofusion protein-binding sites lacked H3K27me3 and H3K9me3 (Extended Data Fig. 5d,e) but were enriched in H3K4me1 and H3K36me3, both of which mark transcribed gene bodies, and this enrichment was significant in 9 of 11 and 6 of 11 of the *KMT2A*r leukemia samples, respectively (Extended Data Fig. 5f,g). Enrichment of these marks is expected with mistargeting of KMT2A fusions to gene bodies^33^.

**Extended Data Fig. 6:**
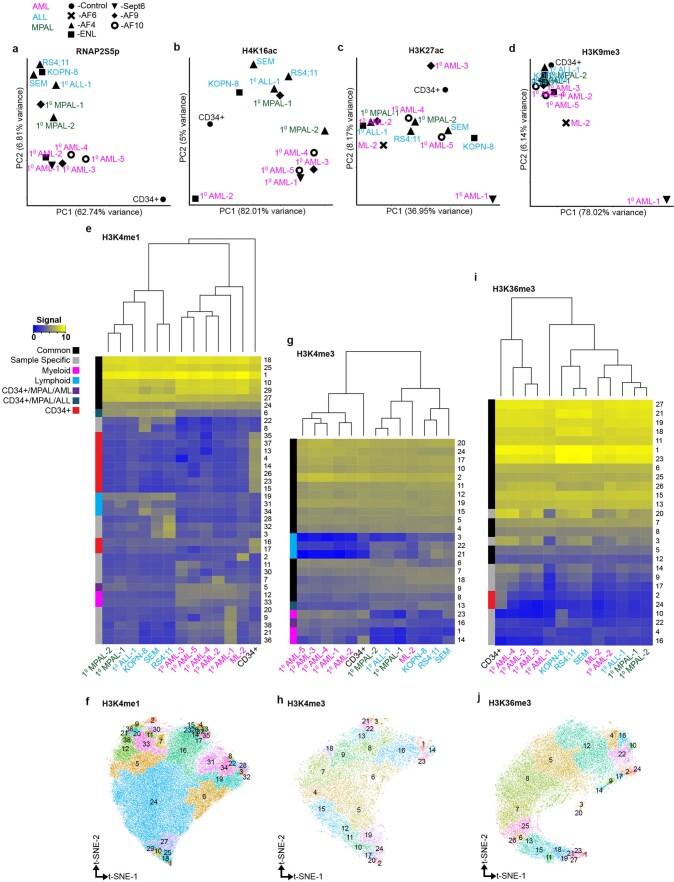
Grouping *KMT2A*r samples and regulatory regions according to the sample specific AutoCUT&Tag chromatin profiles. **a**, PCA of genome-wide RNAP2S5p signal separates samples by leukemia subtype. **b**, PCA of genome-wide H4K16ac signal separates samples by leukemia subtype. **c**, Samples organized by PCA of H3K27ac are not separated by leukemia subtype. **d**, PCA of genome-wide H3K9me3 signal only distinguishes the 1^0^ AML-1 KMT2A-Sept6 containing sample from the rest. **e**, Clustering analysis separates the H3K4me1-marked regions into 38 groups, and the heatmap shows the average signal intensity of H3K4me1 in each of these groups (y-axis) for each *KMT2A*r leukemia sample (x-axis). The colors alongside the heatmap show the subtype designation of each group as common across samples (black), myeloid (magenta), lymphoid (cyan), shared by CD34 + , MPAL and myeloid samples (plum), shared by CD34 + , MPAL and lymphoid samples (teal), CD34 + specific (red), or sample specific (gray). **f**, Two-dimensional t-SNE projections of all H3K4me1-marked regions as in Fig. 2g, but colored according to the group designation as indicated by the numbers along the right side of (e). **g**, Same as (e) for H3K4me3. Clustering analysis separates the H3K4me3-marked regions into 24 groups. **h**, Same as (f) for H3K4me3. **i**, Same as (e) for H3K36me3. Clustering analysis separates the H3K36me3-marked regions into 27 groups. **j**, Same as (f) for H3K36me3.

Histone modification profiling holds the potential to reveal similarities and distinctions between leukemias by reporting their transcriptional status. For example, H3K4me3 reports gene promoter activity and was enriched at marker genes that matched the immunophenotypic characterization of each leukemia (Fig. 2b). To determine how the global distribution of these marks varied between *KMT2A*r leukemia samples, we first identified regions enriched for each modification in our collection of *KMT2A*r leukemia samples as well as CD34^+^ HSPCs using the SEACR peak-calling method^34^ and performed PCA to cluster samples according to their modification-specific similarities. Overall, active chromatin features marked by H3K4me1, H3K4me3, H3K36me3, H4K16ac or RNAP2S5p clustered samples according to their ALL, AML or MPAL lineage designation (Fig. 2c–e and Extended Data Fig. 6a,b), suggesting that a similar repertoire of active genes is used in each leukemia subtype. By contrast, PCA based on H3K27ac or H3K27me3 CUT&Tag profiles partitioned samples into groups largely unrelated to leukemia subtype (Fig. 2f and Extended Data Fig. 6c), and only the 1° AML-1 sample was distinguished by H3K9me3 (Extended Data Fig. 6d). H3K27me3 is an epigenetically inherited histone modification that is linked to developmental progression as cells determine their identity. Thus, these distinct H3K27me3 leukemia landscapes may be related to hematopoietic transitions that are defective in each tumor.

We next examined the lineage-specific variation in gene and regulatory element usage as indicated by the global chromatin landscape of each of the marks we profiled by performing *t*-SNE of these elements followed by density peak clustering^35^. This analysis revealed that H3K4me1-marked regions were highly variable between lineage subtypes, with a substantial fraction of elements marked specifically in AML samples falling to one side of the *t*-SNE plot (8,221/56,267), ALL-specific elements partitioned to the other side of the plot (8,466/56,267) and CD34^+^ HSPC elements grouped in the middle (7,141/56,267) (Fig. 2g and Extended Data Fig. 6e,f). A fraction of both the AML- and ALL-specific elements were also marked by H3K4me1 in CD34^+^ cells and the primary MPAL samples we profiled (Fig. 2g and Extended Data Fig. 6e,f). This regulatory overlap implies that MPAL leukemias share features with both ALL and AML and that *KMT2A*r leukemia samples maintain H3K4me1 at regulatory elements used during normal hematopoiesis.

While only about half of the H3K4me1-marked elements were similarly labeled across all samples (~50%, 25,973/56,267), a much larger fraction of H3K4me3 (~75%, 10,958/13,998) and H3K36me3 (~85%, 22,858/26,759) peaks were common across leukemia subtypes, indicating that these subtypes largely share gene expression repertoires (Fig. 2h,i and Extended Data Fig. 6g–j). Grouping H3K4me3-marked promoter regions by *t*-SNE also partitioned 64 AML- and 508 ALL-specific elements to opposite sides of the *t*-SNE graph and identified 1,918 elements that were shared with the MPAL samples and CD34^+^ HSPCs (Fig. 2h and Extended Data Fig. 6g,h); however, as compared to H3K4me1, where we identified 23,828 lineage-specific peaks among the 56,267 total peaks (~40%), a smaller proportion of H3K4me3-marked features showed any lineage specificity (2,490/13,998 peaks, or ~18%). This is consistent with previous reports that regulatory elements marked by H3K4me1 generally show more cell type specificity than promoter elements marked by H3K4me3 (refs. ^36,37^).

**Extended Data Fig. 7:**
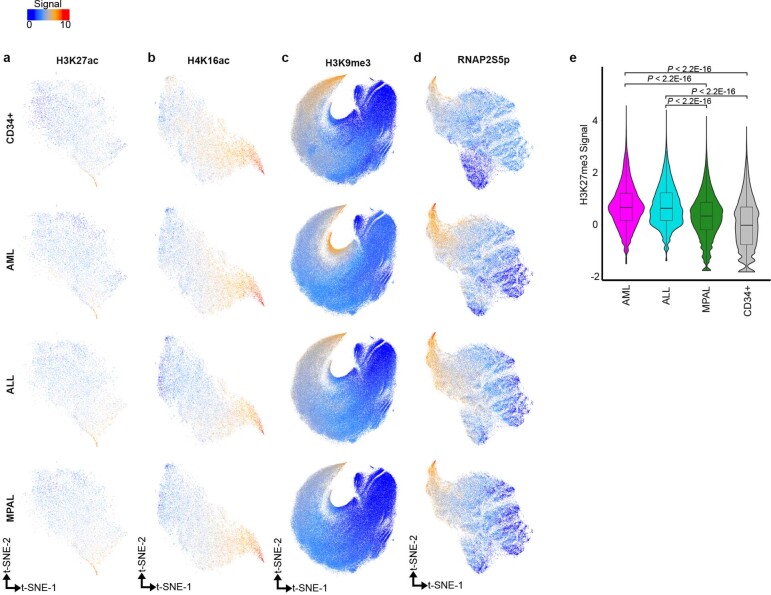
The genome wide distributions of additional active and repressive modifications profiled by AutoCUT&Tag. **a-c**, t-SNE projections of H3K27ac, H4K16ac and H3K9me3 samples do not organize the enriched elements based on lineage specificity. **d**, t-SNE projection of the RNAP2S5p bound regions identifies groups of elements that show CD34 + , AML, ALL and MPAL specific enrichment of RNAP2S5p. **e**, Violin plot comparing the H3K27me3 signal across the composite H3K27me3 peak set identified in all samples. The H3K27me3 signal is significantly lower in the CD34 + and MPAL samples than in the AML and ALL samples, indicating that a greater proportion of the genome is marked by H3K27me3 in the more differentiated AML and ALL samples. Center line = median; box limits = first and third quartiles; whiskers show all data within 1.5 IQRs of the lower and upper quartiles; outliers are not shown; *P* values were computed using a two sample t-test (two-sided); for all samples *n* = 89258 H3K27me3 marked regions.

Similarly to the *t*-SNE analysis of H3K36me3-marked regions, *t*-SNE analysis of H3K27ac-, H4K16ac- and H3K9me3-marked regions did not partition the genome by lineage identity (Extended Data Fig. 7a–c). By contrast, both RNAP2S5p and H3K27me3 peaks showed diversity similar to that observed with H3K4me1 (Fig. 2j and Extended Data Fig. 7d). Analysis by *t*-SNE with H3K27me3 did not partition elements according to lineage subtype (Fig. 2j). Rather, AML and ALL samples had a significantly greater proportion of the genome that was marked by H3K27me3 than CD34^+^ cells (Fig. 2j and Extended Data Fig. 7e), suggesting that these tumor types are more differentiated. In line with this interpretation, MPAL samples had significantly fewer regions marked by H3K27me3 than the ALL or AML samples and were considered to have a higher degree of lineage plasticity (Fig. 2j and Extended Data Fig. 7e). We conclude that high-throughput CUT&Tag profiling provides a powerful tool to characterize *KMT2A*r leukemias and that profiling the developmentally repressed genome reveals tumor-specific differences that are not apparent by profiling the active genome.

### Bivalent chromatin at KMT2A fusion protein target sites

In addition to marking promoters that are engaged in active transcription, H3K4me3 is present at a limited subset of transcriptionally repressed ‘bivalent’ (that is, ‘poised’) promoters that are also marked by H3K27me3 (refs. ^38,39^). In our collection of leukemia samples, we observed both H3K4me3 and H3K27me3 at some promoters that were called as KMT2A fusion protein targets (Fig. 2a, left). Additionally, we observed genes that were bound by the oncofusion protein in the majority of *KMT2A*r leukemia samples but were not called as targets in specific samples; we termed this group ‘missing targets’ (Fig. 2a, right). To systematically define bivalent promoters within our collection of samples, we quantified the abundance of H3K4me3 and H3K27me3 in 2-kb windows centered on gene TSSs of marked and unmarked promoters for each modification. By intersecting these groups, we identified approximately 2,000–5,000 bivalent promoters in each of the *KMT2A*r leukemia samples (Fig. 2k and Supplementary Table 3). Interestingly, we found that approximately 33% (129/396) of promoters for missing targets were called as bivalent, whereas approximately 24% (267/1,097) of KMT2A fusion target promoters were bivalent and only 14% of wild-type KMT2A target promoters were bivalent (Fig. 2l). Thus, oncofusion protein target promoters are enriched for a bivalent chromatin signature, suggesting that expression of these genes may fluctuate among cells within a sample.

### Cell-to-cell chromatin heterogeneity at KMT2A fusion targets

**Fig. 3:**
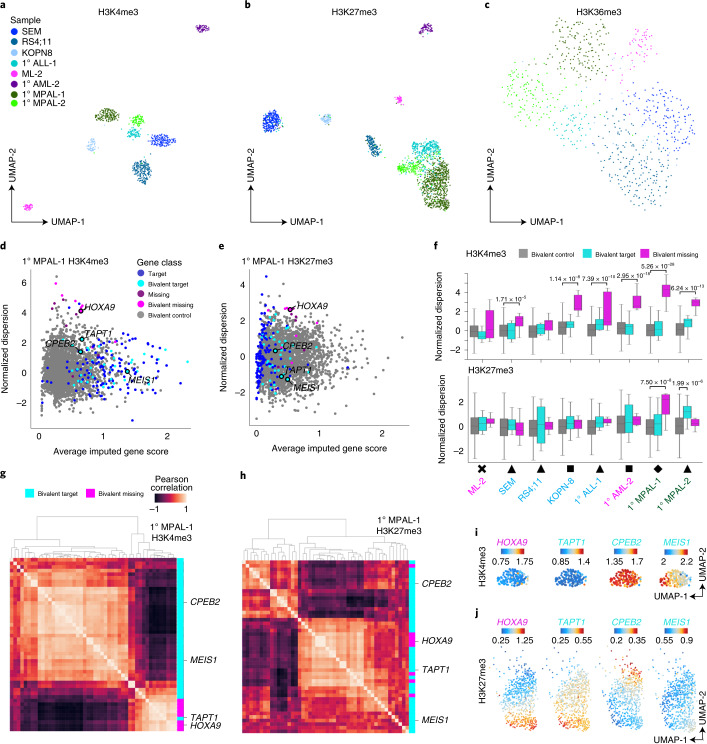
Single-cell profiling of H3K4me3 and H3K27me3 reveals chromatin heterogeneity at KMT2A fusion target loci. **a**, UMAP projection of the H3K4me3 profiles in single leukemia cells resolves sample-specific clusters. **b**, Same as in **a** for H3K27me3. A fraction of cells in the 1° ALL-1, 1° MPAL-1 and 1° MPAL-2 samples intermingle in H3K27me3 UMAP space. **c**, Same as in **a** for H3K36me3. Leukemia cells do not form tight sample-specific groups according to H3K36me3 profile. **d**, Scatterplot comparing the 1° MPAL-1 average imputed H3K4me3 scores and normalized dispersion of genes grouped according to KMT2A fusion-binding status (target, missing target or unbound control) and promoter bivalency status in bulk profiling assays. Select genes are highlighted. **e**, Same as in **d** for H3K27me3. **f**, KMT2A fusion targets show elevated H3K4me3 and H3K27me3 dispersion across select leukemia samples. The center line indicates the median, box limits represent the first and third quartiles, and whiskers show all data within 1.5 times the IQR of the lower and upper quartiles; outliers are not shown. *P* values were computed using a two-sample *t* test (two sided). For each sample, *n* values are listed for bivalent controls, bivalent targets and bivalent missing targets: ML-2: *n* = 2,425, 14, 14; SEM: *n* = 3,441, 8, 15; RS4;11: *n* = 2,635, 6, 9; KOPN-8: *n* = 2,044, 31, 5; 1° ALL-1: *n* = 1,873, 6, 9; 1° AML-2: *n* = 2,483, 17, 12; 1° MPAL-1: *n* = 3,101, 40, 8; 1° MPAL-2: *n* = 3,018, 15, 7. **g**. Organizing genes according to the covariance of H3K4me3 imputed gene scores across 1° MPAL-1 cells resolves groups that vary in concert with one another from cell to cell but are anticorrelated with genes in the other group. Selected genes are highlighted for comparison. **h**, Same as in **g** for H3K27me3. **i**, Imputed H3K4me3 gene scores of the highlighted genes from **g** and **h** displayed on a UMAP plot of 1° MPAL-1 cells shown as a dark green cluster in **a**. **j**, Same as in **i** for H3K27me3. *HOXA9* and *TAPT1* are representative of one group of covariant KMT2A fusion target genes, which are divergent from the second group (for example, *CPEB2* and *MEIS1*).

**Extended Data Fig. 8:**
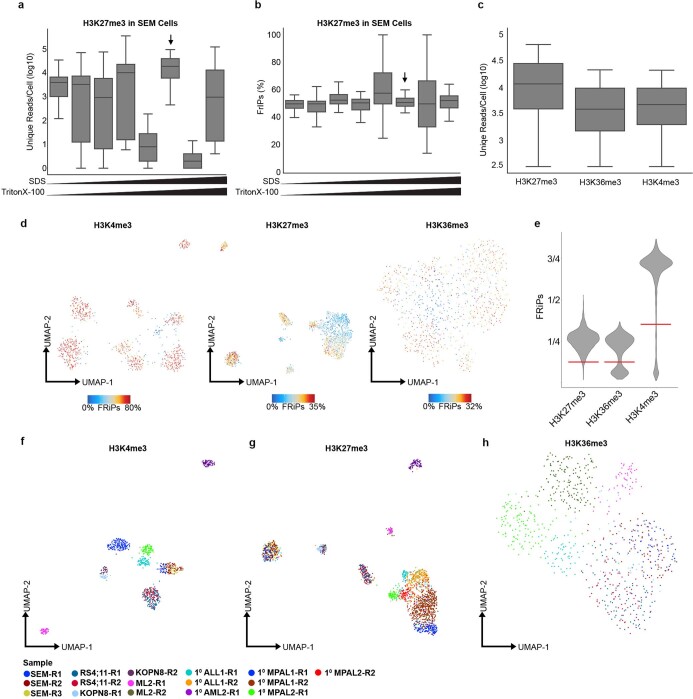
Optimization of CUT&Tag-Direct for single cell applications on the ICELL8. **a**, Titrating the concentration of SDS and Triton-X in the nanowell increases the library yield for individual cells, and identifies optimum conditions for Tn5 release and PCR enrichment (Arrow). For all box plots center line = median; box limits = first and third quartiles; whiskers show all data within 1.5 IQRs of the lower and upper quartiles; outliers are not shown; *P* values were computed using a two sample t-test (two-sided); from left to right *n* = 84, 63, 51, 71, 37, 62, 31, 39 cells. **b**, The Fraction of Reads in Peaks (FRiPs) varies across SDS and Triton-X titration conditions. Arrow indicates the optimum conditions. *N* values the same as in (a). **c**, Boxplot of unique reads per cell across all of the cells profiled for H3K27me3, H3K36me3 and H3K4me3; H3K27me3, *n* = 3,611 cells; H3K36me3, *n* = 1,137 cells; H3K4me3, *n* = 1,528 cells. **d**, UMAP projection of single-cells profiled with H3K4me3, H3K27me3 or H3K36me3 colored according to the FRiP scores of each individual cell using peaks called on the aggregate data of all cells profiled for a given mark. Cells with low FRiP scores tend to fall in between clusters and were removed as a quality control. **e**, Violin plot of FRiP scores for all individual cells profiled using the indicated histone mark; red lines indicate quality control cut-offs for each mark. *N* values same as (c). **f**, UMAP projection of single cells profiled for H3K4me3 and colored according to batch (R1, R2, R3). Replicates profiled on different days group together in UMAP space indicating that batch effects have a minimal impact on clustering cells according to the H3K4me3 profiles. **g**, Same as (f) for H3K27me3. **h**, Same as (f) for H3K36me3.

To test whether the bivalent chromatin signature at KMT2A fusion target promoters is due to heterogeneity among cells, we performed single-cell CUT&Tag on four *KMT2A*r cell lines and four primary *KMT2A*r leukemia samples. Antibody binding and pA-Tn*5* tethering were performed on bulk samples, and individual cells were then arrayed in microwells on the ICELL8 platform for barcoded PCR library enrichment^30^. We optimized the median number of unique reads per cell while maintaining a high fraction of reads in peaks (FRiP) on the ICELL8 by varying the amount of SDS detergent used to release Tn*5* after tagmentation and the amount of Triton X-100 used to quench SDS before PCR (Extended Data Fig. 8a,b). Using this approach, we profiled 1,137–3,611 cells for the H3K4me3, H3K27me3 and H3K36me3 histone modifications. After excluding cells with fewer than 300 fragments, single-cell CUT&Tag for H3K4me3, H3K27me3 and H3K36me3 yielded a median of 4,972, 13,025 and 3,962 unique reads per cell, respectively (Extended Data Fig. 8c). As a second quality-control step, we called peaks on the aggregate data of all cells profiled for each mark and removed cells that had a FRiP value below the normal distribution (Extended Data Fig. 8d,e). Profiles for each single cell were then split into 5-kb bins tiled across the genome, and cells were projected in UMAP space on the basis of this binning (Fig. 3a–c). Encouragingly, cells taken from the same leukemia sample and profiled in different experiments were clustered together in UMAP space, indicating that the data quality was consistent between batches (Extended Data Fig. 8f–h). This approach resolved clusters for samples based on H3K4me3 or H3K27me3 profiling but not H3K36me3 profiling (Fig. 3a–c). This implies that the leukemia samples differ in both sets of active promoters and silenced regions.

To examine intratumoral heterogeneity in the H3K4me3 and H3K27me3 signals, we first used the archR single-cell software package^40,41^ to calculate imputed gene scores for all genes according to the UMAP projection of all cells. We then determined the normalized dispersion of the imputed scores in cells from the same sample (Fig. 3d,e). Strikingly, bivalent missing targets showed significantly higher H3K4me3 dispersion in the SEM, KOPN-8, 1° ALL-1, 1° AML-2, 1° MPAL-1 and 1° MPAL-2 samples than tumor-matched control genes (Fig. 3f). This implies that levels of the H3K4me3 active promoter mark in these genes vary among cells within *KMT2A*r leukemias.

Next, we examined variation in the repressive H3K27me3 mark at bivalent oncoprotein target genes. In 1° MPAL-1 cells, the normalized dispersion of H3K27me3 was significantly higher in bivalent missing target genes, and in the 1° MPAL-2 sample the normalized H3K27me3 dispersion was higher in bivalent target genes than in tumor-matched control genes (Fig. 3f). Some bivalent genes varied among cells for both the H3K4me3 and H3K27me3 modifications. For example, the *HOXA9* gene was a missing target in 1° MPAL-1 cells (Fig. 2a) but showed high dispersion in both the H3K4me3 and H3K27me3 signals (Fig. 3d,e). Thus, bivalency of chromatin marks is associated with heterogeneity among cells within a sample.

**Extended Data Fig. 9:**
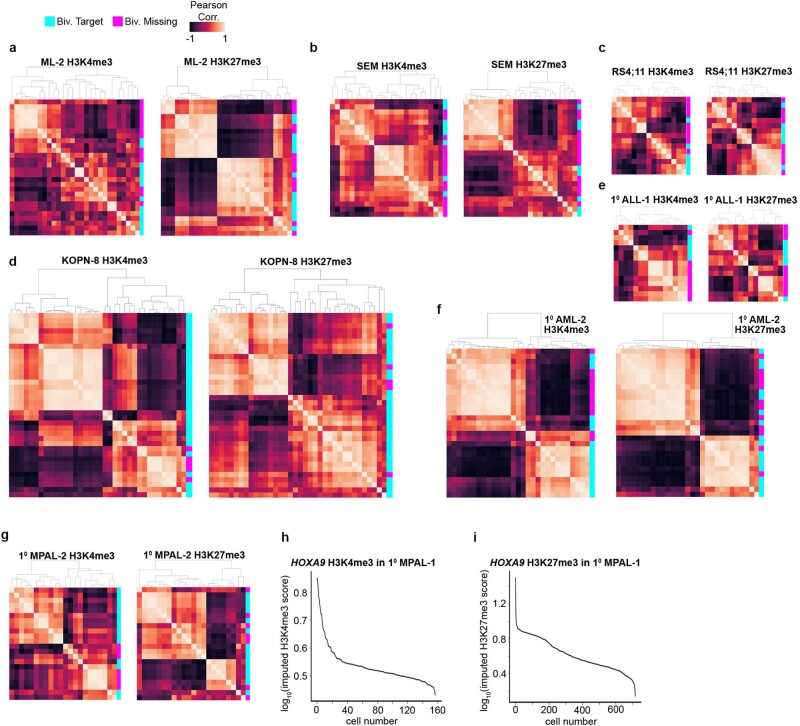
Networks of KMT2A fusion target genes show divergent patterns of active and repressive chromatin marks within the same leukemia. **a**, Organizing genes according to the co-variance of either the imputed H3K4me3 (left) or H3K27me3 (right) scores across ML-2 cells resolves groups that vary in concert with one another from cell-to-cell but are anti-correlated with genes in the other group. Bivalent targets (Cyan) and bivalent missing targets (Magenta) are indicated. **b**, Same as (a) for SEM cells. **c**, Same as (a) for RS4;11 cells. **d**, Same as (a) for KOPN-8 cells. **e**, Same as (a) for the 1^0^ ALL-1 cells. **f**, Same as (a) for the 1^0^ AML-2 cells. **g**, Same as (a) for the 1^0^ MPAL-2 cells. The ML-2, KOPN-8, 1^0^ AML-2 and 1^0^ MPAL-2 cells show the clearest distinction between divergent groups of oncoprotein target genes. **h**, Knee plot showing the distribution of the imputed *HOXA9* H3K4me3 scores across all cells profiled from the 1^0^ MPAL-1 sample. Only ~15% of cells have a log-transformed *HOXA9* H3K4me3 score > 0.6. **i**, Same as (h) but showing imputed *HOXA9* H3K27me3 scores. The majority of 1^0^ MPAL-1 cells (~55%) have a log-transformed *HOXA9* H3K27me3 score > 0.6.

Grouping bivalent target genes according to the Pearson correlation of their imputed gene scores across cells of a given leukemia sample separated two groups by either H3K4me3 or H3K27me3 profiling (Fig. 3g,h and Extended Data Fig. 9a–g). For example, the missing target gene *HOXA9* had elevated H3K4me3 scores in a small fraction of 1° MPAL-1 leukemia cells (~15%) (Fig. 3i and Extended Data Fig. 9h). The *TAPT1* gene clustered together with *HOXA9* (Fig. 3g) and, as expected, had the highest H3K4me3 scores in the same cells as *HOXA9* (*r* = 0.87) (Fig. 3i). By contrast, genes that were anticorrelated with *HOXA9*, such as *CPEB2* (*r* = −0.74) and *MEIS1* (*r* = −0.42), had the weakest H3K4me3 signal in cells where *HOXA9* was active (Fig. 3i). This suggests that there are two exclusive gene expression programs activated by KMT2A fusion oncoproteins. Furthermore, we found that the imputed H3K27me3 scores also formed inverse patterns of gene association from H3K4me3, where genes such as *HOXA9* that were rarely marked by H3K4me3 in 1° MPAL-1 leukemia cells showed elevated H3K27me3 scores in the majority of tumor cells (~55%) (Fig. 3j and Extended Data Fig. 9i). These groups of divergent KMT2A fusion oncoprotein targets may contribute to the phenotypic plasticity of *KMT2A*r leukemias.

### AutoCUT&Tag profiling predicts drug sensitivity of leukemias

**Fig. 4:**
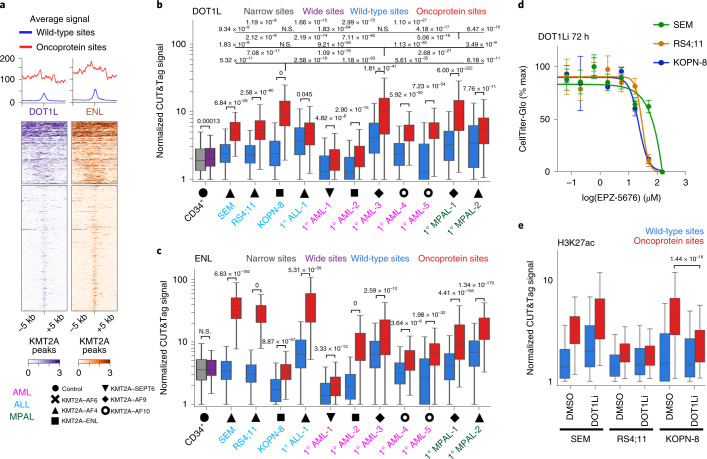
AutoCUT&Tag profiling reveals therapeutic sensitivity of *KMT2A*r leukemia samples to DOT1L inhibition. **a**, Heatmaps comparing the co-occupancy of the transcriptional cofactors DOT1L and ENL over 10-kb windows centered on fusion oncoprotein sites (top) and sample-matched wild-type sites (bottom) in the 1° MPAL-1 sample. **b**, DOT1L is significantly enriched at fusion oncoprotein-binding sites in all *KMT2A*r leukemia samples. The KMT2A–AF9-bearing 1° AML-3 and 1° MPAL-1 samples as well as the KMT2A–ENL-bearing cell line KOPN-8 show the strongest enrichment of DOT1L at fusion oncoprotein sites. For all box plots, the center line indicates the median, box limits represent the first and third quartiles, and whiskers show all data within 1.5 times the IQR of the lower and upper quartiles; outliers are not shown. *P* values were computed using a two-sample *t* test (two sided). *n* values are listed as narrow sites and wide sites for control samples and as wild-type sites and oncoprotein sites for *KMT2A*r samples: CD34^+^: *n* = 6,204, 131; SEM: *n* = 8,168, 91; RS4;11: *n* = 10,287, 92; KOPN-8: *n* = 9,723, 192; 1° ALL-1: *n* = 3,747, 156; 1° AML-1: *n* = 15,915, 349; 1° AML-2: *n* = 26,148, 423; 1° AML-3: *n* = 3,943, 103; 1° AML-4: *n* = 7,218, 270: 1° AML-5: *n* = 11,256, 186; 1° MPAL-1: *n* = 8,641, 248; 1° MPAL-2: *n* = 15,179, 189. **c**, ENL is significantly enriched at fusion oncoprotein-binding sites in all *KMT2A*r leukemia samples. *n* values are the same as in **b**. **d**, Cell survival curves of the SEM, RS4;11 and KOPN-8 cell lines in response to increasing concentrations of the DOT1L inhibitor EPZ-5676 (DOT1Li). Error bars indicate the s.d. of three replicates. **e**, In response to treatment with 30 µM of the DOT1L inhibitor for 3 d, H3K27ac is depleted at fusion oncoprotein-binding sites in KOPN-8 cells but not SEM or RS4;11 cells. *n* values are the same as in **b**.

We reasoned that the distinct binding sites of KMT2A fusion proteins might be driven in part by the cofactors with which the fusion oncoproteins associate. Therefore, we used AutoCUT&Tag to map the distributions of ENL and DOT1L, two chromatin proteins that interact with KMT2A fusion proteins^33^. Regions bound by KMT2A fusion proteins were enriched for DOT1L and ENL in all samples as compared to sample-matched wild-type KMT2A-bound sites (Fig. 4a–c). DOT1L has been proposed to be a central component of oncogenic transformation by KMT2A fusion proteins in certain leukemias^14,42^, and we found that the DOT1L histone methyltransferase was significantly more enriched at oncofusion protein targets in KOPN-8, 1° AML-3 and 1° MPAL-1 samples than in the other leukemias we profiled (Fig. 4b). Both 1° AML-3 and 1° MPAL-1 carry a KMT2A–AF9 fusion protein, whereas KOPN-8 carries a KMT2A–ENL fusion, suggesting that the AF9 fusion partner recruits particularly high levels of DOT1L to oncoprotein target loci while other leukemias can be variable for DOT1L recruitment. This was also illustrated by the KMT2A–SEPT6 fusion (1° AML-1), where only modest enrichment of DOT1L and ENL at fusion-binding sites was observed.

Several studies have suggested that KMT2A–AF9-bearing leukemias are particularly sensitive to pharmacological inhibition of DOT1L methyltransferase activity^43,44^. We hypothesized that elevated DOT1L signal at oncoprotein target sites might be indicative of sensitivity to DOT1L inhibitors. Indeed, we found that KOPN-8 cells were more sensitive to the DOT1L inhibitor EPZ-5676 (half-maximal inhibitory concentration (IC_50_) = 22.45 µM) than either SEM (IC_50_ = 110.45 µM) or RS4;11 (IC_50_ = 29.71 µM) cells (Fig. 4d). Previous reports have also shown that KOPN-8 cells are sensitive to the DOT1L inhibitor EPZ-0007477 (ref. ^44^). We suspect that the EPZ-5676 IC_50_ values obtained here are higher than those previously reported for *KMT2A*r cell lines because we only exposed cells to the inhibitor for 72 h rather than 4–10 d^45,46^. In line with the increased sensitivity of KOPN-8 cells to DOT1L inhibitors, after 72 h of EPZ-5676 treatment, we found a significant depletion of the active histone mark H3K27ac at oncofusion protein-binding sites in KOPN-8 cells but not at KMT2A–AF4-bound sites in the SEM or RS4;11 leukemia cell lines (Fig. 4e). Thus, this pharmacological agent specifically alters the chromatin of oncofusion protein targets in *KMT2A*r leukemia samples.

**Fig. 5:**
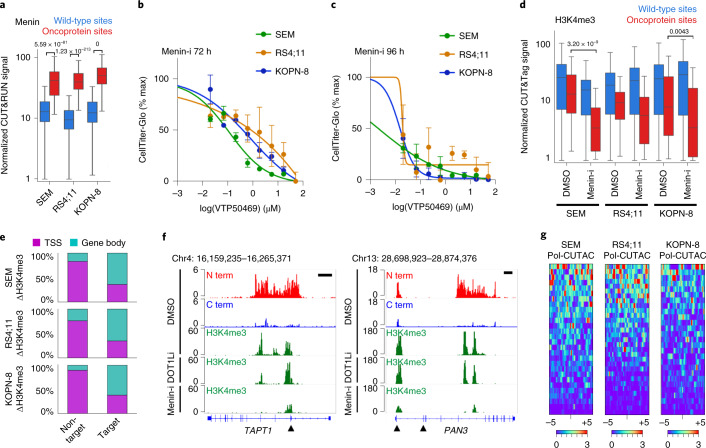
Aberrant enrichment of H3K4me3 in KMT2A fusion target gene bodies is sensitive to disruption of Menin localization. **a**, The transcriptional scaffold protein Menin is enriched at fusion oncoprotein-binding sites in *KMT2A*r cell lines. For all box plots, the center line indicates the median, box limits represent the first and third quartiles, and whiskers show all data within 1.5 times the IQR of the lower and upper quartiles; outliers are not shown. *P* values were computed using a two-sample *t* test (two sided). For each sample, *n* values are listed for wild-type sites and oncoprotein sites: SEM: *n* = 8,168, 91; RS4;11: *n* = 10,287, 92; KOPN-8: *n* = 9,723, 192. **b**, Cell survival curves for the SEM, RS4;11 and KOPN-8 cell lines in response to treatment with increasing concentrations of the Menin binding inhibitor VTP50469 (Menin-i) for 72 h. Error bars indicate the s.d. of three replicates. **c**, Same as in **b** except that treatment with VTP50469 was extended to 96 h. **d**, In response to treatment with 30 µM VTP50469 for 3 d, H3K4me3 is depleted at fusion oncoprotein-binding sites. *n* values are the same as in **a**. **e**, H3K4me3 peaks that show a significant loss of signal in response to treatment with VTP50469 are enriched in the gene bodies of oncoprotein target genes. **f**, Two examples of KMT2A–AF4-bound genes (*TAPT1* and *PAN3*) in SEM cells that lose H3K4me3 in the gene body in response to treatment with 30 µM of the Menin inhibitor for 3 d. Black arrowheads point to annotated TSSs. Black scale bars, 10 kb. **g**, Heatmaps showing that Menin-sensitive H3K4me3 peaks located in the fusion oncoprotein target gene bodies of the indicated cell types are accessible and enriched for initiating RNA polymerase II, as indicated by RNAP2S5p CUTAC (Pol-CUTAC). Heatmaps are centered on Menin-sensitive H3K4me3 peaks falling in oncoprotein target gene bodies.

**Extended Data Fig. 10:**
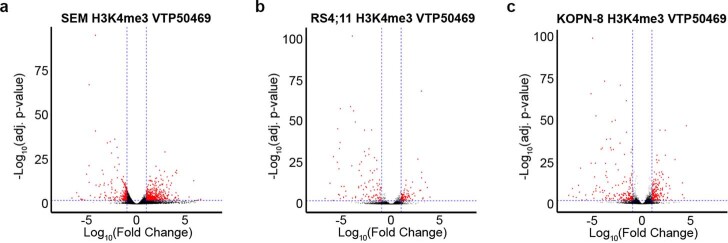
Identification of H3K4me3-marked regions that are sensitive to disruption of Menin localization by VTP50469. **a**, Volcano plot showing the H3K4me3 peaks in SEM cells that are significantly altered (red dots) by exposure to 30 µM VTP50469 for 72 hrs. The dotted lines indicate that peaks with greater than a twofold change in H3K4me3 signal (x-axis) and an adjusted p-value < 0.01 (y-axis) were called as significantly changed by exposure to 30 µM VTP50469. **b**, Same as (a) for RS4;11 cells. **c**, Same as (a) for KOPN-8 cells. All comparisons were done using two replicates of H3K4me3 profiles from DMSO and VTP50469 treated cells.

To extend this analysis, we profiled the transcriptional scaffold protein Menin, which interacts with the N-terminal portion of KMT2A and with oncofusion proteins, by using AutoCUT&RUN. SEM, RS4;11 and KOPN-8 cells had similar levels of Menin at KMT2A fusion-bound sites, but the SEM cell line was more sensitive to the Menin inhibitor VTP50469 (IC_50_ = 2.4 nM) than RS4;11 (IC_50_ = 18.7 nM) or KOPN-8 (IC_50_ = 14.7 nM) cells (Fig. 5a–c). We then used AutoCUT&Tag to profile H3K4me3 in VTP50469-treated cells (Fig. 5d). We called H3K4me3 peaks that showed a significantly depleted signal after drug treatment (Extended Data Fig. 10), and these depleted sites were highly enriched in the gene bodies of oncofusion protein targets (Fig. 5e,f). Finally, we examined chromatin accessibility and the presence of initiating RNA polymerase II (RNAP2S5p) in drug-treated cells using Pol-CUTAC^47,48^ and found that many oncofusion- and Menin-bound sites were normally highly accessible and bound by initiating RNA polymerase II (Fig. 5g). This supports the idea that oncofusion protein-induced transcription in *KMT2A*r leukemias is highly sensitive to Menin inhibition.

## Discussion

Here we have applied high-throughput chromatin profiling to *KMT2A*r leukemias to delineate fusion protein-specific targets and to identify chromatin features that are characteristic of myeloid, lymphoid and mixed-lineage leukemias. To economically profile these features, we took advantage of the high signal-to-noise ratio and low sequencing depth requirements inherent to CUT&RUN and CUT&Tag and fully automated both methods on a standard liquid handling robot. As CUT&Tag requires only thousands of cells for informative histone modifications^47^, AutoCUT&Tag is suitable for profiling of samples for a wide range of studies, including developmental and disease studies, and screening of patient samples. The enhanced throughput and consistency of the AutoCUT&RUN and AutoCUT&Tag platforms for chromatin profiling make these technologies suitable for profiling patient specimens.

By also performing AutoCUT&RUN on KMT2A fusions and components of the Super Elongation and DotCom complexes, we have elucidated the details of mechanisms that likely contribute to the heterogeneity of these tumors. We found that the most common KMT2A fusion proteins, including KMT2A–AF4, KMT2A–AF9, KMT2A–ENL and KMT2A–AF10, all colocalize with the DOT1L and ENL proteins in gene bodies. This suggests that interaction of the C-terminal domains of AF4, AF9, ENL and AF10 with transcriptional elongation complexes likely recruits fusion proteins from the promoter into the gene body. In line with the possibility that these interactions have a pivotal role in oncogenic transformation, wild-type ENL protein is required for tumor growth in numerous *KMT2A*r cell lines^49^.

Using AutoCUT&Tag to profile histone modifications in leukemia samples, we identified frequent KMT2A fusion oncoprotein sites with bivalent chromatin features. At some sites, bivalent chromatin features correlated with heterogeneity among cells of the same tumor, which suggests that the heterogeneity in gene expression seen in populations of mixed-lineage leukemia cells is rooted in chromatin dynamics. We identify a group of KMT2A oncoprotein target genes that are shared in the majority of *KMT2A*r leukemias but are missed in a subset of samples. In several of the *KMT2A*r leukemia samples we profiled, these missing targets were among the genes that showed the highest variation in active and repressive chromatin marks within the tumor, suggesting that these missing targets may be bound and activated by the oncoprotein in a limited subset of cells within the tumor, causing them to fall below the levels necessary for detection in our bulk KMT2A profiling assays. This heterogeneity we observed at KMT2A oncoprotein target genes has implications for how resistance to therapies may develop, if only a subset of cells are susceptible to specific anticancer agents.

Heterogeneity in leukemias may arise if an early cancerous cell divides and differentiates into two related cell types. Alternatively, certain leukemias may sporadically switch between cell types^21,50^. Our single-cell profiling reveals that some leukemias display both active and repressive chromatin states at KMT2A fusion target loci that differ among individual cells. Kinetic analysis of chromatin dynamics within cell populations will be needed to determine whether bivalency reflects differentiation or sporadic switching, with implications for therapeutic strategies to limit relapse.

Multiple compounds targeting chromatin proteins have shown promise as therapeutics for certain leukemias^44,51^. Profiling the targets of these compounds distinguishes certain *KMT2A*r leukemias in which DOT1L is enriched at KMT2A fusion oncoprotein target sites, thus providing a strategy for selecting patients for whom treatment with DOT1L-targeting compounds is suitable. We also identified samples where KMT2A fusion oncoprotein target genes are broadly enriched for H3K4me3 in gene bodies and also bound by initiating RNA polymerase II. These leukemias are particularly sensitive to treatment with the Menin inhibitor VTP50469 and again demonstrate the utility of chromatin profiling for selecting therapeutic treatments. Incorporating AutoCUT&RUN and AutoCUT&Tag into longitudinal clinical trials could thus provide a route to assess the efficacy of epigenetic medicines. In addition, these technologies are highly consistent between replicates and increase the number of samples that can be processed and sequenced in parallel by an order of magnitude relative to conventional chromatin profiling, suggesting that it could be feasible to apply AutoCUT&RUN and AutoCUT&Tag for patient diagnosis.

## Methods

### Patients

All patient samples were obtained by St. Jude Children’s Research Hospital or member COG institutions in accordance with the Declaration of Helsinki after written consent from the parents/guardians of minors upon enrolling in the trial. The studies were overseen by the institutional review boards at Fred Hutchinson Cancer Research Center (IR protocol 9950) and St. Jude Children’s Research Hospital. Patients did not receive compensation for participation in this study.

### Cell culture

Human K562 cells were purchased from ATCC (CCL-243) and cultured according to the supplier’s protocol. H1 human embryonic stem cells were obtained from WiCell (WA01-lot# WB35186) and cultured in plates coated with Matrigel (Corning) in mTeSR1 Basal Media (STEMCELL Technologies, 85851) containing mTeSR1 Supplement (STEMCELL Technologies, 85852). The *KMT2A*r cell lines ML-2, KOPN-8, RS4;11 and SEM were obtained from the Bleakley laboratory at the Fred Hutchinson Cancer Research Center. The SEM cell line was cultured in IMDM (ThermoFisher, 12440061) supplemented with 10% FBS. The ML-2, KOPN-8 and RS4;11 cell lines were cultured in RPMI 1640 with glutamine and HEPES (ThermoFisher, 72400047) supplemented with 10% FBS. All cell lines were maintained in a cell culture incubator (Sanyo, MCO-19AIC) with standard settings (37 °C with 5% CO_2_).

### Drug treatment

Ten thousand SEM, RS4;11 and KOPN-8 cells were plated in 90 µl of the appropriate medium (see above) in a 96-well cell culture plate. Serial dilutions of either the DOT1L inhibitor EPZ-5676 (MedChem Express, HY-15593) or the Menin inhibitor VTP50469 (MedChem Express, HY-114162) were prepared in DMSO and then diluted in primary medium to control for the concentration of DMSO across all conditions. Ten microliters of the diluted inhibitors was then added to cell culture suspensions followed by mixing. Cells were grown for 3 or 4 d, at which point viability was measured using a CellTiter-Glo assay (Promega, G9241) read out on a standard luminometer. For chromatin profiling experiments, SEM, RS4;11 and KOPN-8 cells were plated at the same density (10,000 cells per 100 µl) in 20 ml of medium containing 30 µM EPZ-5676, 30 µM VTP50469 or DMSO alone. After 3 d in culture, the cells were harvested and prepared for either AutoCUT&RUN or AutoCUT&Tag processing.

### Primary patient samples

Diagnoses of acute leukemia were made by hematopathologists at the respective institutions based on review of histological, cytogenetic, flow cytometry and molecular studies of bone marrow biopsy samples and aspirates in accordance with World Health Organization guidelines^52^. Whole blood from patients with bone marrow blast percentages above 88% was subjected to Ficoll centrifugation to remove red blood cells and neutrophils. Ten million mononuclear cells were resuspended in FBS with 10% DMSO and slowly frozen in a Mr. Frosty isopropanol cannister for 24 h before being transferred to a liquid nitrogen tank. Cryopreserved leukemia blasts for 1° MPAL-1 (sample ID: SJMPAL012424_D1, alias TB-11-3295) and 1° ALL-1 (sample ID: SJALL048347_D1, alias TB-13-0939) were obtained from St. Jude Children’s Research Hospital in accordance with institutional regulatory practices. Cryopreserved leukemia blasts for 1° AML-1 (sample ID: A40725), 1° AML-2 (sample ID: A67194), 1° AML-3 (sample ID: A107909), 1° AML-4 (sample ID: A38481), 1° AML-5 (sample ID: A109016) and 1° MPAL-2 (sample ID: A58548) were obtained from the Meshinchi laboratory at the Fred Hutchinson Cancer Research Center. The KMT2A fusion present in each sample was determined by whole-genome and targeted capture sequencing as previously described^53,54^. Cryopreserved CD34^+^ HSPCs from a single granulocyte colony-stimulating factor (G-CSF)-mobilized donor, enriched using a Miltenyi CliniMacs device without expansion in culture, were obtained from the Fred Hutchinson Cooperative Centers of Excellence in Hematology Core in accordance with institutional regulatory practices.

### Antibodies

For profiling wild-type and oncogenic KMT2A proteins, we used two antibodies targeting the KMT2A N terminus (mouse monoclonal anti-KMT2A (1:100; Millipore, clone N4.4, 05-764) referred to as KMT2A-N1 and rabbit monoclonal anti-KMT2A (1:100; Cell Signaling Technology, clone D2M7U, 14689) referred to as KMT2A-N2) as well as two antibodies targeting the KMT2A C terminus (mouse monoclonal anti-KMT2A (1:100; Millipore, clone 9-12, 05-765) referred to as KMT2A-C1 and mouse monoclonal anti-KMT2A (1:100; Santa Cruz, clone H-10, sc-374392) referred to as KMT2A-C2). Because pA-MNase does not bind efficiently to many mouse antibodies, we used rabbit anti-mouse IgG (1:100; Abcam, ab46540) as an adaptor; this antibody was also used in the absence of a primary antibody as an IgG negative control. For profiling Menin via AutoCUT&RUN, we used rabbit polyclonal anti-Menin (1:50; Bethyl, A300-105A). For profiling Super Elongation and DotCom components via manual and automated CUT&Tag, we used rabbit monoclonal anti-ENL (1:50; Cell Signaling Technology, clone D9M4B, 14893) and rabbit monoclonal anti-DOT1L (1:50; Cell Signaling Technology, clone D4O2T, 90878). For profiling histone marks via manual and automated CUT&Tag, as well as single-cell CUT&Tag, we used rabbit oligoclonal anti-H3K4me1 (1:100; Thermo, 710795), rabbit polyclonal anti-H3K4me3 (1:100 for bulk profiling or 1:10 for single-cell experiments; Active Motif, 39159), rabbit polyclonal anti-H3K36me3 (1:100 for bulk profiling or 1:10 for single-cell experiments; Epicypher, 13-0031), rabbit monoclonal anti-H3K27me3 (1:100 for bulk profiling or 1:10 for single-cell experiments; Cell Signaling Technology, clone C36B11, 9733), rabbit polyclonal anti-H3K9me3 (1:100; Abcam, ab8898), rabbit monoclonal anti-H3K27ac (1:50; Millipore, clone RM172, MABE647), rabbit monoclonal anti-H4K16ac (1:50; Abcam, ab109463) and rabbit monoclonal anti-RNAP2S5p (1:100; Cell Signaling Technology, clone D9N5I, 13523). To increase the local concentration of pA-Tn*5*, all CUT&Tag reactions also included the secondary antibody guinea pig anti-rabbit IgG (1:100; antibodies-online, ABIN101961).

### AutoCUT&RUN

Primary patient samples were thawed at room temperature, washed and bound to concanavalin-A (ConA) paramagnetic beads (Bangs Laboratories, BP531) for magnetic separation. Samples were then suspended in antibody binding buffer and split for incubation with antibodies specific to the KMT2A N or C terminus or IgG control antibody overnight. Sample processing was performed by the CUT&RUN core facility at the Fred Hutchinson Cancer Research Center according to the AutoCUT&RUN protocol available from the protocols.io website (10.17504/protocols.io.ufeetje).

### CUT&Tag

Manual CUT&Tag reactions were performed according to the CUT&Tag-direct protocol^31^. Briefly, nuclei were prepared by suspending cells in NE1 buffer (20 mM HEPES-KOH pH 7.9, 10 mM KCl, 0.5 mM spermidine, 0.1% Triton X-100, 20% glycerol) for 10 min on ice. Samples were then spun down and resuspended in wash buffer (20 mM HEPES pH 7.5, 150 mM NaCl, 0.5 mM spermidine, Roche Complete Protease Inhibitor EDTA-Free) and lightly cross-linked by addition of 16% formaldehyde to a final concentration of 0.1%. After 2 min, cross-linking was stopped by addition of 2.5 M glycine to a final concentration of 75 mM. Nuclei were washed and either cryopreserved in a Mr. Frosty chamber for long-term storage or bound to ConA magnetic beads for further processing. ConA-bound nuclei were suspended in antibody binding buffer (wash buffer containing 2 mM EDTA) and split into individual 0.5-ml tubes for incubation with antibody at room temperature for 1 h or 4 °C overnight. Samples were then washed to remove unbound primary antibody, resuspended in wash buffer containing the secondary antibody and incubated at 4 °C for 1 h. Samples were washed and resuspended in 300-wash buffer (wash buffer with 300 mM NaCl) containing pA-Tn*5* (1:150 dilution) and incubated at 4 °C for 1 h. Samples were then washed in 300-wash buffer and resuspended in tagmentation buffer (300-wash buffer plus 10 mM MgCl_2_) and incubated at 37 °C for 1 h to allow the Tn*5* tagmentation reaction to go to completion. Samples were washed with TAPS wash buffer (10 mM TAPS with 0.2 mM EDTA) and resuspended in 5 µl of release solution (10 mM TAPS with 0.1% SDS). Samples were then incubated in a thermocycler with a heated lid at 58 °C for 1 h to release Tn*5* and prepare tagmented chromatin for PCR. Neutralizing solution (15 µl of 0.67% Triton X-100) was added followed by 2 µl of barcoded i5 primer (10 µM), 2 µl of barcoded i7 primer (10 µM) and 25 µl of NEBNext PCR mix. Samples were then placed in a thermocycler and PCR amplification was performed using 12–14 rapid cycles. CUT&Tag libraries were cleaned with a single round of SPRIselect beads (Beckman Coulter, B23319) at a 1.3 to 1 (vol/vol) ratio of beads to sample, quantified on a TapeStation Bioanalyzer instrument and pooled for sequencing.

### AutoCUT&Tag

A detailed protocol complete with program downloads has been made publicly available on protocols.io for implementing AutoCUT&Tag on a Beckman Coulter Biomek liquid handling robot (10.17504/protocols.io.bgztjx6n). To facilitate adaptation of the method to other standard liquid handling modules, the complete specifications for each step in the automated procedure are outlined in the guidelines section. Briefly, nuclei were extracted, lightly cross-linked, bound to ConA beads and incubated with primary antibody as in manual CUT&Tag. Up to 96 samples were then arrayed in a 96-well PCR plate and positioned on a stationary ALP on the Beckman Coulter Biomek FX robot equipped with an ALPAQUA Magnet Plate for standard magnetic separation, an ALPAQUA LE Magnet Plate for low-volume elution and a thermal block for temperature-controlled incubation. Wash buffer and 300-wash buffer were loaded in deep-well plates and secondary antibody solution, pA-Tn*5* solution, tagmentation buffer, TAPS buffer and release buffer were all loaded into V-bottom plates and were positioned on stationary ALPs in accordance with the preprogrammed AutoCUT&Tag method. AutoCUT&Tag processing was conducted over the course of 4 h. The sample plate containing ConA-bound tagmented nuclei in 10 µl of 0.1% SDS was then removed, sealed and placed on a thermocycler with a heated lid for a 1-h incubation at 58 °C. Using a reservoir and multichannel pipettor, 54 µl of 0.15% SDS neutralization solution was added to each well, followed by 4 µl of premixed i5 and i7 barcoded primers and 36 µl of premixed KAPA PCR Master Mix. The plate was then sealed and returned to a thermocycler for 14 rapid PCR cycles. Following PCR amplification, the sample plate was returned to the Biomek for one round of post-PCR cleanup on the Biomek deck setup in accordance with a preprogrammed post-PCR cleanup method, including a second 96-well plate preloaded with SPRIselect beads, a deep-well plate loaded with 80% ethanol for bead washes and two V-bottom plates preloaded with 10 mM Tris-HCl pH 8.0 for tip washes and elution. Upon completion of the 1-h cleanup, the samples were quantified using a TapeStation Bioanalyzer instrument and pooled for sequencing.

### Single-cell CUT&Tag

Nuclei were extracted and lightly cross-linked using the same strategy as for manual CUT&Tag. The nuclei concentration was then quantified using a Vi-CELL analyzer (Beckman Coulter) to allow for accurate dilution to 400 nuclei per µl (see below) before dispensing into nanowells on the ICELL8. For each antibody, 10 µl of ConA beads were washed in binding buffer (20 mM HEPES-KOH pH 7.9, 10 mM KCl, 1 mM CaCl_2_, 1 mM MnCl_2_) and bound to the sample for 10 min. Samples were then split into 0.5-ml Lobind tubes, one for each antibody, and resuspended in 25 µl of antibody buffer containing primary antibody at a 1:10 dilution. Samples were incubated at 4 °C overnight, washed twice with 100 µl of wash buffer and then resuspended in 50 µl of wash buffer containing secondary antibody at a 1:50 dilution. Samples were incubated at 4 °C for 1 h, washed twice with 100 µl of wash buffer and then resuspended in 50 µl of 300-wash buffer with a 1:50 dilution of pA-Tn*5*. Samples were incubated at 4 °C for 1 h, washed twice with 100 µl of 300-wash buffer and then resuspended in 50 µl of tagmentation solution (300-wash buffer with 10 mM MgCl_2_). Samples were incubated at 37 °C in a thermocycler with a heated lid for 1 h to allow the tagmentation reaction to go to completion. Samples were then washed with 10 mM TAPS to remove any residual salt and resuspended in 10 mM TAPS pH 8.5 containing 1× DAPI and 1× secondary diluent reagent (Takara, 640196) at a concentration of 400 nuclei per µl. Eighty microliters of cell suspension was loaded into 8 wells of a 384-well plate, together with 25 µl of fiducial reagent (Takara, 640196), according to the manufacturer’s instructions. Sample suspension (35 nl) was dispensed on the ICELL8 into the nanowells of a 350v Chip (Takara, 640019). The 350v chip was dried and sealed, and cells were centrifuged at 1,200*g* for 3 min. The chip was then imaged to identify wells containing a single nucleus, and a filter file was prepared. During image processing, 35 nl of 0.19% SDS in TAPS was added to all nanowells on the ICELL8 using an unfiltered dispense. The chip was then dried, sealed and centrifuged at 1,200*g* for 3 min and heated at 58 °C in a thermocycler with a heated lid for 1 h to release pA-Tn*5* and prepare the tagmented chromatin for PCR. Before opening, the chip was centrifuged at 1,200*g* and 35 nl of 2.5% Triton X-100 neutralization solution was added to all wells containing a single nucleus via a filtered dispense on the ICELL8. The chip was then dried and 35 nl of i5 indices was added via a filtered dispense. The chip was dried and 35 nl of i7 indices was added via a filtered dispense. The chip was dried, sealed and centrifuged at 1,200*g* for 3 min. Then, 100 nl of KAPA PCR mix (2.775× HiFi buffer, 0.85 mM dNTPs, 0.05 U KAPA HiFi polymerase per µl) (Roche, 07958846001) was added to all wells containing a single nucleus via two 50-nl filtered dispenses. The chip was centrifuged at 1,200*g* for 3 min, sealed and placed in a thermocycler for PCR amplification using the following conditions: 1 cycle at 58 °C for 5 min; 1 cycle at 72 °C for 10 min; 1 cycle at 98 °C for 45 s; 15 cycles at 98 °C for 15 s, 60 °C for 15 s and 72 °C for 10 s; and 1 cycle at 72 °C for 2 min. The chip was then centrifuged at 1,200*g* for 3 min into a collection tube (Takara, 640048). To remove residual PCR primers and detergent, the sample was cleaned using two rounds of SPRIselect bead cleanup at a 1.3 to 1 (vol/vol) ratio of beads to sample. Samples were resuspended in 30 µl of 10 mM Tris-HCl pH 8.0, quantified on a TapeStation Bioanalyzer instrument and pooled with bulk samples for sequencing.

### DNA sequencing and data processing

The size distribution and molar concentration of libraries were determined using an Agilent 4200 TapeStation. Up to 48 barcoded CUT&RUN libraries or 96 barcoded CUT&Tag libraries were pooled at approximately equimolar concentration for sequencing. Paired-end 2 × 25 bp sequencing on the Illumina HiSeq 2500 platform was performed by the Fred Hutchinson Cancer Research Center Genomics Shared Resources. This yielded 5–10 million reads per antibody. Single-cell CUT&Tag libraries were prepared using unique i5 and i7 barcodes and pooled with bulk samples for sequencing. For 500–1,000 cells, 20 million reads was sufficient to obtain an average of approximately 80% saturation of the estimated library size for each single cell. Paired-end reads were aligned using Bowtie2 version 2.3.4.3 to UCSC hg19 with the following options: --end-to-end --very-sensitive --no-mixed --no-discordant -q --phred33 -I 10 -X 700. Peaks were called using SEACR version 1.3 after combining replicates. We used custom scripts (https://github.com/mpmeers/JanssensEtAl_MPAL) to merge bulk histone modification-specific peak sets, map fragments to merged peak sets and generate PCA and *t*-SNE plots. All PCA was implemented using the prcomp() function in R version 4.0.0 (https://www.r-project.org/). *t*-SNE was implemented using the Rtsne() function in Rtsne library version 0.15. We used all principal components explaining greater than 1% of variance as input to Rtsne, and perplexity was set to the nearest integer to the square root of the number of rows in the input matrix. Bivalent gene classifications (H3K4me3 specific, H3K27me3 specific and bivalent) for each cell type were determined by quantifying the number of reads mapping in a 2-kb window around the TSS for every gene and using a two-component Gaussian mixture model as implemented using the normalmixEM() function from mixtools library version 1.2.0 in R to distinguish ‘enriched’ and ‘non-enriched’ sets of genes for each histone mark. Bivalent genes were designated as residing in the enriched Gaussian component for both H3K4me3 and H3K27me3 in the cell type in question.

### Identifying *KMT2A*r oncoprotein targets

To identify unique *KMT2A*r targets, we first generated merged sets of SEACR peaks originating from either N-terminal or C-terminal KMT2A antibody-targeted CUT&RUN in each cell type assayed. We quantified the number of fragments mapping to each peak *i* from each dataset *j* and summed reads mapped from the two antibodies targeting the same KMT2A terminus in the same dataset to yield N-terminal (*n*_*ij*_) and C-terminal (*c*_*ij*_) fragments mapped in each peak, existing in cell type sets *N*_j_ and *C*_*j*_, respectively. We calculated the cell-type-specific ‘N over C ratio’ (NCR) for each peak as follows:

1 $$\begin{array}{l}\textrm{NCR}_{ij} = \log _{10} \left(\right. ( ( ( {n_{ij}} ) +\min ( {N_j} ) )/( ( {c_{ij}} ) + \min ( {C_j} ) ) ) \\\qquad\quad\ \left.\times \textrm {ECDF}( ( {N_j + C_j} )/2 ) \right)\left.( ( {n_{ij} + c_{ij}} )/2) \right)\end{array}$$

where min(*x*) is the minimum value of *x* across the peak set and ECDF(*y*)(*x*) is the empirical cumulative distribution function of set *y* evaluated at *x*, as implemented in R version 4.0.0 using the ecdf() function. As illustrated in equation (1), ECDF was used to shrink NCR values toward zero in inverse proportion to the mean *n*_*ij*_ + *c*_*ij*_ signal observed in the peak. *KMT2A*r identity was evaluated by fitting a two-component Gaussian mixture model to all NCR_*j*_ and asserting as true any NCR_*ij*_ that was greater than the mean NCR value of NCR_*j*_ at which the two fitted Gaussian distributions intersected. As a second filter, the above Gaussian mixture modeling approach was repeated using peak length as an input, and peak_*ij*_ was considered to be a *KMT2A*r oncoprotein-specific target only when both NCR and peak length met the cutoffs described above. Gaussian mixture modeling was implemented in R using the normalMixEM() function from mixtools library version 1.2.0. For all peaks assigned as *KMT2A*r in any cell type, NCR scores were hierarchically clustered using the hclust() function in R on a Euclidean distance matrix generated by the dist() function.

### *t*-SNE embedding of active and repressed chromatin regions

For histone modification data, peaks were called from merged replicate datasets using SEACR^34^ version 1.3, and peak sets were merged for each modification across all cell types. We generated matrices of raw read counts mapping in each cell type (columns) to merged peaks (rows) for each modification, and we filtered out instances where counts were lower than any count value whose evaluated ECDF was more than 5% diverged from the predicted ECDF value based on a lognormal fit of the data distribution, using the fitdistr() function from MASS library version 7.3-53 with densfun set to lognormal. We then log_10_ transformed the results and rescaled columns to *z* scores. PCA was performed on the resulting transformed matrices using the prcomp() function in R. For *t*-SNE analysis, all principal components contributing greater than 1% of variance were used as input to the Rtsne() function from Rtsne library version 0.15, with perplexity set as the nearest integer to the square root of the number of peaks and check_duplicates set as false. We used the resulting two-dimensional *t*-SNE values as input to the densityClust() function from densityClust library version 0.3 and used that output in the findClusters() function, with rho and delta values set to the 95th percentile of all rho and delta values output from densityClust(), respectively. To generate cluster-average heatmaps, scaled count values were averaged by cluster and the resulting matrix was used as input to the heatmap.2() function from gplots library version 3.1.1. PCA and *t*-SNE plots were generated using ggplot2 library version 3.3.5 (https://ggplot2.tidyverse.org/).

### UMAP embedding of single cells

Single cells that did not meet a minimum number of reads (*n* = 300) or fell below the normal distribution of FRiP values defined by aggregate data were removed. Then, a single-cell count matrix of *N* features, defined by 5-kb windows tiled across the genome, by *M* cells was generated. These matrices were binarized and normalized via latent semantic indexing (LSI)^40^. The normalized count matrix was reduced from *N* dimensions to two dimensions using UMAP and plotted. We generated imputed gene scores using MAGIC^41^ for subsequent analysis. Normalized dispersion was calculated from these gene scores using SCANPY^55^ version 1.6.0.

### Statistical analysis

All comparisons of the normalized AutoCUT&RUN or AutoCUT&Tag signal across peak sets as well as comparisons of normalized dispersion between gene groups were done using two-sample *t* tests (two sided) with the SciPy.stats.ttest_ind() function in Python; *P* values were not corrected for multiple-hypothesis testing. Comparisons between the distributions of wide KMT2A peaks and KMT2A oncoprotein-binding sites across gene annotations were done using Fisher’s exact tests; *P* values were similarly not corrected for multiple-hypothesis testing. H3K4me3 peaks that showed a significant change in H3K4me3 signal in response to treatment with 30 µM of the Menin binding inhibitor VTP50469 were identified by DESeq2 version 1.32.0 using the Wald test. Here *P* values were corrected for multiple-hypothesis testing (adjusted *P* value) in a manner that was proportional to the number of peaks per sample.

### Preparation of figure panels

All heatmaps were generated using DeepTools^56^ version 3.5.0. *t*-SNE plots colored by maximum signal from immunophenotype class were generated using ggplot2 version 3.3.5. All data were analyzed using bash, Python (https://github.com/python) or R version 4.0.0. The following packages were used in Python: Matplotlib version 3.2.2, NumPy version 1.18.5, Pandas version 1.0.5, Scipy version 1.5.0, Scanpy version 1.6.0 and Seaborn version 0.10.1.

### Reporting Summary

Further information on research design is available in the Nature Research Reporting Summary linked to this article.

## Online content

Any methods, additional references, Nature Research reporting summaries, source data, extended data, supplementary information, acknowledgements, peer review information; details of author contributions and competing interests; and statements of data and code availability are available at 10.1038/s41588-021-00941-9.

## Supplementary information

Reporting Summary

Peer Review Information

Supplementary Tables Supplementary Tables 1–3.

## Data Availability

All primary sequencing data have been deposited as paired-end fastq files in the Gene Expression Omnibus under accession code GSE159608.

## Code availability

Custom code^57^ used for calling KMT2A fusion oncoprotein-binding sites and comparing AutoCUT&Tag histone modification profiles by PCA and *t*-SNE as well as code for generating box plots and bar graphs and analyzing single-cell CUT&Tag data is available at https://github.com/mpmeers/JanssensEtAl_MPAL and 10.5281/zenodo.5123505.
