# Supplementary material for: Automated CUT&Tag profiling of chromatin heterogeneity in mixed-lineage leukemia
Source: Nat Genet. 2021 Oct 18;53(11):1586–96. doi: 10.1038/s41588-021-00941-9 (PMC8571097; doi:10.1038/s41588-021-00941-9)
Supplement: Supplementary file 1 — Reporting Summary [file 41588_2021_941_MOESM1_ESM.pdf]

nature research

Corresponding author(s): Janssens et al

Last updated by author(s): July 23 2021

Reporting Summary

Nature Research wishes to improve the reproducibility of the work that we publish. This form provides structure for consistency and transparency in reporting. For further information on Nature Research policies, see our Editorial Policies and the Editorial Policy Checklist.

Statistics

For all statistical analyses, confirm that the following items are present in the figure legend, table legend, main text, or Methods section.

n/a	Confirmed	
☐	☒	The exact sample size (*n*) for each experimental group/condition, given as a discrete number and unit of measurement
☐	☒	A statement on whether measurements were taken from distinct samples or whether the same sample was measured repeatedly
☐	☒	The statistical test(s) used AND whether they are one- or two-sided<br>*Only common tests should be described solely by name; describe more complex techniques in the Methods section.*
☒	☐	A description of all covariates tested
☐	☒	A description of any assumptions or corrections, such as tests of normality and adjustment for multiple comparisons
☐	☒	A full description of the statistical parameters including central tendency (e.g. means) or other basic estimates (e.g. regression coefficient) AND variation (e.g. standard deviation) or associated estimates of uncertainty (e.g. confidence intervals)
☐	☒	For null hypothesis testing, the test statistic (e.g. *F*, *t*, *r*) with confidence intervals, effect sizes, degrees of freedom and *P* value noted<br>*Give P values as exact values whenever suitable.*
☒	☐	For Bayesian analysis, information on the choice of priors and Markov chain Monte Carlo settings
☒	☐	For hierarchical and complex designs, identification of the appropriate level for tests and full reporting of outcomes
☐	☒	Estimates of effect sizes (e.g. Cohen's *d*, Pearson's *r*), indicating how they were calculated

Our web collection on statistics for biologists contains articles on many of the points above.

Software and code

Policy information about availability of computer code

Data collection	The size distributions and molar concentration of libraries were determined using an Agilent 4200 TapeStation. Up to 48 barcoded CUT&RUN libraries or 96 barcoded CUT&Tag libraries were pooled at approximately equimolar concentration for sequencing. Paired-end 25×25 bp sequencing on the Illumina HiSeq 2500 platform was performed by the Fred Hutchinson Cancer Research Center Genomics Shared Resources. This yielded 5-10 million reads per antibody. Single-cell CUT&Tag libararies were prepared using unique i5 and i7 barcodes and pooled with bulk samples for sequencing. For 500-100 cells 20 million reads was sufficient to obtain an average of approximately 80% saturation of the estimated library size for each single cell. Paired-end reads were aligned using Bowtie2 version 2.3.4.3 to UCSC HG19 with options: --end-to-end --very-sensitive --no-mixed --no-discordant -q --phred33 -I 10 -X 700.
Data analysis	Bedtools version 2.28.0; Deeptools version 3.5.0; Python Packages Used: Numpy version 1.18.5, Pandas version 1.0.5, Seaborn version 0.10.1, Matplotlib version 3.2.2, umap version 0.5, SciPy version 1.5.0, ScanPy version 1.6.0; R version 4.0.0, R libraries used: MASS version 7.3-53, ggplot2 version 3.3.5, Rtsne version 0.15, densityClust version 0.3, gplots version 3.1.1, heatmap3, RColorBrewer version 1.1-2; Custom code available at https://github.com/mpmeers/JanssensEtAl_MPAL. DOI: 10.5281/zenodo.5123505

For manuscripts utilizing custom algorithms or software that are central to the research but not yet described in published literature, software must be made available to editors and reviewers. We strongly encourage code deposition in a community repository (e.g. GitHub). See the Nature Research guidelines for submitting code & software for further information.

Data

Policy information about availability of data

All manuscripts must include a data availability statement. This statement should provide the following information, where applicable:

- Accession codes, unique identifiers, or web links for publicly available datasets
- A list of figures that have associated raw data
- A description of any restrictions on data availability

All primary sequencing data have been deposited as paried-end fastq files in Gene Expression Omnibus under the accession code GSE159608.

Field-specific reporting

Please select the one below that is the best fit for your research. If you are not sure, read the appropriate sections before making your selection.

☒ Life sciences ☐ Behavioural & social sciences ☐ Ecological, evolutionary & environmental sciences

For a reference copy of the document with all sections, see nature.com/documents/nr-reporting-summary-flat.pdf

Life sciences study design

All studies must disclose on these points even when the disclosure is negative.

Sample size	In this study we collected chromatin profiling data for comkparative analysis from 3 contorl samples (CD34+ hematopoietic stem and progenitor cells, H1 human embryonic stem cells, and K562 leukemia cells) as well as 4 KMT2Ar leukemia cell lines (ML-2, SEM, RS4;11, KOPN-8) and 8 KMT2Ar primary patient leukemia samples
Data exclusions	Sequencing reads mapping to the mitochondrial genome were removed from all datasets. This was pre-established and is standard practice in the field. The purpose of this study was to perform comparative analysis of chromatin profiles from the nuclear genome and this can be confounded by variable read numbers from the mitochondrial genome.
Replication	At least 2 biological replicates were profiled. All attempts at replication were successful.
Randomization	n/a. The data and analysis for this study is objective and not prone to influence by the researchers bias.
Blinding	n/a. The data and analysis for this study is objective and not prone to influence by researchers bias.

Reporting for specific materials, systems and methods

We require information from authors about some types of materials, experimental systems and methods used in many studies. Here, indicate whether each material, system or method listed is relevant to your study. If you are not sure if a list item applies to your research, read the appropriate section before selecting a response.

Materials & experimental systems

n/a	Involved in the study	
☐	☒	Antibodies
☐	☒	Eukaryotic cell lines
☒	☐	Palaeontology and archaeology
☒	☐	Animals and other organisms
☐	☒	Human research participants
☒	☐	Clinical data
☒	☐	Dual use research of concern

Methods

n/a	Involved in the study	
☐	☒	ChIP-seq
☒	☐	Flow cytometry
☒	☐	MRI-based neuroimaging

Antibodies

Antibodies used

mouse monoclonal anti-KMT2A (1:100, Millipore, clone N4.4, Cat# 05-764),
rabbit monoclonal anti-KMT2A (1:100, Cell Signaling Tech, clone D2M7U, Cat# 14689S),
mouse monoclonal anti-KMT2A (1:100, Millipore, clone 9-12, Cat# 05-765),
mouse monoconal anti-KMT2A (1:100, Santa Cruz, clone H-10, Cat# sc-374392),
rabbit anti-Mouse IgG (1:100, Abcam Cat# ab46540),
rabbit polyclonal anti-Menin (1:50, Bethyl Cat# A300-105A),
rabbit monoclonal anti-ENL (1:50, Cell Signaling Tech, clone D9M4B, Cat# 14893S),
rabbit monoclonal anti-Dot1L (1:50, Cell Signaling Tech, clone D4O2T, Cat# 90878S),
rabbit oligoclonal anti-H3K4me1 (1:100, Thermo Cat# 710795),
rabbit polyclonal anti-H3K4me3 (1:100 for bulk profiling or 1:10 for single-cell experiments, Active Motif Cat# 39159),

	rabbit polyclonal anti-H3K36me3 (1:100 for bulk profiling or 1:10 for single-cell experiments, Epicypher Cat# 13-0031), rabbit monoclonal anti-H3K27me3 (1:100 for bulk profiling or 1:10 for single-cell experiments, Cell Signaling Technologies, clone C36B11, Cat# 9733S), rabbit polyclonal anti-H3K9me3 (1:100, Abcam Cat# ab8898), rabbit monoclonal anti-H3K27ac (1:50 Millipore, clone RM172, Cat# MABE647), rabbit monoclonal anti-H4K16ac (1:50, Abcam Cat# ab109463), rabbit monoclonal anti-RNAPIISer5P (1:100, Cell Signaling Technologies, clone D9N5I, Cat# 13523), guinea pig anti-rabbit IgG (1:100, antibodies-online Cat# ABIN101961).
Validation	All antibodies are commercially available, and have been verified by Western blotting or by peptide ELISA described on the manufacturer's specification sheets. All antibodies used in this study are confirmed to recognize the human protein as stated on the manufactures website.

Eukaryotic cell lines

Policy information about cell lines

Cell line source(s)	Human K562 cells were purchased from ATCC (Manassas, VA, Cat# CCL-243), H1 hESCs were obtained from WiCell (Cat# WA01-lot# WB35186), The KMT2Ar cell lines ML-2, KOPN-8, RS4;11 and SEM were obtained from the Bleakley lab at the Fred Hutchinson Cancer Research Center
Authentication	Our profiling confirmed both the presence of the KMT2A-fusion protein and the lineage designation of each KMT2Ar cell line (SEM, RS;411, KOPN-8 and ML-2).
Mycoplasma contamination	All cell lines were confirmed as mycoplasma negative on a tri-monthly basis.
Commonly misidentified lines (See ICLAC register)	No commonly misidentified lines were used in this study.

Human research participants

Policy information about studies involving human research participants

Population characteristics	All patient samples were obtained by St. Jude Children's Research Hospital or member COG institutions and in accordance with the Declaration of Helsinki after written consent from the parents/guardians of minors upon enrolling in the trial.
Recruitment	Patients did not receive compensation for participation in this study.
Ethics oversight	The studies were overseen by the Institutional Review Boards at Fred Hutchinson Cancer Research Center (IR Protocol # 9950) and St. Jude Children's Research Hospital.

Note that full information on the approval of the study protocol must also be provided in the manuscript.

ChIP-seq

Data deposition

☒ Confirm that both raw and final processed data have been deposited in a public database such as GEO.

☒ Confirm that you have deposited or provided access to graph files (e.g. BED files) for the called peaks.

Data access links<br>*May remain private before publication.*	All primary sequencing data have been deposited as paired-end fastq files in Gene Expression Omnibus under the accession code GSE159608 (https://www.ncbi.nlm.nih.gov/geo/query/acc.cgi?acc=GSE159608).
Files in database submission	H1_IgG_AutoCUTnRUN(DJ_HsEc_IgG_HA_0705)<br>H1_KMT2A_N1_AutoCUTnRUN(JS_HsEc_042820_JFS1339)<br>H1_KMT2A_N2_AutoCUTnRUN(DJ_HsEc_MLD2_HA_0705)<br>H1_KMT2A_C1_AutoCUTnRUN(DJ_HsEc_MLSp_HA_0705)<br>H1_KMT2A_C2_AutoCUTnRUN(DJ_HsEc_MLMp_HA_0705)<br>SEM_IgG_AutoCUTnRUN(JS_HsEc_031020_JFS1298)<br>SEM_KMT2A_N1_AutoCUTnRUN(JS_HsEc_031020_JFS1292)<br>SEM_KMT2A_N2_AutoCUTnRUN(JS_HsEc_031020_JFS1293)<br>SEM_KMT2A_C1_AutoCUTnRUN(JS_HsEc_031020_JFS1294)<br>SEM_KMT2A_C2_AutoCUTnRUN(JS_HsEc_031020_JFS1295)<br>RS411_IgG_AutoCUTnRUN(JS_HsEc_031020_JFS1312)<br>RS411_KMT2A_N1_AutoCUTnRUN(JS_HsEc_031020_JFS1306)<br>RS411_KMT2A_N2_AutoCUTnRUN(JS_HsEc_031020_JFS1307)<br>RS411_KMT2A_C1_AutoCUTnRUN(JS_HsEc_031020_JFS1308)<br>RS411_KMT2A_C2_AutoCUTnRUN(JS_HsEc_031020_JFS1309)<br>KOPN8_IgG_AutoCUTnRUN(JS_HsEc_031020_JFS1319)<br>KOPN8_KMT2A_N1_AutoCUTnRUN(JS_HsEc_031020_JFS1313)<br>KOPN8_KMT2A_N2_AutoCUTnRUN(JS_HsEc_031020_JFS1314)<br>KOPN8_KMT2A_C1_AutoCUTnRUN(JS_HsEc_031020_JFS1315)

KOPN8_KMT2A_C2_AutoCUTnRUN(JS_HsEc_031020_JFS1316)
ALL1_IgG_AutoCUTnRUN(JS_HsEc_042820_JFS1376)
ALL1_KMT2A_N1_AutoCUTnRUN(JS_HsEc_042820_JFS1377)
ALL1_KMT2A_N2_AutoCUTnRUN(JS_HsEc_042820_JFS1378)
ALL1_KMT2A_C1_AutoCUTnRUN(JS_HsEc_042820_JFS1379)
ALL1_KMT2A_C2_AutoCUTnRUN(JS_HsEc_042820_JFS1380)
AML1_IgG_AutoCUTnRUN(JS_HsEc_042820_JFS1344)
AML1_KMT2A_N1_AutoCUTnRUN(JS_HsEc_042820_JFS1345)
AML1_KMT2A_N2_AutoCUTnRUN(JS_HsEc_042820_JFS1346)
AML1_KMT2A_C1_AutoCUTnRUN(JS_HsEc_042820_JFS1347)
AML1_KMT2A_C2_AutoCUTnRUN(JS_HsEc_042820_JFS1348)
AML2_IgG_AutoCUTnRUN(JS_HsEc_042820_JFS1360)
AML2_KMT2A_N1_AutoCUTnRUN(JS_HsEc_042820_JFS1361)
AML2_KMT2A_N2_AutoCUTnRUN(JS_HsEc_042820_JFS1362)
AML2_KMT2A_C1_AutoCUTnRUN(JS_HsEc_042820_JFS1363)
AML2_KMT2A_C2_AutoCUTnRUN(JS_HsEc_042820_JFS1364)
MPAL1_IgG_AutoCUTnRUN(JS_HsEc_042820_JFS1368)
MPAL1_KMT2A_N1_AutoCUTnRUN(JS_HsEc_042820_JFS1369)
MPAL1_KMT2A_N2_AutoCUTnRUN(JS_HsEc_042820_JFS1370)
MPAL1_KMT2A_C1_AutoCUTnRUN(JS_HsEc_042820_JFS1371)
MPAL1_KMT2A_C2_AutoCUTnRUN(JS_HsEc_042820_JFS1372)
MPAL2_IgG_AutoCUTnRUN(JS_HsEc_042820_JFS1352)
MPAL2_KMT2A_N1_AutoCUTnRUN(JS_HsEc_042820_JFS1353)
MPAL2_KMT2A_N2_AutoCUTnRUN(JS_HsEc_042820_JFS1354)
MPAL2_KMT2A_C1_AutoCUTnRUN(JS_HsEc_042820_JFS1355)
MPAL2_KMT2A_C2_AutoCUTnRUN(JS_HsEc_042820_JFS1356)
K562_IgG_AutoCUTnTag(DJ_Hs_IgG_K5_0310)
K562_H3K27me3_AutoCUTnTag(DJ_Hs_K27m_K5_0310)
K562_H3K27me3_CUTnTag(SH_Hs_K562_K27me3_0416)
K562_H3K4me3_AutoCUTnTag(DJ_Hs_K4m3_K5_0310)
K562_H3K4me3_CUTnTag(SH_Hs_K5x_H3K4me3_0505)
K562_H3K4me1_AutoCUTnTag(DJ_Hs_K4m1_K5_0310)
K562_H3K4me1_CUTnTag(SH_Hs_K5x_H3K4me1_0505)
K562_H3K36me3_AutoCUTnTag(DJ_Hs_K36m3_K5_0310)
K562_H3K36me3_CUTnTag(SH_Hs_K562_K36me3T_0419)
K562_H3K9me3_AutoCUTnTag(DJ_Hs_K9m3_K5_0310)
K562_H3K9me3_CUTnTag(SH_Hs_K562_K9me3_0419)
K562_Dot1L_CUTnTag_Rep1(SH_Hs_K5x_Dot1L_0505)
K562_Dot1L_CUTnTag_Rep2(SH_Hs_K5n_Dot1L_0505)
K562_ELL_CUTnTag_Rep1(SH_Hs_K5x_ELL_0505)
K562_ELL_CUTnTag_Rep2(SH_Hs_K5n_ELL_0505)
K562_ENL_CUTnTag_Rep1(SH_Hs_K5x_MLLENL_0505)
K562_ENL_CUTnTag_Rep2(SH_Hs_K5n_MLLENL_0505)
SEM_IgG_AutoCUTnTag(DJ_Hs_IgG_SE_0310)
SEM_H3K27me3_AutoCUTnTag(DJ_Hs_K27m_SE_0310)
SEM_H3K27me3_CUTnTag(SH_Hs_SEM_K27me3_0416)
SEM_H3K4me3_AutoCUTnTag(DJ_Hs_K4m3_SE_0310)
SEM_H3K4me3_CUTnTag(SH_Hs_SEM_H3K4me3_0502)
SEM_H3K4me1_AutoCUTnTag(DJ_Hs_K4m1_SE_0310)
SEM_H3K4me1_CUTnTag(SH_Hs_SEM_K4me1T_0418)
SEM_H3K36me3_AutoCUTnTag(DJ_Hs_K36m3_SE_0310)
SEM_H3K36me3_CUTnTag_Rep1(SH_Hs_SEM_H3K36me3E_0419)
SEM_H3K36me3_CUTnTag_Rep2(SH_Hs_SEM_H3K36me3T_0419)
SEM_H3K9me3_AutoCUTnTag(DJ_Hs_K9m3_SE_0310)
SEM_H3K9me3_CUTnTag(SH_Hs_SEM_K9me3_0419)
SEM_Dot1L_CUTnTag(SH_Hs_SEM_Dot1_D4O_0502)
SEM_ELL_CUTnTag(SH_Hs_SEM_ELL_0502)
SEM_ENL_CUTnTag(SH_Hs_SEM_MLL_ENL_0502)
RS411_IgG_AutoCUTnTag(DJ_Hs_IgG_RS_0310)
RS411_H3K27me3_AutoCUTnTag(DJ_Hs_K27m_RS_0310)
RS411_H3K27me3_CUTnTag(SH_Hs_R5411_K27me3_0416)
RS411_H3K4me3_AutoCUTnTag(DJ_Hs_K4m3_RS_0310)
RS411_H3K4me3_CUTnTag(SH_Hs_R5411_K4me3_0416)
RS411_H3K4me1_AutoCUTnTag(DJ_Hs_K4m1_RS_0310)
RS411_H3K4me1_CUTnTag(SH_Hs_R5411_K4me1E_0418)
RS411_H3K36me3_AutoCUTnTag(DJ_Hs_K36m3_RS_0310)
RS411_H3K36me3_CUTnTag_Rep1(SH_Hs_R5411_H3K36me3E_0419)
RS411_H3K36me3_CUTnTag_Rep2(SH_Hs_R5411_H3K36me3T_0419)
RS411_H3K9me3_AutoCUTnTag(DJ_Hs_K9m3_RS_0310)
RS411_H3K9me3_CUTnTag(SH_Hs_R5411_K9me3_0419)
RS411_Dot1L_CUTnTag(SH_Hs_RS411_Dot1_D4O_0502)
RS411_ELL_CUTnTag(SH_Hs_RS411_ELL_0502)
RS411_ENL_CUTnTag(SH_Hs_RS411_MLL_ENL_0502)
KOPN8_IgG_AutoCUTnTag(DJ_Hs_IgG_KO_0310)
KOPN8_H3K27me3_AutoCUTnTag(DJ_Hs_K27m_KO_0310)
KOPN8_H3K27me3_CUTnTag(SH_Hs_KOPN8_K27me3_0416)

KOPN8_H3K4me3_AutoCUTnTag(DJ_Hs_K4m3_KO_0310)
KOPN8_H3K4me3_CUTnTag(SH_Hs_KOPN8_K4me3_0416)
KOPN8_H3K4me1_AutoCUTnTag(DJ_Hs_K4m1_KO_0310)
KOPN8_H3K4me1_CUTnTag(SH_Hs_KOPN8_K4me1E_0418)
KOPN8_H3K36me3_AutoCUTnTag(DJ_Hs_K36m3_KO_0310)
KOPN8_H3K36me3_CUTnTag_Rep1(SH_Hs_KOPN8_H3K36me3E_0419)
KOPN8_H3K36me3_CUTnTag_Rep2(SH_Hs_KOPN8_H3K36me3T_0419)
KOPN8_H3K9me3_AutoCUTnTag(DJ_Hs_K9m3_KO_0310)
KOPN8_H3K9me3_CUTnTag(SH_Hs_KOPN8_K9me3_0419)
ML2_IgG_AutoCUTnTag(DJ_Hs_IgG_ML_0310)
ML2_H3K27me3_AutoCUTnTag(DJ_Hs_K27m_ML_0310)
ML2_H3K27me3_CUTnTag(SH_Hs_ML_2_K27me3_0416)
ML2_H3K4me3_AutoCUTnTag(DJ_Hs_K4m3_ML_0310)
ML2_H3K4me3_CUTnTag(SH_Hs_ML_2_K4me3_0416)
ML2_H3K4me1_AutoCUTnTag(DJ_Hs_K4m1_ML_0310)
ML2_H3K4me1_CUTnTag(SH_Hs_ML_2_K4me1E_0418)
ML2_H3K36me3_AutoCUTnTag(DJ_Hs_K36m3_ML_0310)
ML2_H3K36me3_CUTnTag_Rep1(SH_Hs_ML_2_H3K36me3E_0419)
ML2_H3K36me3_CUTnTag_Rep2(SH_Hs_ML_2_H3K36me3T_0419)
ML2_H3K9me3_AutoCUTnTag(DJ_Hs_K9m3_ML_0310)
ML2_H3K9me3_CUTnTag(SH_Hs_ML_2_K9me3_0419)
AML1_IgG_AutoCUTnTag_Rep1(DJ_Hs_IgG_A4_0323)
AML1_IgG_AutoCUTnTag_Rep2(DJ_Hs_IgG_A4_0520)
AML1_H3K27me3_AutoCUTnTag_Rep1(DJ_Hs_K27m_A4_0323)
AML1_H3K27me3_AutoCUTnTag_Rep2(DJ_Hs_K27m_A4_0520)
AML1_H3K4me3_AutoCUTnTag_Rep1(DJ_Hs_K4m3_A4_0323)
AML1_H3K4me3_AutoCUTnTag_Rep2(DJ_Hs_K4m3_A4_0520)
AML1_H3K4me1_AutoCUTnTag_Rep1(DJ_Hs_K4m1_A4_0323)
AML1_H3K4me1_AutoCUTnTag_Rep2(DJ_Hs_K4m1_A4_0520)
AML1_H3K36me3_AutoCUTnTag_Rep1(DJ_Hs_K36m_A4_0323)
AML1_H3K36me3_AutoCUTnTag_Rep2(DJ_Hs_K36m3_A4_0520)
AML1_H3K9me3_AutoCUTnTag_Rep1(DJ_Hs_K9m3_A4_0323)
AML1_H3K9me3_AutoCUTnTag_Rep2(DJ_Hs_K9m3_A4_0520)
AML1_Dot1L_AutoCUTnTag(DJ_Hs_Dot1_A4_0520)
AML1_ENL_AutoCUTnTag(DJ_Hs_ENL_A4_0520)
AML1_ELL_AutoCUTnTag(DJ_Hs_ELL_A4_0520)
MPAL2_IgG_AutoCUTnTag_Rep1(DJ_Hs_IgG_A5_0323)
MPAL2_IgG_AutoCUTnTag_Rep2(DJ_Hs_IgG_A5_0520)
MPAL2_H3K27me3_AutoCUTnTag_Rep1(DJ_Hs_K27m_A5_0323)
MPAL2_H3K27me3_AutoCUTnTag_Rep2(DJ_Hs_K27m_A5_0520)
MPAL2_H3K4me3_AutoCUTnTag_Rep1(DJ_Hs_K4m3_A5_0323)
MPAL2_H3K4me3_AutoCUTnTag_Rep2(DJ_Hs_K4m3_A5_0520)
MPAL2_H3K4me1_AutoCUTnTag_Rep1(DJ_Hs_K4m1_A5_0323)
MPAL2_H3K4me1_AutoCUTnTag_Rep2(DJ_Hs_K4m1_A5_0520)
MPAL2_H3K36me3_AutoCUTnTag_Rep1(DJ_Hs_K36m_A5_0323)
MPAL2_H3K36me3_AutoCUTnTag_Rep2(DJ_Hs_K36m3_A5_0520)
MPAL2_H3K9me3_AutoCUTnTag_Rep1(DJ_Hs_K9m3_A5_0323)
MPAL2_H3K9me3_AutoCUTnTag_Rep2(DJ_Hs_K9m3_A5_0520)
MPAL2_Dot1L_AutoCUTnTag(DJ_Hs_Dot1_A5_0520)
MPAL2_ENL_AutoCUTnTag(DJ_Hs_ENL_A5_0520)
MPAL2_ELL_AutoCUTnTag(DJ_Hs_ELL_A5_0520)
AML2_IgG_AutoCUTnTag_Rep1(DJ_Hs_IgG_A6_0323)
AML2_IgG_AutoCUTnTag_Rep2(DJ_Hs_IgG_A6_0520)
AML2_H3K27me3_AutoCUTnTag_Rep1(DJ_Hs_K27m_A6_0323)
AML2_H3K27me3_AutoCUTnTag_Rep2(DJ_Hs_K27m_A6_0520)
AML2_H3K4me3_AutoCUTnTag_Rep1(DJ_Hs_K4m3_A6_0323)
AML2_H3K4me3_AutoCUTnTag_Rep2(DJ_Hs_K4m3_A6_0520)
AML2_H3K4me1_AutoCUTnTag_Rep1(DJ_Hs_K4m1_A6_0323)
AML2_H3K4me1_AutoCUTnTag_Rep2(DJ_Hs_K4m1_A6_0520)
AML2_H3K36me3_AutoCUTnTag_Rep1(DJ_Hs_K36m_A6_0323)
AML2_H3K36me3_AutoCUTnTag_Rep2(DJ_Hs_K36m3_A6_0520)
AML2_H3K9me3_AutoCUTnTag_Rep1(DJ_Hs_K9m3_A6_0323)
AML2_H3K9me3_AutoCUTnTag_Rep2(DJ_Hs_K9m3_A6_0520)
AML2_Dot1L_AutoCUTnTag(DJ_Hs_Dot1_A6_0520)
AML2_ENL_AutoCUTnTag(DJ_Hs_ENL_A6_0520)
AML2_ELL_AutoCUTnTag(DJ_Hs_ELL_A6_0520)
MPAL1_IgG_AutoCUTnTag_Rep1(DJ_Hs_IgG_TB11_0323)
MPAL1_IgG_AutoCUTnTag_Rep2(DJ_Hs_IgG_TB11_0520)
MPAL1_H3K27me3_AutoCUTnTag_Rep1(DJ_Hs_K27m_TB11_0323)
MPAL1_H3K27me3_AutoCUTnTag_Rep2(DJ_Hs_K27m_TB11_0520)
MPAL1_H3K4me3_AutoCUTnTag_Rep1(DJ_Hs_K4m3_TB11_0323)
MPAL1_H3K4me3_AutoCUTnTag_Rep2(DJ_Hs_K4m3_TB11_0520)
MPAL1_H3K4me1_AutoCUTnTag_Rep1(DJ_Hs_K4m1_TB11_0323)
MPAL1_H3K4me1_AutoCUTnTag_Rep2(DJ_Hs_K4m1_TB11_0520)
MPAL1_H3K36me3_AutoCUTnTag_Rep1(DJ_Hs_K36m_TB11_0323)
MPAL1_H3K36me3_AutoCUTnTag_Rep2(DJ_Hs_K36m3_TB11_0520)

MPAL1_H3K9me3_AutoCUTnTag_Rep1(DJ_Hs_K9m3_TB11_0323)
MPAL1_H3K9me3_AutoCUTnTag_Rep2(DJ_Hs_K9m3_TB11_0520)
MPAL1_Dot1L_AutoCUTnTag(DJ_Hs_Dot1_TB11_0520)
MPAL1_ENL_AutoCUTnTag(DJ_Hs_ENL_TB11_0520)
MPAL1_ELL_AutoCUTnTag(DJ_Hs_ELL_TB11_0520)
ALL1_IgG_AutoCUTnTag_Rep1(DJ_Hs_IgG_TB13_0323)
ALL1_IgG_AutoCUTnTag_Rep2(DJ_Hs_IgG_TB13_0520)
ALL1_H3K27me3_AutoCUTnTag_Rep1(DJ_Hs_K27m_TB13_0323)
ALL1_H3K27me3_AutoCUTnTag_Rep2(DJ_Hs_K27m_TB13_0520)
ALL1_H3K4me3_AutoCUTnTag_Rep1(DJ_Hs_K4m3_TB13_0323)
ALL1_H3K4me3_AutoCUTnTag_Rep2(DJ_Hs_K4m3_TB13_0520)
ALL1_H3K4me1_AutoCUTnTag_Rep1(DJ_Hs_K4m1_TB13_0323)
ALL1_H3K4me1_AutoCUTnTag_Rep2(DJ_Hs_K4m1_TB13_0520)
ALL1_H3K36me3_AutoCUTnTag_Rep1(DJ_Hs_K36m_TB13_0323)
ALL1_H3K36me3_AutoCUTnTag_Rep2(DJ_Hs_K36m3_TB13_0520)
ALL1_H3K9me3_AutoCUTnTag_Rep1(DJ_Hs_K9m3_TB13_0323)
ALL1_H3K9me3_AutoCUTnTag_Rep2(DJ_Hs_K9m3_TB13_0520)
ALL1_Dot1L_AutoCUTnTag(DJ_Hs_Dot1_TB13_0520)
ALL1_ENL_AutoCUTnTag(DJ_Hs_ENL_TB13_0520)
ALL1_ELL_AutoCUTnTag(DJ_Hs_ELL_TB13_0520)
CD34_IgG_AutoCUTnRUN(DJ_Hs_IgG_CD34_1208)
CD34_KMT2A_N1_AutoCUTnRUN(DJ_Hs_MLL_N1_CD34_1208)
CD34_KMT2A_N2_AutoCUTnRUN(DJ_Hs_MLL_N2_CD34_1208)
CD34_KMT2A_C1_AutoCUTnRUN(DJ_Hs_MLL_C1_CD34_1208)
CD34_KMT2A_C2_AutoCUTnRUN(DJ_Hs_MLL_C1_CD34_1208)
K562_IgG_AutoCUTnRUN(DJ_Hs_IgG_K5_1208)
K562_KMT2A_N1_AutoCUTnRUN(DJ_Hs_MLL_N1_K5_1208)
K562_KMT2A_N2_AutoCUTnRUN(DJ_Hs_MLL_N2_K5_1208)
K562_KMT2A_C1_AutoCUTnRUN(DJ_Hs_MLL_C1_K5_1208)
K562_KMT2A_C2_AutoCUTnRUN(DJ_Hs_MLL_C2_K5_1208)
AML3_IgG_AutoCUTnRUN(DJ_Hs_IgG_A107C_1208)
AML3_KMT2A_N1_AutoCUTnRUN(DJ_Hs_MLL_N1_A107C_1208)
AML3_KMT2A_N2_AutoCUTnRUN(DJ_Hs_MLL_N2_A107C_1208)
AML3_KMT2A_C1_AutoCUTnRUN(DJ_Hs_MLL_C1_A107C_1208)
AML3_KMT2A_C2_AutoCUTnRUN(DJ_Hs_MLL_C2_A107C_1208)
AML4_IgG_AutoCUTnRUN(DJ_Hs_IgG_A384C_1208)
AML4_KMT2A_N1_AutoCUTnRUN(DJ_Hs_MLL_N1_A384C_1208)
AML4_KMT2A_N2_AutoCUTnRUN(DJ_Hs_MLL_N2_A384C_1208)
AML4_KMT2A_C1_AutoCUTnRUN(DJ_Hs_MLL_C1_A384C_1208)
AML4_KMT2A_C2_AutoCUTnRUN(DJ_Hs_MLL_C2_A384C_1208)
AML5_IgG_AutoCUTnRUN(DJ_Hs_IgG_A109C_1208)
AML5_KMT2A_N1_AutoCUTnRUN(DJ_Hs_MLL_N1_A109C_1208)
AML5_KMT2A_N2_AutoCUTnRUN(DJ_Hs_MLL_N2_A109C_1208)
AML5_KMT2A_C1_AutoCUTnRUN(DJ_Hs_MLL_C1_A109C_1208)
AML5_KMT2A_C2_AutoCUTnRUN(DJ_Hs_MLL_C2_A109C_1208)
ML2_IgG_AutoCUTnRUN(JS_HsEc_031020_JFS1305)
ML2_KMT2A_N1_AutoCUTnRUN(JS_HsEc_031020_JFS1299)
ML2_KMT2A_N2_AutoCUTnRUN(JS_HsEc_031020_JFS1300)
ML2_KMT2A_C1_AutoCUTnRUN(JS_HsEc_031020_JFS1301)
ML2_KMT2A_C2_AutoCUTnRUN(JS_HsEc_031020_JFS1302)
CD34_IgG_AutoCUTnTag_Rep1(DJ_Hs_CD34_IgG_1_0101)
CD34_IgG_AutoCUTnTag_Rep2(DJ_Hs_CD34_IgG_2_0101)
CD34_H3K4me3_AutoCUTnTag_Rep1(DJ_Hs_CD34_K4m3_1_0101)
CD34_H3K4me3_AutoCUTnTag_Rep2(DJ_Hs_CD34_K4m3_2_0101)
CD34_H3K4me1_AutoCUTnTag_Rep1(DJ_Hs_CD34_K4m1_1_0101)
CD34_H3K4me1_AutoCUTnTag_Rep2(DJ_Hs_CD34_K4m1_2_0101)
CD34_H3K36me3_AutoCUTnTag_Rep1(DJ_Hs_CD34_K36m3_1_0101)
CD34_H3K36me3_AutoCUTnTag_Rep2(DJ_Hs_CD34_K36m3_2_0101)
CD34_H3K27ac_AutoCUTnTag_Rep1(DJ_Hs_CD34_K27ac_1_0101)
CD34_H3K27ac_AutoCUTnTag_Rep2(DJ_Hs_CD34_K27ac_2_0101)
CD34_H4K16ac_AutoCUTnTag_Rep1(DJ_Hs_CD34_K16ac_1_0101)
CD34_H4K16ac_AutoCUTnTag_Rep2(DJ_Hs_CD34_K16ac_2_0101)
CD34_H3K27me3_AutoCUTnTag_Rep1(DJ_Hs_CD34_K27m_1_0101)
CD34_H3K27me3_AutoCUTnTag_Rep2(DJ_Hs_CD34_K27m_2_0101)
CD34_H3K9me3_AutoCUTnTag_Rep1(DJ_Hs_CD34_K9m3_1_0101)
CD34_H3K9me3_AutoCUTnTag_Rep2(DJ_Hs_CD34_K9m3_2_0101)
CD34_RNAP2S5p_AutoCUTnTag_Rep1(DJ_Hs_CD34_Pol2s5_1_0101)
CD34_RNAP2S5p_AutoCUTnTag_Rep2(DJ_Hs_CD34_Pol2s5_2_0101)
CD34_Dot1L_AutoCUTnTag_Rep1(DJ_Hs_CD34_Dot1L_1_0101)
CD34_Dot1L_AutoCUTnTag_Rep2(DJ_Hs_CD34_Dot1L_2_0101)
CD34_ENL_AutoCUTnTag_Rep1(DJ_Hs_CD34_ENL_1_0101)
CD34_ENL_AutoCUTnTag_Rep2(DJ_Hs_CD34_ENL_2_0101)
AML3_IgG_AutoCUTnTag_Rep1(DJ_Hs_A107_IgG_1_0101)
AML3_IgG_AutoCUTnTag_Rep2(DJ_Hs_A107_IgG_2_0101)
AML3_H3K4me3_AutoCUTnTag_Rep1(DJ_Hs_A107_K4m3_1_0101)
AML3_H3K4me3_AutoCUTnTag_Rep2(DJ_Hs_A107_K4m3_2_0101)

AML3_H3K4me1_AutoCUTnTag_Rep1(DJ_Hs_A107_K4m1_1_0101)
AML3_H3K4me1_AutoCUTnTag_Rep2(DJ_Hs_A107_K4m1_2_0101)
AML3_H3K36me3_AutoCUTnTag_Rep1(DJ_Hs_A107_K36m3_1_0101)
AML3_H3K36me3_AutoCUTnTag_Rep2(DJ_Hs_A107_K36m3_2_0101)
AML3_H3K27ac_AutoCUTnTag_Rep1(DJ_Hs_A107_K27ac_1_0101)
AML3_H3K27ac_AutoCUTnTag_Rep2(DJ_Hs_A107_K27ac_2_0101)
AML3_H4K16ac_AutoCUTnTag_Rep1(DJ_Hs_A107_K16ac_1_0101)
AML3_H4K16ac_AutoCUTnTag_Rep2(DJ_Hs_A107_K16ac_2_0101)
AML3_H3K27me3_AutoCUTnTag_Rep1(DJ_Hs_A107_K27m_1_0101)
AML3_H3K27me3_AutoCUTnTag_Rep2(DJ_Hs_A107_K27m_2_0101)
AML3_H3K9me3_AutoCUTnTag_Rep1(DJ_Hs_A107_K9m3_1_0101)
AML3_H3K9me3_AutoCUTnTag_Rep2(DJ_Hs_A107_K9m3_2_0101)
AML3_RNAP2S5p_AutoCUTnTag_Rep1(DJ_Hs_A107_Pol2s5_1_0101)
AML3_RNAP2S5p_AutoCUTnTag_Rep2(DJ_Hs_A107_Pol2s5_2_0101)
AML3_Dot1L_AutoCUTnTag_Rep1(DJ_Hs_A107_Dot1L_1_0101)
AML3_Dot1L_AutoCUTnTag_Rep2(DJ_Hs_A107_Dot1L_2_0101)
AML3_ENL_AutoCUTnTag_Rep1(DJ_Hs_A107_ENL_1_0101)
AML3_ENL_AutoCUTnTag_Rep2(DJ_Hs_A107_ENL_2_0101)
AML4_IgG_AutoCUTnTag_Rep1(DJ_Hs_A384_IgG_1_0101)
AML4_IgG_AutoCUTnTag_Rep2(DJ_Hs_A384_IgG_2_0101)
AML4_H3K4me3_AutoCUTnTag_Rep1(DJ_Hs_A384_K4m3_1_0101)
AML4_H3K4me3_AutoCUTnTag_Rep2(DJ_Hs_A384_K4m3_2_0101)
AML4_H3K4me1_AutoCUTnTag_Rep1(DJ_Hs_A384_K4m1_1_0101)
AML4_H3K4me1_AutoCUTnTag_Rep2(DJ_Hs_A384_K4m1_2_0101)
AML4_H3K36me3_AutoCUTnTag_Rep1(DJ_Hs_A384_K36m3_1_0101)
AML4_H3K36me3_AutoCUTnTag_Rep2(DJ_Hs_A384_K36m3_2_0101)
AML4_H3K27ac_AutoCUTnTag_Rep1(DJ_Hs_A384_K27ac_1_0101)
AML4_H3K27ac_AutoCUTnTag_Rep2(DJ_Hs_A384_K27ac_2_0101)
AML4_H4K16ac_AutoCUTnTag_Rep1(DJ_Hs_A384_K16ac_1_0101)
AML4_H4K16ac_AutoCUTnTag_Rep2(DJ_Hs_A384_K16ac_2_0101)
AML4_H3K27me3_AutoCUTnTag_Rep1(DJ_Hs_A384_K27m_1_0101)
AML4_H3K27me3_AutoCUTnTag_Rep2(DJ_Hs_A384_K27m_2_0101)
AML4_H3K9me3_AutoCUTnTag_Rep1(DJ_Hs_A384_K9m3_1_0101)
AML4_H3K9me3_AutoCUTnTag_Rep2(DJ_Hs_A384_K9m3_2_0101)
AML4_RNAP2S5p_AutoCUTnTag_Rep1(DJ_Hs_A384_Pol2s5_1_0101)
AML4_RNAP2S5p_AutoCUTnTag_Rep2(DJ_Hs_A384_Pol2s5_2_0101)
AML4_Dot1L_AutoCUTnTag_Rep1(DJ_Hs_A384_Dot1L_1_0101)
AML4_Dot1L_AutoCUTnTag_Rep2(DJ_Hs_A384_Dot1L_2_0101)
AML4_ENL_AutoCUTnTag_Rep1(DJ_Hs_A384_ENL_1_0101)
AML4_ENL_AutoCUTnTag_Rep2(DJ_Hs_A384_ENL_2_0101)
AML5_IgG_AutoCUTnTag_Rep1(DJ_Hs_A109_IgG_1_0101)
AML5_IgG_AutoCUTnTag_Rep2(DJ_Hs_A109_IgG_2_0101)
AML5_H3K4me3_AutoCUTnTag_Rep1(DJ_Hs_A109_K4m3_1_0101)
AML5_H3K4me3_AutoCUTnTag_Rep2(DJ_Hs_A109_K4m3_2_0101)
AML5_H3K4me1_AutoCUTnTag_Rep1(DJ_Hs_A109_K4m1_1_0101)
AML5_H3K4me1_AutoCUTnTag_Rep2(DJ_Hs_A109_K4m1_2_0101)
AML5_H3K36me3_AutoCUTnTag_Rep1(DJ_Hs_A109_K36m3_1_0101)
AML5_H3K36me3_AutoCUTnTag_Rep2(DJ_Hs_A109_K36m3_2_0101)
AML5_H3K27ac_AutoCUTnTag_Rep1(DJ_Hs_A109_K27ac_1_0101)
AML5_H3K27ac_AutoCUTnTag_Rep2(DJ_Hs_A109_K27ac_2_0101)
AML5_H4K16ac_AutoCUTnTag_Rep1(DJ_Hs_A109_K16ac_1_0101)
AML5_H4K16ac_AutoCUTnTag_Rep2(DJ_Hs_A109_K16ac_2_0101)
AML5_H3K27me3_AutoCUTnTag_Rep1(DJ_Hs_A109_K27m_1_0101)
AML5_H3K27me3_AutoCUTnTag_Rep2(DJ_Hs_A109_K27m_2_0101)
AML5_H3K9me3_AutoCUTnTag_Rep1(DJ_Hs_A109_K9m3_1_0101)
AML5_H3K9me3_AutoCUTnTag_Rep2(DJ_Hs_A109_K9m3_2_0101)
AML5_RNAP2S5p_AutoCUTnTag_Rep1(DJ_Hs_A109_Pol2s5_1_0101)
AML5_RNAP2S5p_AutoCUTnTag_Rep2(DJ_Hs_A109_Pol2s5_2_0101)
AML5_Dot1L_AutoCUTnTag_Rep1(DJ_Hs_A109_Dot1L_1_0101)
AML5_Dot1L_AutoCUTnTag_Rep2(DJ_Hs_A109_Dot1L_2_0101)
AML5_ENL_AutoCUTnTag_Rep1(DJ_Hs_A109_ENL_1_0101)
AML5_ENL_AutoCUTnTag_Rep2(DJ_Hs_A109_ENL_2_0101)
AML1_Dot1L_AutoCUTnTag_Rep2(DJ_Hs_Dot1L_A4_2_0125)
AML1_ENL_AutoCUTnTag_Rep2(DJ_Hs_ENL_A4_2_0125)
AML1_RNAP2S5p_AutoCUTnTag_Rep1(DJ_Hs_P2S5_A4_0323)
AML1_RNAP2S5p_AutoCUTnTag_Rep2(DJ_Hs_Pol2S5_A4_2_0125)
AML1_H3K27ac_AutoCUTnTag_Rep1(DJ_Hs_K27ac_A4_1_0125)
AML1_H3K27ac_AutoCUTnTag_Rep2(DJ_Hs_K27ac_A4_2_0125)
AML1_H4K16ac_AutoCUTnTag_Rep1(DJ_Hs_K16ac_A4_1_0125)
AML1_H4K16ac_AutoCUTnTag_Rep2(DJ_Hs_K16ac_A4_2_0125)
MPAL2_Dot1L_AutoCUTnTag_Rep2(DJ_Hs_Dot1L_A5_2_0125)
MPAL2_ENL_AutoCUTnTag_Rep2(DJ_Hs_ENL_A5_2_0125)
MPAL2_RNAP2S5p_AutoCUTnTag_Rep1(DJ_Hs_P2S5_A5_0323)
MPAL2_RNAP2S5p_AutoCUTnTag_Rep2(DJ_Hs_Pol2S5_A5_2_0125)
MPAL2_H3K27ac_AutoCUTnTag_Rep1(DJ_Hs_K27ac_A5_1_0125)
MPAL2_H3K27ac_AutoCUTnTag_Rep2(DJ_Hs_K27ac_A5_2_0125)

MPAL2_H4K16ac_AutoCUTnTag_Rep1(DJ_Hs_K16ac_A5_1_0125)
MPAL2_H4K16ac_AutoCUTnTag_Rep2(DJ_Hs_K16ac_A5_2_0125)
AML2_Dot1L_AutoCUTnTag_Rep2(DJ_Hs_Dot1L_A6_2_0125)
AML2_ENL_AutoCUTnTag_Rep2(DJ_Hs_ENL_A6_2_0125)
AML2_RNAP2S5p_AutoCUTnTag_Rep1(DJ_Hs_P2S5_A6_0323)
AML2_RNAP2S5p_AutoCUTnTag_Rep2(DJ_Hs_Pol2S5_A6_2_0125)
AML2_H3K27ac_AutoCUTnTag_Rep1(DJ_Hs_K27ac_A6_1_0125)
AML2_H3K27ac_AutoCUTnTag_Rep2(DJ_Hs_K27ac_A6_2_0125)
AML2_H4K16ac_AutoCUTnTag_Rep1(DJ_Hs_K16ac_A6_1_0125)
AML2_H4K16ac_AutoCUTnTag_Rep2(DJ_Hs_K16ac_A6_2_0125)
MPAL1_Dot1L_AutoCUTnTag_Rep2(DJ_Hs_Dot1L_TB11_2_0125)
MPAL1_ENL_AutoCUTnTag_Rep2(DJ_Hs_ENL_TB11_2_0125)
MPAL1_RNAP2S5p_AutoCUTnTag_Rep1(DJ_Hs_P2S5_TB11_0323)
MPAL1_RNAP2S5p_AutoCUTnTag_Rep2(DJ_Hs_Pol2S5_TB11_2_0125)
MPAL1_H3K27ac_AutoCUTnTag_Rep1(DJ_Hs_K27ac_TB11_1_0125)
MPAL1_H3K27ac_AutoCUTnTag_Rep2(DJ_Hs_K27ac_TB11_2_0125)
MPAL1_H4K16ac_AutoCUTnTag_Rep1(DJ_Hs_K16ac_TB11_1_0125)
MPAL1_H4K16ac_AutoCUTnTag_Rep2(DJ_Hs_K16ac_TB11_2_0125)
ALL1_Dot1L_AutoCUTnTag_Rep2(DJ_Hs_Dot1L_TB13_2_0125)
ALL1_ENL_AutoCUTnTag_Rep2(DJ_Hs_ENL_TB13_2_0125)
ALL1_RNAP2S5p_AutoCUTnTag_Rep1(DJ_Hs_P2S5_TB13_0323)
ALL1_RNAP2S5p_AutoCUTnTag_Rep2(DJ_Hs_Pol2S5_TB13_2_0125)
ALL1_H3K27ac_AutoCUTnTag_Rep1(DJ_Hs_K27ac_TB13_1_0125)
ALL1_H3K27ac_AutoCUTnTag_Rep2(DJ_Hs_K27ac_TB13_2_0125)
ALL1_H4K16ac_AutoCUTnTag_Rep1(DJ_Hs_K16ac_TB13_1_0125)
ALL1_H4K16ac_AutoCUTnTag_Rep2(DJ_Hs_K16ac_TB13_2_0125)
SEM_H4K16ac_AutoCUTnTag_Rep1(DJ_Hs_K16ac_SEM_1_0125)
SEM_H4K16ac_AutoCUTnTag_Rep2(DJ_Hs_K16ac_SEM_2_0125)
RS411_H4K16ac_AutoCUTnTag_Rep1(DJ_Hs_K16ac_RS411_1_0125)
RS411_H4K16ac_AutoCUTnTag_Rep2(DJ_Hs_K16ac_RS411_2_0125)
KOPN8_H4K16ac_AutoCUTnTag_Rep1(DJ_Hs_K16ac_KOPN8_1_0125)
KOPN8_H4K16ac_AutoCUTnTag_Rep2(DJ_Hs_K16ac_KOPN8_2_0125)
ML2_H3K27ac_AutoCUTnTag_Rep1(DJ_Hs_K27ac_ML2_1_0125)
ML2_H3K27ac_AutoCUTnTag_Rep2(DJ_Hs_K27ac_ML2_2_0125)
SEM_IgG_DMSO_AutoCUTnTag_Rep1(DJ_Hs_IgG_SE_DMSO_1_0119)
SEM_IgG_DMSO_AutoCUTnTag_Rep2(DJ_Hs_IgG_SE_DMSO_2_0119)
SEM_H3K27ac_DMSO_AutoCUTnTag_Rep1(DJ_Hs_K27ac_SE_DMSO_1_0119)
SEM_H3K27ac_DMSO_AutoCUTnTag_Rep2(DJ_Hs_K27ac_SE_DMSO_2_0119)
SEM_H3K27ac_EPZ5676_AutoCUTnTag_Rep1(DJ_Hs_K27ac_SE_EPZ_1_0119)
SEM_H3K27ac_EPZ5676_AutoCUTnTag_Rep2(DJ_Hs_K27ac_SE_EPZ_2_0119)
SEM_H3K4me3_DMSO_AutoCUTnTag_Rep1(DJ_Hs_K4me3_SE_DMSO_1_0119)
SEM_H3K4me3_DMSO_AutoCUTnTag_Rep2(DJ_Hs_K4me3_SE_DMSO_2_0119)
SEM_H3K4me3_EPZ5676_AutoCUTnTag_Rep1(DJ_Hs_K4me3_SE_EPZ_1_0119)
SEM_H3K4me3_EPZ5676_AutoCUTnTag_Rep2(DJ_Hs_K4me3_SE_EPZ_2_0119)
SEM_H3K4me3_VTP50469_AutoCUTnTag_Rep1(DJ_Hs_K4me3_SE_VTP_1_0119)
SEM_H3K4me3_VTP50469_AutoCUTnTag_Rep2(DJ_Hs_K4me3_SE_VTP_2_0119)
SEM_RNAP2S5p_AutoCUTnTag(DJ_Hs_PolS5p_SE_0310)
SEM_RNAP2S5p_DMSO_AutoCUTnTag_Rep1(DJ_Hs_Pol2S5_SE_DMSO_1_0119)
SEM_RNAP2S5p_DMSO_AutoCUTnTag_Rep2(DJ_Hs_Pol2S5_SE_DMSO_2_0119)
KOPN8_IgG_DMSO_AutoCUTnTag_Rep1(DJ_Hs_IgG_KO_DMSO_1_0119)
KOPN8_IgG_DMSO_AutoCUTnTag_Rep2(DJ_Hs_IgG_KO_DMSO_2_0119)
KOPN8_H3K27ac_DMSO_AutoCUTnTag_Rep1(DJ_Hs_K27ac_KO_DMSO_1_0119)
KOPN8_H3K27ac_DMSO_AutoCUTnTag_Rep2(DJ_Hs_K27ac_KO_DMSO_2_0119)
KOPN8_H3K27ac_EPZ5676_AutoCUTnTag_Rep1(DJ_Hs_K27ac_KO_EPZ_1_0119)
KOPN8_H3K27ac_EPZ5676_AutoCUTnTag_Rep2(DJ_Hs_K27ac_KO_EPZ_2_0119)
KOPN8_H3K4me3_DMSO_AutoCUTnTag_Rep1(DJ_Hs_K4me3_KO_DMSO_1_0119)
KOPN8_H3K4me3_DMSO_AutoCUTnTag_Rep2(DJ_Hs_K4me3_KO_DMSO_2_0119)
KOPN8_H3K4me3_EPZ5676_AutoCUTnTag_Rep1(DJ_Hs_K4me3_KO_EPZ_1_0119)
KOPN8_H3K4me3_EPZ5676_AutoCUTnTag_Rep2(DJ_Hs_K4me3_KO_EPZ_2_0119)
KOPN8_H3K4me3_VTP50469_AutoCUTnTag_Rep1(DJ_Hs_K4me3_KO_VTP_1_0119)
KOPN8_H3K4me3_VTP50469_AutoCUTnTag_Rep2(DJ_Hs_K4me3_KO_VTP_2_0119)
KOPN8_RNAP2S5p_AutoCUTnTag(DJ_Hs_PolS5p_KO_0310)
KOPN8_RNAP2S5p_DMSO_AutoCUTnTag_Rep1(DJ_Hs_Pol2S5_KO_DMSO_1_0119)
KOPN8_RNAP2S5p_DMSO_AutoCUTnTag_Rep1(DJ_Hs_Pol2S5_KO_DMSO_2_0119)
RS411_IgG_DMSO_AutoCUTnTag_Rep1(DJ_Hs_IgG_RS_DMSO_1_0119)
RS411_IgG_DMSO_AutoCUTnTag_Rep2(DJ_Hs_IgG_RS_DMSO_2_0119)
RS411_H3K27ac_DMSO_AutoCUTnTag_Rep1(DJ_Hs_K27ac_RS_DMSO_1_0119)
RS411_H3K27ac_DMSO_AutoCUTnTag_Rep2(DJ_Hs_K27ac_RS_DMSO_2_0119)
RS411_H3K27ac_EPZ5676_AutoCUTnTag_Rep1(DJ_Hs_K27ac_RS_EPZ_1_0119)
RS411_H3K27ac_EPZ5676_AutoCUTnTag_Rep2(DJ_Hs_K27ac_RS_EPZ_2_0119)
RS411_H3K4me3_DMSO_AutoCUTnTag_Rep1(DJ_Hs_K4me3_RS_DMSO_1_0119)
RS411_H3K4me3_DMSO_AutoCUTnTag_Rep2(DJ_Hs_K4me3_RS_DMSO_2_0119)
RS411_H3K4me3_EPZ5676_AutoCUTnTag_Rep1(DJ_Hs_K4me3_RS_EPZ_1_0119)
RS411_H3K4me3_EPZ5676_AutoCUTnTag_Rep2(DJ_Hs_K4me3_RS_EPZ_2_0119)
RS411_H3K4me3_VTP50469_AutoCUTnTag_Rep1(DJ_Hs_K4me3_RS_VTP_1_0119)
RS411_H3K4me3_VTP50469_AutoCUTnTag_Rep2(DJ_Hs_K4me3_RS_VTP_2_0119)

RS411_RNAP2S5p_AutoCUTnTag(DJ_Hs_PolS5p_RS_0310)
RS411_RNAP2S5p_DMSO_AutoCUTnTag_Rep1(DJ_Hs_Pol2S5_RS_DMSO_1_0119)
RS411_RNAP2S5p_DMSO_AutoCUTnTag_Rep1(DJ_Hs_Pol2S5_RS_DMSO_2_0119)
SEM_Menin_AutoCUTnTag_Rep1(DJ_Hs_Men1_SE_DMSO_0112)
SEM_Menin_AutoCUTnTag_Rep2(DJ_Hs_Men2_SE_DMSO_0112)
RS411_Menin_AutoCUTnTag_Rep1(DJ_Hs_Men1_RS_DMSO_0112)
RS411_Menin_AutoCUTnTag_Rep2(DJ_Hs_Men2_RS_DMSO_0112)
KOPN8_Menin_AutoCUTnTag_Rep1(DJ_Hs_Men1_KO_DMSO_0112)
KOPN8_Menin_AutoCUTnTag_Rep2(DJ_Hs_Men2_KO_DMSO_0112)
SEM_Dot1L_AutoCUTnTag_Rep2(DJ_Hs_Dot1L_SE_1104)
SEM_ENL_AutoCUTnTag_Rep2(DJ_Hs_ENL_SE_1104)
RS411_Dot1L_AutoCUTnTag_Rep2(DJ_Hs_Dot1L_RS_1104)
RS411_ENL_AutoCUTnTag_Rep2(DJ_Hs_ENL_RS_1104)
KOPN8_Dot1L_AutoCUTnTag_Rep1(DJ_Hs_Dot1L_KO_1104)
KOPN8_Dot1L_AutoCUTnTag_Rep2(DJ_Hs_Dot1L_2_KO_1104)
KOPN8_ENL_AutoCUTnTag_Rep1(DJ_Hs_ENL_KO_1104)
KOPN8_ENL_AutoCUTnTag_Rep2(DJ_Hs_ENL_2_KO_1104)
SEM_PolCUTAC(SH_Hs_SEM_PolS5hex_1123)
RS411_PolCUTAC(SH_Hs_RS411_PolS5hex_1123)
KOPN8_PolCUTAC(SH_Hs_KOPN8x_PolS5hex_1123)

Genome browser session (e.g. UCSC)

https://genome.ucsc.edu/s/djanssen/Janssens_Figure_1b
https://genome.ucsc.edu/s/djanssen/Janssens_Figure_2a
https://genome.ucsc.edu/s/djanssen/Janssens_Figure_4k
https://genome.ucsc.edu/s/djanssen/Janssens_Supplementary_Figure_1b
https://genome.ucsc.edu/s/djanssen/Janssens_Supplementary_Figure_4c

Methodology

Replicates

At least 2 replicates were performed. Comparative analysis is the topic of this manuscript.

Sequencing depth

All Experiments were paired-end. Sequencing depths and sampling is reported in the manuscript.

Antibodies

All antibodies and sources are provided in the Methods section.

Peak calling parameters

SEACR version 1.3, relaxed mode, “norm” mode.

Data quality

Data quality assessment is the topic of this manuscript, and is reported.

Software

Bedtools version 2.28.0; Deeptools version 3.5.0; Python Packages Used: Numpy version 1.18.5, Pandas version 1.0.5, Seaborn version 0.10.1, Matplotlib version 3.2.2, umap version 0.5, SciPy version 1.5.0, ScanPy version 1.6.0; R version 4.0.0, R libraries used: MASS version 7.3-53, ggplot2 version 3.3.5, Rtsne version 0.15, densityClust version 0.3, gplots version 3.1.1, heatmap3, RColorBrewer version 1.1-2; Custom code available at https://github.com/mpmeers/JanssensEtAl_MPAL. DOI: 10.5281/zenodo.5123505
