# Supplementary material for: Automated CUT&Tag profiling of chromatin heterogeneity in mixed-lineage leukemia
Source: Nat Genet. 2021 Oct 18;53(11):1586–96. doi: 10.1038/s41588-021-00941-9 (PMC8571097; doi:10.1038/s41588-021-00941-9)
Supplement: Supplementary file 2 — Peer Review Information [file 41588_2021_941_MOESM2_ESM.pdf]

Peer Review Information

Journal: Nature Genetics
Manuscript Title: Automated CUT&Tag profiling of chromatin heterogeneity in mixed-lineage leukemia
Corresponding author name(s): Dr Steven Henikoff

Reviewer Comments & Decisions:

Decision Letter, initial version:

17th Nov 2020

Dear Steve,

Your Article, "Automated CUT&Tag profiling of chromatin heterogeneity in mixed-lineage leukemia" has now been seen by 3 referees. You will see from their comments copied below that while they find your work of considerable potential interest, they have raised quite substantial concerns that must be addressed. In light of these comments, we cannot accept the manuscript for publication, but would be very interested in considering a revised version that addresses these serious concerns.

We hope you will find the referees' comments useful as you decide how to proceed. If you wish to submit a substantially revised manuscript, please bear in mind that we will be reluctant to approach the referees again in the absence of major revisions.

In brief, all three reviewers appreciated (to varying extents) the methodological advances, both experimental and computational, presented by your study; however, they all also found the biological novelty to insufficient, at this stage, for publication at Nature Genetics.

Referee #1 thinks that the methods will be "of broad interest in cancer biology" but the analysis is under-developed. They suggest a number of new experiments (e.g. looking at MLL1-deficient cells, drug treatments) and analysis (e.g. for histone sc data: comparing to scATAC/RNA data, combinatorial analysis).
Referee #2 clearly appreciates the technical improvements in these techniques, but thinks the computational analysis should be much more rigorously quantitative. They also make some specific suggestions for new analyses.
Referee #3 quite clearly think thats, in its current state, the manuscript lacks the novel biological

insights required. They also make a number of important suggestions for further work (more controls, functional/mechanistic details of the SEPT6 fusion or of the novel KMT2A fusion targets).

Editorially, we agree that this shortfall of novel biological insight is important and should be addressed in a revision. We think the suggestions for the computational analysis (e.g. making it more quantitative, and doing combinatorial rather than single-factor histone mark analysis) are constructive and would add to the impact of the study. We also concur that further functional experiments would enhance the biological novelty; the referees make a number of useful suggestions, and we would leave it to you and your co-authors in deciding which to pursue.

To guide the scope of the revisions, the editors discuss the referee reports in detail within the team, including with the chief editor, with a view to identifying key priorities that should be addressed in revision and sometimes overruling referee requests that are deemed beyond the scope of the current study. We hope that you will find the prioritised set of referee points to be useful when revising your study. Please do not hesitate to get in touch if you would like to discuss these issues further.

If you choose to revise your manuscript taking into account all reviewer and editor comments, please highlight all changes in the manuscript text file. At this stage we will need you to upload a copy of the manuscript in MS Word .docx or similar editable format.

We are committed to providing a fair and constructive peer-review process. Do not hesitate to contact us if there are specific requests from the reviewers that you believe are technically impossible or unlikely to yield a meaningful outcome.

If revising your manuscript:

*1) Include a “Response to referees” document detailing, point-by-point, how you addressed each referee comment. If no action was taken to address a point, you must provide a compelling argument. This response will be sent back to the referees along with the revised manuscript.

*2) If you have not done so already please begin to revise your manuscript so that it conforms to our Article format instructions, available here.
Refer also to any guidelines provided in this letter.

*3) Include a revised version of any required Reporting Summary:
https://www.nature.com/documents/nr-reporting-summary.pdf
It will be available to referees (and, potentially, statisticians) to aid in their evaluation if the manuscript goes back for peer review.
A revised checklist is essential for re-review of the paper.

Please be aware of our guidelines on digital image standards.

You may use the link below to submit your revised manuscript and related files:

[REDACTED]

Note: This URL links to your confidential home page and associated information about manuscripts you may have submitted, or that you are reviewing for us. If you wish to forward this email to co-authors, please delete the link to your homepage.

If you wish to submit a suitably revised manuscript we would hope to receive it within 6 months. If you cannot send it within this time, please let us know. We will be happy to consider your revision so long as nothing similar has been accepted for publication at Nature Genetics or published elsewhere. Should your manuscript be substantially delayed without notifying us in advance and your article is eventually published, the received date would be that of the revised, not the original, version.

Please do not hesitate to contact me if you have any questions or would like to discuss the required revisions further.

Nature Genetics is committed to improving transparency in authorship. As part of our efforts in this direction, we are now requesting that all authors identified as 'corresponding author' on published papers create and link their Open Researcher and Contributor Identifier (ORCID) with their account on the Manuscript Tracking System (MTS), prior to acceptance. ORCID helps the scientific community achieve unambiguous attribution of all scholarly contributions. You can create and link your ORCID from the home page of the MTS by clicking on 'Modify my Springer Nature account'. For more information please visit please visit www.springernature.com/orcid.

Thank you for the opportunity to review your work.

Sincerely,

Michael Fletcher, PhD
Associate Editor, Nature Genetics

ORCID: 0000-0003-1589-7087

Referee expertise:

Referee #1: KMT2A fusions in haematopoesis/leukaemia

Referee #2: single-cell chromatin methods, cancer

Referee #3: MLLs, cancer, chromatin

Reviewers' Comments:

Reviewer #1:
Remarks to the Author:
In the manuscript "Automated CUT & TAG profiling of chromatin heterogeneity in mixed lineage leukemia" the authors develop and improve upon methods that localize chromatin proteins in small numbers of cells. The study starts out using a paradigm of MLL fusion oncoprotein binding in cell lines

where there is significant prior knowledge from ChIP-seq and other approaches used in published studies. They use first the cut & run approach to identify fusion oncoprotein binding sites in 3 cell lines and 5 primary leukemia specimens. This approach includes an emphasis on new computational methods to distinguish MLL proto-oncogene sites from MLL fusion oncoprotein sites. Having designated these 2 groups of genes, they include an analysis of the colocalization of particular fusion partner-recruited components (SEC/DOT1L) with either the fusion protein or its proto-oncogene counterpart. These do provide a new perspective on both biochemical and prior ChIP-seq investigation of a) whether the proto-oncogene plays a role in regulating leukemogenic programs and b) whether recruitment of transcriptional effector proteins/complexes are unique to the fusion oncoprotein or specific to particular fusion partners. The data further corroborate and add to prior observations that fusion oncoproteins exhibit a more spread out, gene body localization that the proto-oncoprotein. In addition to these basic observations, the authors use the identity of the specific oncogene binding sites to sort the cell lines and specimens in PCA space and make the argument that the fusion partner plays an active role the distribution on chromatin and thus in determining the leukemia subtype or lineage, a concept that is supported by large analyses of patient specimens. Co-incident chromatin features are then analyzed with several conclusions being made from the leukemia-type specific findings (see below). Finally the single cell cut & tag approach is optimized and applied to cell lines and primary samples to examine tumor heterogeneity. The authors focus on particular chromatin marks and their correlation with lineage-specific gene programs for analyzing primary leukemia specimen heterogeneity and make the case that fusion partner and lineage are reflected in the distinct H3K4me3 patterns. Strengths of this study include the new, very rigorous experimental/computational methods which will be of broad interest in cancer biology, but weaknesses include the application of these methods to discover new features of this particular disease.

Major critiques:

A) The data presented in Figure 1 is a new approach that very rigorously defines MLL1 proto-oncogene binding sites as distinct from MLL-fusion oncoproteins sites in a series of cell lines used by many in the field. The authors provide important data that addresses some controversies in this field regarding a) the distinctions between proto-oncogene and oncogene binding sites, b) the relative enrichment of H3K4me3 modifications at either set of targets, and c) the relative enrichment of transcriptional effector components in cells comparing the proto- versus fusion-oncogene sets of binding sites. Further, they corroborate an observation of gene-body spreading that is specific to the oncogene.

However, these data are not developed as fully as they could be to provide novel information regarding oncogene and proto-oncogene function. The following 4 points focus on areas in which the authors could contribute something new to the understanding of this particular leukemia genetic network:

1) The observation of decreased H3K4me3 signal at oncogene sites (Fig 2a) brings an implication that the oncogene binding displaces the WT proto-oncogene binding. Given that the authors define anti-MLL-N sites as either oncogene or proto-oncogene bound, can the authors comment on the existence of co-occupied sites? It has been reported using ChIP-seq that the two proteins bind distinct sites (Cao et al. Cell Discovery 2016) and on the other hand that they compete with each other for binding sites (Liang et al. Cell 2017). Therefore discussing how the authors interpret their data with respect to this controversy would be helpful.

2) Could the decreased H3K4me3 signal at oncogene bound sites simply reflect the gene-body biased

distribution that the authors show, rather than the TSS-oriented distribution that they show for the proto-oncogene? An analysis to rule out this bias in the box and whisker plots really should be shown or a description of how the authors corrected for this expected bias should be emphasized.

3) There is an assumption that the association of the H3K4me3 enrichment with the proto-oncogene MLL rather than the oncogene reflects its own catalytic activity. Another interpretation of this correlation is that the H3K4me3 binding domain (PHD3) in the wild type protein (not present in the oncoprotein) would be responsible for this association. The authors should comment on this, or better, address experimentally in the following point:

4) The authors miss an opportunity to definitively comment on the interaction between wild type protein and fusion oncoproteins since they do not perform the hit & run approach using an MLL1-deficient cell type, such as the ML-2 cell line. I understand that the definition of "wild-type sites" is technically impossible in this line, but could they use the definition from a related AML to define such sites? If not, it would not be too difficult to generate an MLL1 Cas9/CRISPR knockout version of one of the cell lines profiled in this study.

B) The use of the definition of unique MLL-fusion oncoprotein sites in the different cell lines and specimens to argue that the fusion partner "likely influences the tumor specific localization" is not very strongly supported by data in Fig. 1, which, as the authors point out, could be equally explained by cell of origin bias. The claim made could be substantiated through experimental introduction of different fusion oncoproteins coupled with this analysis, but as presented the sample sizes are small and diverse history of the cell lines/human specimens precludes anything but a correlation.

C) It seems that a major value of the single cell method (auto cut & tag) and the analysis of the results in PCA space is geared toward demonstrating the power of these approaches to diagnose and understand heterogeneity in real primary specimens. Although it is very interesting to see how the different chromatin marks predict cell identity compared to each other, the information gained (that H3K4me3 predicts identity better than H3K27me3 or H3K36me3 for example) is not that new. As far as lymphoid versus myeloid identity goes in the primary samples, other small-scale techniques seem more connected to cell identity/plasticity such as sc-ATAC-seq or sc-RNA-seq. Therefore this aspect of the work is not compelling without showing that the approach outperforms or gives different information than comparable methods that could function at this scale.

Overall the methods developed in this manuscript are very impressive and have the potential to provide new mechanistic insight into the targeting of MLL-related oncoproteins in the genome, as well as highlight the important targetable features in MLL-r leukemia, but the data presented here are more confirmatory of prior observations using different approaches. In addition to the thoughts in section "A" above, the authors could perform the chromatin profiling with some perturbations that might really be insightful, for example, do Menin inhibitors selectively remove the gene body (broad) oncogene pattern? Or do they act on a subset of other (non-fusion target) genes? What impact would MLL1, Dot1L or other inhibitors have on the localization of proto-oncogene/oncogene? Does a combinatorial analyis of chromatin marks predict the lineage fidelity of the primary specimen, when patients are followed long-term?

Small critiques

1) The use of MPAL to describe the MLL-r patient samples is a bit imprecise given they are more characterized than just by co-expression of multiple lineage markers. If the authors were discussing many MPALs that included multiple mutational landscapes, it might make more sense.

2) The use of review citations in the intro is not very precise to the statement being made in many cases. For example the “fetal and adult hematopoiesis” statement cites a very general, mostly biochemical review where there are several (Atunes and Ottersbach comes to mind) that focus on that topic. Similarly the citations referring to biochemical features of the KMT2 family use more biological-oriented papers to cover those statements, so some re-evaluation of the review content might be helpful for accuracy.

3) Similar to the above comment, citation 17, 19-21 all refer to lineage plasticity specifically in MLL-rearranged leukemia but the statement is more broadly about cancer cell lineage infidelity, where there are other non-MLL focused reviews that would cover that statement.

4) Could the authors clarify in the DepMap supplemental data whether they included (in the MLL1 row) guides that target the MLL-fusion oncoprotein itself, or just those that edit beyond the breakpoint of the fusion oncoprotein? Or if this row of the column is intended to show the impact of targeting the fusion oncoprotein, perhaps that should be made clear.

a few typos:

1. page 7, regions-region
2. Page11, heterogenous - heterogeneous
3. page 12, pivotol-pivotal

Reviewer #2:
Remarks to the Author:
Janssens et al. present an automated CUT&Tag approach and a series of datasets to study the genomic binding patterns of WT and translocated KTM2A together with epigenomic landscapes in leukemia, both in vitro and primary patient samples. Their approach is of wide interest as it could be applicable to a variety of cancers and other diseases with altered epigenetic actors, where the genomic binding patterns of these actors have remained unknown due to technical limitations. If the technique is highly promising (could be applicable to a wide range of translational questions), and the datasets impressive in terms of technological challenge they represent and their quality (remarkable signal to noise ratio), I have some major concerns regarding the analysis of the data and the conclusions drawn.

Main Concerns:

1. The absence of quantification and statistical testing from the manuscript makes it difficult to evaluate the conclusions drawn by the authors from their analysis. The terminology remains qualitative throughout the manuscript: “is similar to” without any correlation coefficient and statistical evaluation, “heavily marked with H3K4me3 », « binding sites are relatively depleted for H3K4me3 » with no statistical testing of the signal with proper assumption on the data distribution; « a smaller proportion of H3K4me3 marked features show »with no quantification nor testing.

Such absence of quantification and statistics leads to contradictions and over-statements at several occasions:

- The authors state that " (PCA) of oncogene binding sites indicates that the specific partner in each fusion protein likely influences the tumor-specific localization. », and then state an opposite conclusion at the end of the same paragraph "KMT2A fusion partners are not the sole determinants of oncogene landscapes". In addition, there is no statistical testing to evaluate which statement is more significant than the other, e.g evaluating the association of the variable "fusion partner" to the PC1 or PC2 values.

- The general conclusion that « H3K4me3 is deposited away from the gene promoter » is supported by one snapshot (Fig. 2b), where it should be quantified genome-wide, and statistically tested against WT, and random shuffling of datasets.

- The statement that « Oncoprotein binding sites lack H3K27me3 or H3K9me3 (Fig. 2c,d), but are enriched in H3K4me1 and H3K36me3 » is supported by no quantification/statistics.

2. Histone enrichment profiles are analyzed "in silos", whereas we know that's it is the combination of marks for each locus that determines expression patterns/cell identities. Epigenomic profiles could be analyzed in combination to fully account for the dependencies between them and their combinatorial value in determining cell lineages.

3. The analysis of single-cell datasets is biased by the choice of the annotation to interrogate the data. The authors chose to use the differential elements identified earlier in the study between bulk datasets to interrogate single-cell datasets. The authors make the strong - and unjustified - hypothesis "that the heterogeneous usage of those elements within the same leukemia might underlie the phenotypic plasticity of KMT2Ar leukemia ». Intra and inter heterogeneity won't be necessarily based on differential enrichments of the same sets of loci, this needs to be determined thanks to detailed data analysis, using various annotations for example. Why did the authors not use the single-cell data without any a priori to reveal tumor intra-heterogeneity, rather than forcing the bulk annotation on their single-cell data? Each single-cell dataset could also be analyzed independently to reveal intra-tumor heterogeneity.

In addition, the authors state that "Discrete sample-specific clusters were resolved by UMAP projection of cells profiled for H3K4me3 and H3K27me3 (Fig. 4b,c) …, indicating that the differences in the H3K4me3 and H3K27me3 landscapes of KMT2Ar leukemia cells of the same samples are generally less than the differences between samples. »
But this was completely expected and is due to the initial bias in the annotation for the analysis (see above). The authors have precisely selected their single cell data to only look at regions that differ between samples and not the ones that could differ within one single sample.

Minor concerns:
1/Regarding differences in H3K4me3 between WT and oncogenic sites, could they be due to accessibility issues, could the sites be profiled for accessibility to look into this (H3 profiling, ATAC or other technique)?
2/What about batch effect in the single-cell datasets, could it also explain part of the grouping of cells

within samples ?

Reviewer #3:
Remarks to the Author:
In this manuscript Janssens and colleagues in the Henikoff lab apply a variety of chromatin profiling techniques to characterize a panel of human acute myeloid leukemia (AML) lines and patient samples. Using automated CUT&RUN and automated CUT&TAG technology the authors profile the binding pattern of the KMT2A fusion oncoprotein as well as a panel of histone modifications using low cell numbers or single cells. The authors confirm previously published results showing that KMT2A fusions display unique localization patterns from non-translocated KMT2A. Translocated KMT2A displays an increased co-localization with the H3K4me1 and H3K36me3 marks relative to wildtype KMT2A. Regulatory element clustering and single cell analysis revealed leukemia subtypes and intra-tumor heterogeneity.

While this article presents a number of technically innovative approaches, it does little to provide actionable mechanistic insight into the pathogenesis of AML that could be used to advance the development of therapies. Moreover, some of the findings here regarding differences in KMT2A fusion binding and have already been reported using standard ChIP-seq approaches (Prange et al., 2017. Oncogene “MLL-AF9 and MLL-AF4 oncofusion proteins bind a distinct enhancer repertoire and target the RUNX1 program in 11q23 acute myeloid leukemia”). Therefore, this manuscript lacks the functional/biological studies that would normally be expected of a publication in Nature Genetics. That said, the work is carefully performed and is of high technical quality making it a good fit for a journal such as Nature Methods.

Reviewer comments
1. Defining KMT2A-fusion binding sites as sites detected with the amino-terminal antibody, but not the carboxy-terminal antibody is a reasonable approach, but there are additional controls that would be great to have before being fully convinced of this method. First, it is essential to have more than one KMT2A wildtype cell line used a proof of principle control. The authors use only the H1 human ESC line, how about including human hematopoetic stem cells and non-KMT2A translocated leukemias as controls. Second, depletion experiment (shRNA or CRISPR) to deplete KMT2A and measure whether detected peaks are specific by loss of CUT&RUN signal is also essential. Using an shRNA specific to the KMT2A fusion partner would be a good way to define oncoprotein specific sites.

2. The authors identify a set shared KMT2A oncogene targets including SENP6 and ARID2, which the authors claim are novel dependencies in KMT2A rearranged leukemias based on Cancer Dependency Map (DepMap) data. This is not accurate for SENP6, a common essential gene, whose depletion kills the majority of cancer cell lines in the DepMap database. Similarly, ARID2 is a cancer dependency in many lineages although it does show selectivity for hematopoetic cancers, including AML. However, it does not appear to show a selectivity for KMT2A translocated cancers over other hematopoetic cancers.

3. Relating to point #2, the authors should perform biological functional assays of potential novel important targets of KMT2A by gene depletion experiments. The manuscript would be greatly strengthened if the authors can demonstrate that their chromatin profiling technologies can point toward new therapeutic targets.

4. The characterization of the SEPT6 translocation is interesting and suggests that perhaps this fusion does not interact with the canonical fusion complexes of KMT2A. This is a novel area that the authors could explore. How does gene expression in the SEPT6 translocated cells compare to canonical KMT2A translocations. Understanding the mechanisms governing the pathogenesis of non-SEC/Dotcom KMT2A translocation driven AML would add novelty to this manuscript.

5. With regard to the colocalization of KMT2A fusions with various histone modifications, the association with H3K36me3 (mark of active gene bodies) is not surprising because KMT2A was previously shown to co-localize with H3K79me2, another marker of active gene bodies (Prange et al., 2017). Moreover, KMT2A fusions are most commonly components of transcription elongation complexes that localize to actively transcribed genes.

H3K4me1 is a very broadly distributed mark that occurs both inside of genes and in inter-genic regions. Does KMT2A fusion binding only co-localize with with active gene-body associated H3K4me1, or does it bind to silent H3K4me1 marked intergenic elements? If the former, this is likely not a causative mechanism for recruitment of the fusion but is simply a reflection of the very broad distribution of the H3K4me1 mark in active/poised chromatin.

Do the authors observe any correlation between KMT2A fusion binding and histone acetylation?

6. In their final section the authors propose that KMT2A-AF4 leukemias have greater lineage plasticity. The authors should perform functional experiments to test this.

Author Rebuttal to Initial comments

Reviewer #1:
Remarks to the Author:
In the manuscript “Automated CUT & TAG profiling of chromatin heterogeneity in mixed lineage leukemia” the authors develop and improve upon methods that localize chromatin proteins in small numbers of cells. The study starts out using a paradigm of MLL fusion oncoprotein binding in cell lines where there is significant prior knowledge from ChIP-seq and other approaches used in published studies. They use first the cut & run approach to identify fusion oncoprotein binding sites in 3 cell lines and 5 primary leukemia specimens. This approach includes an emphasis on new computational methods to distinguish MLL proto-oncogene sites from MLL fusion oncoprotein sites. Having designated these 2 groups of genes, they include an analysis of the colocalization of particular fusion partner-recruited components (SEC/DOT1L) with either the fusion protein or its proto-oncogene counterpart. These do provide a new perspective on both biochemical and prior ChIP-seq investigation of a) whether
the proto-oncogene plays a role in regulating leukemogenic programs and b) whether recruitment of transcriptional effector proteins/complexes are unique to the fusion oncoprotein or specific to particular fusion partners. The data further corroborate and add to prior observations that fusion oncoproteins exhibit a more spread out, gene body localization that the proto-oncoprotein. In addition to these basic observations, the authors use the identity of the specific oncogene binding sites to sort the cell lines and specimens in PCA space and make the argument that the fusion partner plays an active role the distribution on chromatin and thus in

determining the leukemia subtype or lineage, a concept that is supported by large analyses of patient specimens. Co-incident chromatin features are then analyzed with several conclusions being made from the leukemia-type specific findings (see below). Finally the single cell cut & tag approach is optimized and applied to cell lines and
primary samples to examine tumor heterogeneity. The authors focus on particular chromatin marks and their correlation with lineage-specific gene programs for analyzing primary leukemia specimen heterogeneity and make the case that fusion partner and lineage are reflected in the distinct H3K4me3 patterns. Strengths of this study include the new, very rigorous experimental/computational methods which will be of broad interest in cancer biology, but weaknesses include the application of these methods to discover new features of this particular disease.

Major critiques:

A) The data presented in Figure 1 is a new approach that very rigorously defines MLL1 proto-oncogene binding sites as distinct from MLL-fusion oncoproteins sites in a series of cell lines used by many in the field. The authors provide important data that addresses some controversies in this field regarding a) the distinctions between proto-oncogene and oncogene binding sites, b) the relative enrichment of H3K4me3 modifications at either set of targets, and c) the relative enrichment of transcriptional effector components in cells comparing the proto- versus fusion-oncogene sets of binding sites. Further, they corroborate an observation of gene-body spreading that is specific to the oncogene.
We are glad the reviewer appreciates the potential impact of these experiments.

However, these data are not developed as fully as they could be to provide novel information regarding oncogene and proto-oncogene function. The following 4 points focus on areas in which the authors could contribute something new to the understanding of this particular leukemia genetic network:

1) The observation of decreased H3K4me3 signal at oncogene sites (Fig 2a) brings an implication that the oncogene binding displaces the WT proto-oncogene binding. Given that the authors define anti-MLL-N sites as either oncogene or proto-oncogene bound, can the authors comment on the existence of co-occupied sites? It has been reported using ChIP-seq that the two proteins bind distinct sites (Cao et al. Cell Discovery 2016) and on the other hand that they compete with each other for binding sites (Liang et al. Cell 2017). Therefore discussing how the authors interpret their data with respect to this controversy would be helpful.
We have added profiling of ML-2 leukemia cells, which lack wildtype KMT2A. These experiments demonstrate binding of the oncofusion protein without binding of any wildtype protein, with chromatin features that we attribute to the oncofusion protein binding. We now refer to the Cao et al (2016) and Liang et al (2017) studies on line 112 of the text. We now show that the oncoprotein displaces the wildtype protein (line 124-127).

2) Could the decreased H3K4me3 signal at oncogene bound sites simply reflect the gene-body biased distribution that the authors show, rather than the TSS-oriented distribution that they show for the proto-oncogene? An analysis to rule out this bias in the box and whisker plots

really should be shown or a description of how the authors corrected for this expected bias should be emphasized.
We agree that gene body biased distribution of the oncofusion protein could account for reduced H3K4me3 signal and so we have reworded the text accordingly (line 176-177).

3) There is an assumption that the association of the H3K4me3 enrichment with the proto-oncogene MLL rather than the oncogene reflects its own catalytic activity. Another interpretation of this correlation is that the H3K4me3 binding domain (PHD3) in the wild type protein (not present in the oncoprotein) would be responsible for this association. The authors should comment on this, or better, address experimentally in the following point:

4) The authors miss an opportunity to definitively comment on the interaction between wild type protein and fusion oncoproteins since they do not perform the hit & run approach using an MLL1-deficient cell type, such as the ML-2 cell line. I understand that the definition of “wild-type sites” is technically impossible in this line, but could they use the definition from a related AML to define such sites? If not, it would not be too difficult to generate an MLL1 Cas9/CRISPR knockout version of one of the cell lines profiled in this study.
To address the question of whether KMT2A methyltransferase activity is responsible for H3K4me3 modification at oncofusion binding sites, we have added new data profiling the ML-2 cell type, which lacks wildtype MLL-1 (Figure 1C & E, as well as Figure 2 C). We show that some fusion protein target promoters remain positive for H3K4me3, implying that a different methyltransferase catalyzes this methylation. We now make this point in the manuscript (line 176-178).

B) The use of the definition of unique MLL-fusion oncoprotein sites in the different cell lines and specimens to argue that the fusion partner “likely influences the tumor specific localization” is not very strongly supported by data in Fig. 1, which, as the authors point out, could be equally explained by cell of origin bias. The claim made could be substantiated through experimental introduction of different fusion oncoproteins coupled with this analysis, but as presented the sample sizes are small and diverse history of the cell lines/human specimens precludes anything but a correlation.
We agree, and we now acknowledge both possibilities in the text (lines 128-144). We have also included three more MLL-rearranged clinical samples, and CD34+ and K562 cell control data sets in order to clarify the relationships between samples.

C) It seems that a major value of the single cell method (auto cut & tag) and the analysis of the results in PCA space is geared toward demonstrating the power of these approaches to diagnose and understand heterogeneity in real primary specimens. Although it is very interesting to see how the different chromatin marks predict cell identity compared to each other, the information gained (that H3K4me3 predicts identity better than H3K27me3 or H3K36me3 for example) is not that new. As far as lymphoid versus myeloid identity goes in the primary samples, other small-scale techniques seem more connected to cell identity/plasticity such as sc-ATAC-seq or sc-RNA-seq. Therefore this aspect of the work is not compelling without showing that the approach outperforms or gives different information than comparable methods that could function at this scale.

We have expanded our analysis of bulk and single cell profiling in two ways, First, by integrating the H3K4me3 and H3K37me3 bulk data we now identify regions of “bivalent” chromatin. Interestingly, many of these regions overlap with oncoprotein target genes (Figure 2k,l). Using our single cell approach we found that H3K4me3 and H3K27me3 vary on oncogene target genes even within a leukemia sample (Figure 3g-j). Because bivalent chromatin is defined by these marks this would not be feasible using either single cell ATAC-seq or RNA-seq. We added new sections to the manuscript describing these results.

Overall the methods developed in this manuscript are very impressive and have the potential to provide new mechanistic insight into the targeting of MLL-related oncoproteins in the genome, as well as highlight the important targetable features in MLL-r leukemia, but the data presented here are more confirmatory of prior observations using different approaches. In addition to the thoughts in section “A” above, the authors could perform the chromatin profiling with some perturbations that might really be insightful, for example, do Menin inhibitors selectively remove the gene body (broad) oncogene pattern? Or do they act on a subset of other (non-fusion target) genes? What impact would MLL1, Dot1L or other inhibitors have on the localization of proto-oncogene/oncogene?
We are happy to added these experiments. We now include drug inhibition studies using Menin and Dot1L inhibitors (Figure 4). We found that CUT&Tag profiling can predict which samples are sensitive to each of these inhibitors by identifying the chromatin distributions of fusion oncoprotein-associated cofactors (Fig. 4k,l). We added a new section 5 to the manuscript describing this work.

Does a combinatorial analyis of chromatin marks predict the lineage fidelity of the primary specimen, when patients are followed long-term?
This is an interesting point, and we have analyzed chromatin features of oncofusion binding sites to address this question. We defined binding sites marked with bivalent chromatin features of H3K4me3 and H3K27me3 marks. In single-cell analysis, many of these sites vary between cells, indicating heterogeneity within a population for gene expression programs. We describe the implications of this for lineage fidelity in the discussion (lines 368-379).

Small critiques

1) The use of MPAL to describe the MLL-r patient samples is a bit imprecise given they are more characterized than just by co-expression of multiple lineage markers. If the authors were discussing many MPALs that included multiple mutational landscapes, it might make more sense.
The clinical samples we profiled have been characterized as MPALs by flow cytometry using the WHO approved markers. We confirmed expression using our chromatin profiling assays, and we provide this information in Supplementary Table 1.

2) The use of review citations in the intro is not very precise to the statement being made in many cases. For example the “fetal and adult hematopoiesis” statement cites a very general,

mostly biochemical review where there are several (Atunes and Ottersbach comes to mind) that focus on that topic. Similarly the citations referring to biochemical features of the KMT2 family use more biological-oriented papers to cover those statements, so some re-evaluation of the review content might be helpful for accuracy.
We have now replaced review citations with the appropriate citations of the primary literature (see lines 41, 43, 50, 53 and 57).

3) Similar to the above comment, citation 17, 19-21 all refer to lineage plasticity specifically in MLL-rearranged leukemia but the statement is more broadly about cancer cell lineage infidelity, where there are other non-MLL focused reviews that would cover that statement.
We have clarified this point on line 57.

4) Could the authors clarify in the DepMap supplemental data whether they included (in the MLL1 row) guides that target the MLL-fusion oncoprotein itself, or just those that edit beyond the breakpoint of the fusion oncoprotein? Or if this row of the column is intended to show the impact of targeting the fusion oncoprotein, perhaps that should be made clear.
We have removed the text and table referring to DepMap data because the substantial functional data we now include makes similar points more directly.

a few typos:

1. page 7, regions-region
2. Page11, heterogenous - heterogeneous
3. page 12, pivotol-pivotal

Fixed

Reviewer #2:
Remarks to the Author:
Janssens et al. present an automated CUT&Tag approach and a series of datasets to study the genomic binding patterns of WT and translocated KTM2A together with epigenomic landscapes in leukemia, both in vitro and primary patient samples. Their approach is of wide interest as it could be applicable to a variety of cancers and other diseases with altered epigenetic actors, where the genomic binding patterns of these actors have remained unknown due to technical limitations. If the technique is highly promising (could be applicable to a wide range of translational questions), and the datasets impressive in terms of technological challenge they represent and their quality (remarkable signal to noise ratio), I have some major concerns regarding the analysis of the data and the conclusions drawn.

Main Concerns:

1. The absence of quantification and statistical testing from the manuscript makes it difficult to evaluate the conclusions drawn by the authors from their analysis. The terminology remains qualitative throughout the manuscript: “is similar to” without any correlation coefficient and statistical evaluation, “heavily marked with H3K4me3 », « binding sites are relatively depleted for H3K4me3 » with no statistical testing of the signal with proper assumption on the data

distribution; « a smaller proportion of H3K4me3 marked features show »with no quantification nor testing.
We now provide statistical testing for all quantitative statements made throughout the manuscript (see below for specifics).

Such absence of quantification and statistics leads to contradictions and over-statements at several occasions:

- The authors state that “ (PCA) of oncogene binding sites indicates that the specific partner in each fusion protein likely influences the tumor-specific localization. », and then state an opposite conclusion at the end of the same paragraph “KMT2A fusion partners are not the sole determinants of oncogene landscapes”. In addition, there is no statistical testing to evaluate which statement is more significant than the other, e.g evaluating the association of the variable “fusion partner” to the PC1 or PC2 values.
We have now reworded this section to clarify that both the lineage and fusion partner contribute to targeting of the oncoprotein (lines 135-138).

- The general conclusion that « H3K4me3 is deposited away from the gene promoter » is supported by one snapshot (Fig. 2b), where it should be quantified genome-wide, and statistically tested against WT, and random shuffling of datasets.
We now summarize genomic profiling for H3K4me3 in 3 samples, showing that the histone modification is enriched in gene bodies at oncofusion binding sites. We compare gene body enrichment between oncofusion target and wildtype KMT2A target sites in Fig. 4j.

- The statement that « Oncoprotein binding sites lack H3K27me3 or H3K9me3 (Fig. 2c,d), but are enriched in H3K4me1 and H3K36me3 » is supported by no quantification/statistics.
We have moved the boxplots the reviewer mentions here to Supplementary Figure 6. Our analysis shows that these two histone modifications are not affected by the oncofusion protein, and so are not specifically enriched around target genes.

2. Histone enrichment profiles are analyzed “in silos”, whereas we know that’s it is the combination of marks for each locus that determines expression patterns/cell identities. Epigenomic profiles could be analyzed in combination to fully account for the dependencies between them and their combinatorial value in determining cell lineages.
We appreciate this helpful critique, and now perform extensive combinatorial analysis for the H3K4me3 and H3K27me3 marks to define bivalent chromatin. This is added as a new section to the Results (line 239-256).

3. The analysis of single-cell datasets is biased by the choice of the annotation to interrogate the data. The authors chose to use the differential elements identified earlier in the study between bulk datasets to interrogate single-cell datasets. The authors make the strong - and unjustified - hypothesis “that the heterogeneous usage of those elements within the same leukemia might underlie the phenotypic plasticity of KMT2Ar leukemia ».

We agree and have refocused our analysis using single cell data to describe the heterogeneity between cells of certain leukemia samples. Our results reveal that sets of genes co-vary between cells, and are related to bivalent chromatin marks at these genes. We elaborate two possible interpretations of this heterogeneity in the Discussion.

Intra and inter heterogeneity won't be necessarily based on differential enrichments of the same sets of loci, this needs to be determined thanks to detailed data analysis, using various annotations for example. Why did the authors not use the single-cell data without any a priori to reveal tumor intra-heterogeneity, rather than forcing the bulk annotation on their single-cell data? Each single-cell dataset could also be analyzed independently to reveal intra-tumor heterogeneity.
We agree and have performed this analysis in an unbiased manner using a standard genomic binning approach. This analysis is presented in Figure 3a-c and described in the revised section 4 of the Results. This approach allows us to identify the extent of both inter and intra-tumor heterogeneity and reveals variation of both active and repressive chromatin marks at the direct oncogene targets within different cells of the same tumors.

In addition, the authors state that "Discrete sample-specific clusters were resolved by UMAP projection of cells profiled for H3K4me3 and H3K27me3 (Fig. 4b,c) …, indicating that the differences in the H3K4me3 and H3K27me3 landscapes of KMT2Ar leukemia cells of the same samples are generally less than the differences between samples.
But this was completely expected and is due to the initial bias in the annotation for the analysis (see above). The authors have precisely selected their single cell data to only look at regions that differ between samples and not the ones that could differ within one single sample.
Our revised analysis using unbiased binned genomic data described in the previous point address this critique.

Minor concerns:
1/Regarding differences in H3K4me3 between WT and oncogenic sites, could they be due to accessibility issues, could the sites be profiled for accessibility to look into this (H3 profiling, ATAC or other technique)?
We have added profiling of initiating RNA-Pol2, which coincides with accessible sites as we have recently demonstrated (Henikoff S, Henikoff JG, Kaya-Okur HS, Ahmad K. Elife, 2020 9:e63274). These data are presented in Figure 4l and Supplementary Fig. 5.

2/What about batch effect in the single-cell datasets, could it also explain part of the grouping of cells within samples ?
We now provide this analysis in Supplementary Figure 9f-h. This shows that replicate single cell profiling experiments cluster together, thus ruling out batch effects.

Reviewer #3:
Remarks to the Author:
In this manuscript Janssens and colleagues in the Henikoff lab apply a variety of chromatin profiling techniques to characterize a panel of human acute myeloid leukemia (AML) lines and

patient samples. Using automated CUT&RUN and automated CUT&TAG technology the authors profile the binding pattern of the KMT2A fusion oncoprotein as well as a panel of histone modifications using low cell numbers or single cells. The authors confirm previously published results showing that KMT2A fusions display unique localization patterns from non-translocated KMT2A. Translocated KMT2A displays an increased co-localization with the H3K4me1 and H3K36me3 marks relative to wildtype KMT2A. Regulatory element clustering and single cell analysis revealed leukemia subtypes and intra-tumor heterogeneity.

While this article presents a number of technically innovative approaches, it does little to provide actionable mechanistic insight into the pathogenesis of AML that could be used to advance the development of therapies.
We have added a new section (lines 307-342 and Figure 4) of experimental work comparing the sensitivity of different MLL-rearranged leukemias to compounds that target the epigenetic machinery. This analysis reveals that the abundance of Dot1L present at the oncogene binding sites, as defined using AutoCUT&Tag is predictive of whether a sample will be sensitive to Dot1L inhibition. In addition, the spreading of the KMT2A-AF4 fusion lines across the gene body is highly sensitive to Menin inhibition but not Dot1L inhibition.

Moreover, some of the findings here regarding differences in KMT2A fusion binding and have already been reported using standard ChIP-seq approaches (Prange et al., 2017. Oncogene "MLL-AF9 and MLL-AF4 oncofusion proteins bind a distinct enhancer repertoire and target the RUNX1 program in 11q23 acute myeloid leukemia"). Therefore, this manuscript lacks the functional/biological studies that would normally be expected of a publication in Nature Genetics. That said, the work is carefully performed and is of high technical quality making it a good fit for a journal such as Nature Methods.
We believe the improved single cell analyses and addition of drug treatments address the reviewer's concerns of biological importance.

Reviewer comments
1. Defining KMT2A-fusion binding sites as sites detected with the amino-terminal antibody, but not the carboxy-terminal antibody is a reasonable approach, but there are additional controls that would be great to have before being fully convinced of this method. First, it is essential to have more than one KMT2A wildtype cell line used a proof of principle control. The authors use only the H1 human ESC line, how about including human hematopoetic stem cells and non-KMT2A translocated leukemias as controls.
We now include profiling results on CD34+ HSPCs and K562 myeloma cells that both lack the fusion oncoprotein, and profile ML-2 cells that lack the wildtype KMT2A protein (shown in Figure 1c and Supplementary Figures 1-3). Comparisons to these profiles validates our approach for mapping the oncogene protein.

Second, depletion experiment (shRNA or CRISPR) to deplete KMT2A and measure whether detected peaks are specific by loss of CUT&RUN signal is also essential. Using an shRNA specific to the KMT2A fusion partner would be a good way to define oncoprotein specific sites.

Rather than knockdown experiments, we have added experiments using Menin and Dot1L inhibitors which accomplish the same goal of depleting activity.

2. The authors identify a set shared KMT2A oncogene targets including SENP6 and ARID2, which the authors claim are novel dependencies in KMT2A rearranged leukemias based on Cancer Dependency Map (DepMap) data. This is not accurate for SENP6, a common essential gene, whose depletion kills the majority of cancer cell lines in the DepMap database. Similarly, ARID2 is a cancer dependency in many lineages although it does show selectivity for hematopoetic cancers, including AML. However, it does not appear to show a selectivity for KMT2A translocated cancers over other hematopoetic cancers.
We agree, and we have removed text referring to DepMap data, as they were distracting from our main points.

3. Relating to point #2, the authors should perform biological functional assays of potential novel important targets of KMT2A by gene depletion experiments. The manuscript would be greatly strengthened if the authors can demonstrate that their chromatin profiling technologies can point toward new therapeutic targets.
We have added experiments using chromatin profiling to predict responses to treatment with Menin or Dot1L inhibitors. These drugs reduce chromatin modifying activities associated with KMT2A fusions, and are in clinical trials and so have the additional value of therapeutic relevance. This is the newly added section (lines 307-342 and Figure 4) of the Results.

4. The characterization of the SEPT6 translocation is interesting and suggests that perhaps this fusion does not interact with the canonical fusion complexes of KMT2A. This is a novel area that the authors could explore. How does gene expression in the SEPT6 translocated cells compare to canonical KMT2A translocations. Understanding the mechanisms governing the pathogenesis of non-SEC/Dotcom KMT2A translocation driven AML would add novelty to this manuscript.
We agree that characterization of chromatin proteins in the SEPT6 fusion is novel and now examine H3K27ac enrichment at KMT2A-SEPT6 fusion sites. We find the abundance of markers of active transcription are indeed enriched at the oncogene targets (Supplementary Figure 6b), suggesting that the mechanism of KMT2A-SEPT6 transformation may be similar to other fusions, despite relative lack of enrichment of other factors such as Dot1L or ENL at KMT2A-SEPT6 binding sites (Figure 4b, c). We make this point on lines 365-367.

5. With regard to the colocalization of KMT2A fusions with various histone modifications, the association with H3K36me3 (mark of active gene bodies) is not surprising because KMT2A was previously shown to co-localize with H3K79me2, another marker of active gene bodies (Prange et al., 2017). Moreover, KMT2A fusions are most commonly components of transcription elongation complexes that localize to actively transcribed genes.
We thank the reviewer for bringing this to our attention and we now cite Prange et al. 2017 (line 100).

H3K4me1 is a very broadly distributed mark that occurs both inside of genes and in inter-genic regions. Does KMT2A fusion binding only co-localize with with active gene-body associated H3K4me1, or does it bind to silent H3K4me1 marked intergenic elements? If the former, this is likely not a causative mechanism for recruitment of the fusion but is simply a reflection of the very broad distribution of the H3K4me1 mark in active/poised chromatin.
We agree with the reviewer that the colocalization of the oncogene with H3K4me1 may simply reflect the localization of the oncogene across the gene body, and so we have reworded this section to avoid the implication of H3K4 methylation playing a causal role in gene expression and have de-emphasized the boxplots by moving them to Supplementary Figure 6.

Do the authors observe any correlation between KMT2A fusion binding and histone acetylation?
Yes, and we have added results examining both H3K27ac and H4K16ac across the full panel of KMT2A-rearranged samples as well as for CD34+ control cells. We found that H3K27ac (but not H4K16ac) is enriched at KMT2A fusion binding sites compared to KMT2A wildtype control sites in 5/11 samples (Supplementary Figure 5), which is consistent with the known role of KMT2A-fusions in promoting transcriptional activation of its targets.

6. In their final section the authors propose that KMT2A-AF4 leukemias have greater lineage plasticity. The authors should perform functional experiments to test this.
Published work has observed lineage plasticity for KMT2A-AF4 leukemias (References 24 and 25). Our work supports these conclusions. We now cite these precedents (line 376).

Decision Letter, first revision:

27th Apr 2021

Dear Steve,

Your Article, "Automated CUT&Tag profiling of chromatin heterogeneity in mixed-lineage leukemia" has now been seen by 2 of the 3 original referees.

You will see from their comments below that while they continue find your work of interest, some important points are still outstanding. We are interested in the possibility of publishing your study in Nature Genetics, but would like to consider your response to these concerns in the form of a revised manuscript before we make a final decision on publication.

In brief, Reviewer #1 appreciates the improvement shown in this revision. Their requests are mainly presentational, i.e., changes to the text and figures. Reviewer #2 is more critical. They say that the statistical analysis in the manuscript is lacking, and must be made much more rigorously quantitative;

they provide a list of specific examples for 2 pages of the manuscript and suggest this must be checked throughout the text. They also make a comment about bivalency.

We think that these concerns are important and should be completely addressed. We believe that the directions provided are clear and practical, and hope that you and your co-authors will find them helpful when preparing your revision.

To guide the scope of the revisions, the editors discuss the referee reports in detail within the team, including with the chief editor, with a view to identifying key priorities that should be addressed in revision and sometimes overruling referee requests that are deemed beyond the scope of the current study. We hope that you will find the prioritized set of referee points to be useful when revising your study. Please do not hesitate to get in touch if you would like to discuss these issues further.

We therefore invite you to revise your manuscript taking into account all reviewer and editor comments. Please highlight all changes in the manuscript text file. At this stage we will need you to upload a copy of the manuscript in MS Word .docx or similar editable format.

We are committed to providing a fair and constructive peer-review process. Do not hesitate to contact us if there are specific requests from the reviewers that you believe are technically impossible or unlikely to yield a meaningful outcome.

When revising your manuscript:

*1) Include a “Response to referees” document detailing, point-by-point, how you addressed each referee comment. If no action was taken to address a point, you must provide a compelling argument. This response will be sent back to the referees along with the revised manuscript.

*2) If you have not done so already please begin to revise your manuscript so that it conforms to our Article format instructions, available
here.
Refer also to any guidelines provided in this letter.

*3) Include a revised version of any required Reporting Summary:
https://www.nature.com/documents/nr-reporting-summary.pdf
It will be available to referees (and, potentially, statisticians) to aid in their evaluation if the manuscript goes back for peer review.
A revised checklist is essential for re-review of the paper.

Please be aware of our guidelines on digital image standards.

Please use the link below to submit your revised manuscript and related files:

[REDACTED]

Note: This URL links to your confidential home page and associated information about manuscripts you may have submitted, or that you are reviewing for us. If you wish to forward this email to co-authors, please delete the link to your homepage.

We are happy to set a flexible deadline for the receipt of your revised manuscript.

Please do not hesitate to contact me if you have any questions or would like to discuss these revisions further.

Nature Genetics is committed to improving transparency in authorship. As part of our efforts in this direction, we are now requesting that all authors identified as 'corresponding author' on published papers create and link their Open Researcher and Contributor Identifier (ORCID) with their account on the Manuscript Tracking System (MTS), prior to acceptance. ORCID helps the scientific community achieve unambiguous attribution of all scholarly contributions. You can create and link your ORCID from the home page of the MTS by clicking on 'Modify my Springer Nature account'. For more information please visit please visit www.springernature.com/orcid.

We look forward to seeing the revised manuscript and thank you for the opportunity to review your work.

Sincerely,

Michael Fletcher, PhD
Associate Editor, Nature Genetics

ORCID: 0000-0003-1589-7087

Referee expertise:

Referee #1:

Referee #2:

Referee #3:

Reviewers' Comments:

Reviewer #1:
Remarks to the Author:
The manuscript by Janssens et al. et al is substantially changed with multiple new analyses of the previous data, some new studies including the perturbations suggested (drug inhibition studies), and updated text. Comments are organized in 2 sections below, first a response to the specific original critiques and then comments on the current state of the revised manuscript.

1) With respect to the specific original critiques, comments are:
A)
• the addition of the ML2 line and additional referencing adds breadth to this analysis of fusion oncoprotein sites. However, the statement that "this difference becomes more exacerbated at the

fusion oncoprotein binding sites due to a significant depletion of the C-terminal signal" seems a bit overstated and relates to a possible misunderstanding of comment A2. The relative N>C enrichment on oncofusion targets seems just as likely to reflect an increase in actual oncofusion protein on chromatin or the increased breadth that it occupies thus increase in overall signal, rather than any kind of specific depletion of the C terminus. Related to this, the H3K4me3 data on the oncofusion sites (old figure 2A) certainly is not depleted, although this could be interpreted multiple ways. Therefore the authors could still clarify this section to accurately reflect what the data shows (eg is the absolute N- or C-signal comparable between samples or is just the relative enrichment within a sample what can be compared?). I don't think the authors actually addressed the comment A2 in the revised document lines 176-177 but that may not be necessary with the new analyses, however I would strongly encourage more clarity in the interpretation of the "depletion of C terminal signal" statement.
B) revision satisfactory
C)
• The addition of the analysis shown in Fig. 1c/d is actually a very nice improvement to understand the features of the defined binding sites, as is the discussion of bivalent sites as the authors define them. The value of the single cell data as applied to primary samples cannot be overstated, these aspects of the study provide significant new information and goes beyond addressing this comment.
• The drug inhibition studies are a nice addition, but the new analyses illustrating heterogeneity in chromatin states and connecting this to cell identity really bring the best new information, for that reason clarity in these figures are very important and some suggestions follow to improve that a bit

2) With respect to the new manuscript:
• The visualization of lineage-specific clusters in the new Figure 2g makes it challenging to see the relevant populations: yellow-on-white is hard to see and I think it was originally intended to match a heat map which is no longer there. I would favor bringing back the outlining or a red-on-grey approach or inverting the colors to highlight the differences the authors talk about in the text
• It could be made more clear which of these these bulk analyses shown in Figure 2 are based on the indicated chromatin modifications only in "diagnostic immunophenotypic markers" and whether those markers are the same as what are shown in Figure 2b.
• References in the discussion (line 376) that relate to leukemia heterogeneity are 24, 25 which really don't address lineage heterogeneity. Reference 21 however does as do several reviews addressing myeloid vs lymphoid identity in de novo KMT2A-rearranged leukemia and relapses post treatment. These should be swapped out here.
• Also a statement in the discussion at line 376 "our single cell profiling…display at least two gene expression programs" should more accurately be two chromatin states, since expression is not actually what is shown
• Figure 4i is not that informative and could be moved to supplement
• Line 318: it is not quite accurate to talk about AF9 affinity for Dot1L based on genomics data, more accurate would be to better to talk about dependency for the enrichment or recruitment but given this is not a biochemical reaction affinity is not measured here
• Line 323: typo "dprotein"
• I liked the old heatmap from original Figure 3 because it shows how many genes underlie the tsne plots, which now is not as clear about the number of genes underlying the visual representation of heterogeneity. Could those come back or could the marker genes be more clearly listed for each lineage category?
• Line 261 implies that 8 patient samples were used for single cell studies, yet it is 4; rather than saying leukemia samples, primary specimens and cell lines should be used for clarity.

In sum, this study is unique and has a lot to offer the cancer genomics community, particularly if made a bit more clear/accurate.

Reviewer #2:
Remarks to the Author:
If I appreciate the amount of work that has been performed, my major concern about the absence of quantification and statistical testing throughout the manuscript has not been addressed. I had stated that the terminology remained qualitative throughout the manuscript - with examples of terms that should be avoided if present without any quantification - AND that this led to contradictory statements and conclusions with three examples.
If the authors have tried to provide further analysis/data for those three examples and rephrased to avoid conflicting statements, they have not provided any statistical testing. The manuscript remains in qualitative terms throughout, incompatible with publication, especially in the a genomic journal such as Nature Genetics.
For the sake of clarity, I put here below the details for these issues for the first two pages (3-4):

- Line 92: "high reproducibility" should be associated with a calculation of correlation scores and associated p-value between replicates to be qualified as high or significant.
- Line 95: "identical patterns" is only supported by snapshots with no quantification or testing, whereas it should be supported by a genome-wide comparision of binding signal or peak distribution and associated testing.
- Line 101-103: the usage of Gaussian mixture model is not supported by any graphs of distribution of signal and peak size, nor any associated statistics to justify the partition
- Line 104: "numerous" without any precised number, compared to what?
- Line 105: "similar " not justified by any statistical testing
- Lines 109-110: "narrow" and "wide" peaks are not defined by any means +/- sd to quantify their size in base pairs.
- Lines 114/115: "the majority of these sites are wide with a high .. score", with no quantification of what majority means, or wideness or high, in comparison to a control group of peaks with proper statistical testing.
- Lines 118-123/fig 1e: comparisons of peak localization to gene annotation need to be supported by statistical testing, to test whether the differences observed are significant and could therefore have indeed a potential biological relevance
- Lines 124-127/fig 1f: the term "significant" is used, but without any statistical testing to compare the AutoCUT&RUN signal between N-term and C-term KTM2A.

The rest of the text should be revisited with the same methodology to provide rigorous and statistically sound genomic analysis, mandatory to consider any of these conclusions as relevant.

In addition, regarding the "bivalency", I would be more cautious when using this term; in particular line 260 "bivalent histone modifications" is not correct. If the authors have identified loci that are enriched at the population-level for both H3K4me3 and H3K27me3, they have not shown that these histone modifications indeed co-exist in the same cell on the same locus -which is the defition of bivalency - by bulk ChIP-reChIP experiments for example. It could well be that these marks are enriched on these promoters but in different cells, due to epigenomic heterogeneity, which is precisely the subject of interest here.

Reviewer #3:
None

Author Rebuttal, first revision:

Reviewer #1:
Remarks to the Author:
The manuscript by Janssens et al. et al is substantially changed with multiple new analyses of the previous data, some new studies including the perturbations suggested (drug inhibition studies), and updated text. Comments are organized in 2 sections below, first a response to the specific original critiques and then comments on the current state of the revised manuscript.

1) With respect to the specific original critiques, comments are:
A)
• the addition of the ML2 line and additional referencing adds breadth to this analysis of fusion oncoprotein sites. However, the statement that “this difference becomes more exacerbated at the fusion oncoprotein binding sites due to a significant depletion of the C-terminal signal” seems a bit overstated and relates to a possible misunderstanding of comment A2. The relative N>C enrichment on oncofusion targets seems just as likely to reflect an increase in actual oncofusion protein on chromatin or the increased breadth that it occupies thus increase in overall signal, rather than any kind of specific depletion of the C terminus. Related to this, the H3K4me3 data on the oncofusion sites (old figure 2A) certainly is not depleted, although this could be interpreted multiple ways. Therefore the authors could still clarify this section to accurately reflect what the data shows (eg is the absolute N- or C-signal comparable between samples or is just the relative enrichment within a sample what can be compared?). I don’t think the authors actually addressed the comment A2 in the revised document lines 176-177 but that may not be necessary with the new analyses, however I would strongly encourage more clarity in the interpretation of the “depletion of C terminal signal” statement.
Reviewer 1 makes an excellent point, in that the increase in breadth of the signal complicates quantification. We agree that simply adding a comparison to the ML-2 line, which lacks wildtype KMT2A, was insufficient to allow us to conclude that the oncofusion protein displaced the endogenous protein. Please note that we had only addressed this point in response to the initial request by Reviewer 1 to resolve the controversy between Cao et al. 2016 and Liang et al. 2017: “Therefore discussing how the authors interpret their data with respect to this controversy would be helpful.” However, in the absence of a suitable internal control, we depend on a single CDK34+ positive control line as a wildtype standard, whereas we think that much stronger evidence would be required to definitively resolve the controversy. As

this issue is not directly relevant to our use of N- and C-terminal antibodies to identify oncofusion targets, we have removed the assertion that the oncofusion displaces the wildtype protein (lines 139-142).

B) revision satisfactory
C)
• The addition of the analysis shown in Fig. 1c/d is actually a very nice improvement to understand the features of the defined binding sites, as is the discussion of bivalent sites as the authors define them. The value of the single cell data as applied to primary samples cannot be overstated, these aspects of the study provide significant new information and goes beyond addressing this comment.
We thank Reviewer 1 for the initial constructive criticism, which led to a substantial improvement of the manuscript.

• The drug inhibition studies are a nice addition, but the new analyses illustrating heterogeneity in chromatin states and connecting this to cell identity really bring the best new information, for that reason clarity in these figures are very important and some suggestions follow to improve that a bit

2) With respect to the new manuscript:
• The visualization of lineage-specific clusters in the new Figure 2g makes it challenging to see the relevant populations: yellow-on-white is hard to see and I think it was originally intended to match a heat map which is no longer there. I would favor bringing back the outlining or a red-on-grey approach or inverting the colors to highlight the differences the authors talk about in the text
We agree and have now changed the color scheme of the relevant panels to improve visibility.

• It could be made more clear which of these these bulk analyses shown in Figure 2 are based on the indicated chromatin modifications only in “diagnostic immunophenotypic markers” and whether those markers are the same as what are shown in Figure 2b.
In fact Fig. 3b is the only analysis that is limited to the promoters of the diagnositic immunophenotypic markers, and we now clarify this in the figure legend.

• References in the discussion (line 376) that relate to leukemia heterogeneity are 24, 25 which really don’t address lineage heterogeneity. Reference 21 however does as do several reviews addressing myeloid vs lymphoid identity in de novo KMT2A-rearranged leukemia and relapses post treatment. These should be swapped out here.
As suggested we have replaced the previous references with a recent review that succinctly describes models for lineage heterogeneity (line 412).

• Also a statement in the discussion at line 376 “our single cell profiling…display at least two

gene expression programs" should more accurately be two chromatin states, since expression is not actually what is shown
As suggested this has now been changed to "both active and repressive chromatin states" (lines 412-413).

• Figure 4i is not that informative and could be moved to supplement
Done

• Line 318: it is not quite accurate to talk about AF9 affinity for Dot1L based on genomics data, more accurate would be to better to talk about dependency for the enrichment or recruitment but given this is not a biochemical reaction affinity is not measured here
Changed to "the AF9 fusion partner recruits particularly high levels of Dot1L to oncoprotein target loci" (lines 349-350).

• Line 323: typo "dprotein"
Fixed

• I liked the old heatmap from original Figure 3 because it shows how many genes underlie the tsne plots, which now is not as clear about the number of genes underlying the visual representation of heterogeneity. Could those come back or could the marker genes be more clearly listed for each lineage category?
Due to space limitations these heatmaps are now included as part of a revised Supplementary Figure 7, and we now indicate the number of features called for each mark in the main text and figure legend.

• Line 261 implies that 8 patient samples were used for single cell studies, yet it is 4; rather than saying leukemia samples, primary specimens and cell lines should be used for clarity.
As suggested this has now been changed to "four *KMT2A*r cell lines and four primary *KMT2A*r leukemia samples" (lines 290-291).

In sum, this study is unique and has a lot to offer the cancer genomics community, particularly if made a bit more clear/accurate.

Reviewer #2:
Remarks to the Author:
If I appreciate the amount of work that has been performed, my major concern about the absence of quantification and statistical testing throughout the manuscript has not been addressed. I had stated that the terminology remained qualitative throughout the manuscript - with examples of terms that should be avoided if present without any quantification - AND that this led to contradictory statements and conclusions with three examples.
If the authors have tried to provide further analysis/data for those three examples and

rephrased to avoid conflicting statements, they have not provided any statistical testing. The manuscript remains in qualitative terms throughout, incompatible with publication, especially in the a genomic journal such as Nature Genetics.
We thank the reviewer for the thorough and helpful critique, and agree that further statistical analysis was necessary. When appropriate we have now added specific quantifications to the text and/or added additional tabs to Supplementary Table 2. In addition, we have added several additional panels to Supplementary figure 8 to indicate quantitative comparisons of the number of elements labeled by specific marks in our bulk CUT&Tag profiling in each leukemia subtype as well as the CD34+ controls. To further clarify, we now provide brackets and *p*-value indications of relevant comparisons in our box and whisker plots. Finally, the figure legends have been updated to match the reporting requirements of *Nature Genetics*.

For the sake of clarity, I put here below the details for these issues for the first two pages (3-4):

- Line 92: "high reproducibility" should be associated with a calculation of correlation scores and associated p-value between replicates to be qualified as high or significant.
We added "($r = 0.78 \pm 0.19$)" (line 93).

- Line 95: "identical patterns" is only supported by snapshots with no quantification or testing, whereas it should be supported by a genome-wide comparision of binding signal or peak distribution and associated testing.
We added "($r = 0.82 \pm 0.08$)" (line 97).

- Line 101-103: the usage of Gaussian mixture model is not supported by any graphs of distribution of signal and peak size, nor any associated statistics to justify the partition
We added the graphs of distributions and associated statistics as panels to Supplementary Figure 2.

- Line 104: "numerous" without any precised number, compared to what?
We added "131/6336" (line 111).

- Line 105: "similar " not justified by any statistical testing
We now state that a two component Gaussian mixture model failed to partition the KMT2A bound sites in CD34+ HSPCs cells (lines 112-113).

- Lines 109-110: "narrow" and "wide" peaks are not defined by any means +/- sd to quantify their size in base pairs.
Here and below, we now define the wide peaks by means ± sd (line 112) and present Gaussian mixture models and statistics to clarify the difference between wide and

narrow peaks (Supplementary Figure 2a-j)

- Lines 114/115: “the majority of these sites are wide with a high .. score”, with no quantification of what majority means, or wideness or high, in comparison to a control group of peaks with proper statistical testing.

We now define the wide peaks by means ± sd (line 117).

- Lines 118-123/fig 1e: comparisons of peak localization to gene annotation need to be supported by statistical testing, to test whether the differences observed are significant and could therefore have indeed a potential biological relevance

These statistics are provided as additional tabs in Supplementary Table 2.

- Lines 124-127/fig 1f: the term “significant” is used, but without any statistical testing to compare the AutoCUT&RUN signal between N-term and C-term KTM2A.

We added “($p = 3.99 \times 10^{-6}$)” (line 139).

The rest of the text should be revisited with the same methodology to provide rigorous and statistically sound genomic analysis, mandatory to consider any of these conclusions as relevant.
Done.

In addition, regarding the “bivalency”, I would be more cautious when using this term; in particular line 260 “bivalent histone modifications” is not correct. If the authors have identified loci that are enriched at the population-level for both H3K4me3 and H3K27me3, they have not shown that these histone modifications indeed co-exist in the same cell on the same locus -which is the defition of bivalency - by bulk ChIP-reChIP experiments for example. It could well be that these marks are enriched on these promoters but in different cells, due to epigenomic heterogeneity, which is precisely the subject of interest here.
We agree with the reviewer that examining the overlap between H3K4me3 and H3K27me3 in bulk experiments has led to some confusion as to the prevalence of truly “bivalent” nucleosomes. Bivalency was initially defined by bulk analysis (Bernstein et al. 2006), but Bernstein’s group later used single-nucleosome array analysis to demonstrate that only a very small fraction of nucleosomes in regions scored as bivalent have both marks (Shema et al. PMID: 27151869). To avoid ambiguity on this point, we have defined a bivalent promoter as one that is “poised” to indicate that our use of the term bivalency is the traditional one that does not imply that both marks are on the same single nucleosome or the same histone tail (line 271).

Reviewer #3:

None

Decision Letter, second revision:

Our ref: NG-A56051R1

28th May 2021

Dear Steve,

Thank you for submitting your revised manuscript "Automated CUT&Tag profiling of chromatin heterogeneity in mixed-lineage leukemia" (NG-A56051R1). It has now been seen by the original referees and their comments are below. The reviewers find that the paper has improved in revision, and therefore we'll be happy in principle to publish it in Nature Genetics, pending minor revisions to satisfy the referees' final requests and to comply with our editorial and formatting guidelines.

If the current version of your manuscript is in a PDF format, please email us a copy of the file in an editable format (Microsoft Word or LaTex)-- we can not proceed with PDFs at this stage.

We are now performing detailed checks on your paper and will send you a checklist detailing our editorial and formatting requirements in about a week. Please do not upload the final materials and make any revisions until you receive this additional information from us.

Thank you again for your interest in Nature Genetics Please do not hesitate to contact me if you have any questions.

Sincerely,

Michael Fletcher, PhD
Associate Editor, Nature Genetics

ORCID: 0000-0003-1589-7087

Reviewer #1 (Remarks to the Author):

The alterations to this multiply revised manuscript are acceptable; the authors put significant effort into addressing major concerns of 2 reviewers and the text is significantly more transparent and true to the data.

Reviewer #2 (Remarks to the Author):

I am pleased to see that the authors have now performed quantification and statistical testing throughout the manuscript.
No further comments.

Final Decision Letter:

In reply please quote: NG-A56051R2 Henikoff

12th Aug 2021

Dear Steve,

I am delighted to say that your manuscript "Automated CUT&Tag profiling of chromatin heterogeneity in mixed-lineage leukemia" has been accepted for publication in an upcoming issue of Nature Genetics.

Prior to setting your manuscript, we may make minor changes to enhance the lucidity of the text and with reference to our house style. We therefore ask that you examine the proofs most carefully to ensure that we have not inadvertently altered the sense of your text in any way.

Once your manuscript is typeset and you have completed the appropriate grant of rights, you will receive a link to your electronic proof via email with a request to make any corrections within 48 hours. If, when you receive your proof, you cannot meet this deadline, please inform us at rjsproduction@springernature.com immediately.

Your paper will be published online after we receive your corrections and will appear in print in the next available issue. You can find out your date of online publication by contacting the Nature Press Office (press@nature.com) after sending your e-proof corrections. Now is the time to inform your Public Relations or Press Office about your paper, as they might be interested in promoting its publication. This will allow them time to prepare an accurate and satisfactory press release. Include your manuscript tracking number (NG-A56051R2) and the name of the journal, which they will need when they contact our Press Office.

Before your paper is published online, we shall be distributing a press release to news organizations worldwide, which may very well include details of your work. We are happy for your institution or funding agency to prepare its own press release, but it must mention the embargo date and Nature Genetics. Our Press Office may contact you closer to the time of publication, but if you or your Press Office have any enquiries in the meantime, please contact press@nature.com.

Acceptance is conditional on the data in the manuscript not being published elsewhere, or announced in the print or electronic media, until the embargo/publication date. These restrictions are not intended to deter you from presenting your data at academic meetings and conferences, but any enquiries from the media about papers not yet scheduled for publication should be referred to us.

Please note that <i>Nature Genetics</i> is a Transformative Journal (TJ). Authors may publish their research with us through the traditional subscription access route or make their paper immediately open access through payment of an article-processing charge (APC). Authors will not be required to make a final decision about access to their article until it has been accepted. Find out more

about Transformative Journals

<B>Authors may need to take specific actions to achieve compliance with funder and institutional open access mandates. For submissions from January 2021, if your research is supported by a funder that requires immediate open access (e.g. according to Plan S principles) then you should select the gold OA route, and we will direct you to the compliant route where possible. For authors selecting the subscription publication route our standard licensing terms will need to be accepted, including our self-archiving policies. Those standard licensing terms will supersede any other terms that the author or any third party may assert apply to any version of the manuscript.

Please note that Nature Research offers an immediate open access option only for papers that were first submitted after 1 January, 2021.

You will not receive your proofs until the publishing agreement has been received through our system.

If you have any questions about our publishing options, costs, Open Access requirements, or our legal forms, please contact ASJournals@springernature.com

If you have posted a preprint on any preprint server, please ensure that the preprint details are updated with a publication reference, including the DOI and a URL to the published version of the article on the journal website.

To assist our authors in disseminating their research to the broader community, our SharedIt initiative provides you with a unique shareable link that will allow anyone (with or without a subscription) to read the published article. Recipients of the link with a subscription will also be able to download and print the PDF.

As soon as your article is published, you will receive an automated email with your shareable link.

You can now use a single sign-on for all your accounts, view the status of all your manuscript submissions and reviews, access usage statistics for your published articles and download a record of your refereeing activity for the Nature journals.

An online order form for reprints of your paper is available at https://www.nature.com/reprints/author-reprints.html. Please let your coauthors and your institutions' public affairs office know that they are also welcome to order reprints by this method.

If you have not already done so, we invite you to upload the step-by-step protocols used in this manuscript to the Protocols Exchange, part of our on-line web resource, natureprotocols.com. If you complete the upload by the time you receive your manuscript proofs, we can insert links in your article that lead directly to the protocol details. Your protocol will be made freely available upon publication of your paper. By participating in natureprotocols.com, you are enabling researchers to more readily reproduce or adapt the methodology you use. Natureprotocols.com is fully searchable, providing your protocols and paper with increased utility and visibility. Please submit your protocol to https://protocolexchange.researchsquare.com/. After entering your nature.com username and password you will need to enter your manuscript number (NG-A56051R2). Further information can be

found at https://www.nature.com/nprot/.

Sincerely,

Michael Fletcher, PhD
Associate Editor, Nature Genetics

ORCID: 0000-0003-1589-7087
